# Molecular pathology of Usher 1B patient-derived retinal organoids at single cell resolution

**DOI:** 10.1016/j.stemcr.2022.09.006

**Published:** 2022-10-13

**Authors:** Yeh Chwan Leong, Valentina Di Foggia, Hema Pramod, Maria Bitner-Glindzicz, Aara Patel, Jane C. Sowden

**Affiliations:** 1 UCL Great Ormond Street Institute of Child Health, University College London and NIHR Great Ormond Street Hospital Biomedical Research Centre, University College London, London WC1N 1EH, UK

**Keywords:** MYO7A, USH1B, Usher syndrome, retinitis pigmentosa, patient-derived iPSC, retinal organoid, disease modeling, single-cell RNA sequencing, retinal degeneration, oxidative stress, proteasomal ubiquitin-dependent protein catabolic processes, Müller cells, ubiquitin-proteosome system

## Abstract

Usher syndrome-associated retinitis pigmentosa (RP) causes progressive retinal degeneration, which has no cure. The pathomechanism of Usher type 1B (USH1B)-RP caused by *MYO7A* mutation remains elusive because of the lack of faithful animal models and limited knowledge of *MYO7A* function. Here, we analyzed 3D retinal organoids generated from USH1B patient-derived induced pluripotent stem cells. Increased differential gene expression occurred over time without excessive photoreceptor cell death in USH1B organoids compared with controls. Dysregulated genes were enriched first for mitochondrial functions and then proteasomal ubiquitin-dependent protein catabolic processes and RNA splicing. Single-cell RNA sequencing revealed *MYO7A* expression in rod photoreceptor and Müller glial cells corresponding to upregulation of stress responses in *NRL*^*+*^ rods and apoptotic signaling pathways in *VIM*^*+*^ Müller cells, pointing to the defensive mechanisms that mitigate photoreceptor cell death. This first human model for USH1B-RP provides a representation of patient retina *in vivo* relevant for development of therapeutic strategies.

## Highlights

• USH1B patient iPSC-derived retinal organoids contain 7 major retinal cell types
• Bulk RNA-seq identified dysregulated biological processes in USH1B organoids
• Single-cell RNA-seq showed cellular responses to stress in patient-derived rods
• Patient-derived Müller cells were enriched for apoptotic signaling pathways

## Graphical abstract

**Figure:**
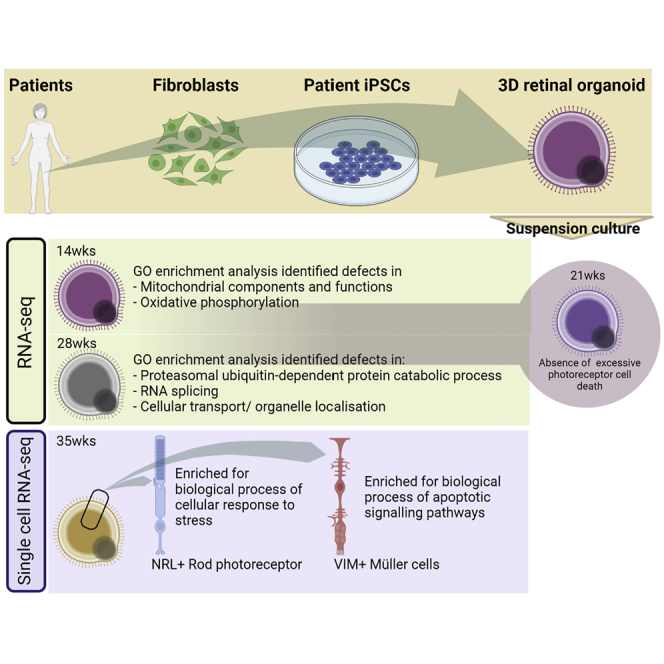

## Teaser

In this article, Leong and colleagues describe the first 3D retinal organoid model for USH1B generated from patient-derived iPSCs. Transcriptional defects informative of disease mechanisms associated with protein ubiquitination and RNA splicing were identified through bulk RNA sequencing. Single-cell transcriptomic analysis confirmed exclusive susceptibility of rods to cellular stress while providing insight into the role of Müller cells in USH1B pathology.

## Introduction

Usher syndrome (USH) is an autosomal-recessive hereditary disease that causes sensorineural hearing loss, vestibular dysfunction, and retinitis pigmentosa (RP). It is the most common hereditary disease that causes deaf-blindness, affecting 1 to 4 in 25,000 newborns (Parker and Bitner-Glindzicz, 2015). Three major USH clinical types (I–III) have been identified. USH1 is the most severe causing profound congenital hearing loss, RP beginning in the first decade of life, and balance issues. RP is characterized by progressive loss of rod photoreceptors in the retina, resulting in night blindness and tunnel vision, followed by cone photoreceptor loss, affecting central vision and eventually leading to legal blindness (Bonnet and El-Amraoui, 2012). *MYO7A* gene mutation (USH1B subtype) accounts for 40%–55% of all USH1 cases, with >2,000 disease-causing variants reported (Whatley et al., 2020). Although hearing loss can be effectively compensated for with cochlear implants, no treatments are currently available for USH1B-RP.

MYO7A is a class 7 myosin protein with an N-terminal motor domain and distinct properties (Heissler and Manstein, 2012). Like other members of the myosin superfamily, it binds actin filaments triggering ATP hydrolysis to energise its translocation. No faithful animal or human cell models of USH1B-associated RP are available to date. *Myo7a* mutant mouse models, such as *shaker1*, recapitulate hearing loss and vestibular dysfunction but not the USH1B retinal phenotype (Gibson et al., 1995). Studies using *shaker1* identified rhodopsin mislocalization and showed MYO7A in the connecting cilium along the axonemal actin, where it facilitates rhodopsin transport from the photoreceptor inner segment (IS) to the outer segment (OS) (Liu et al., 1999). The same model showed the absence of MYO7A disrupted melanosome translocation to the apical processes of retinal pigment epithelium (RPE) cells and OS phagocytosis (Gibbs et al., 2003). Soni et al. (2005) demonstrated disrupted interaction of MYO7A with lysosomes in a human RPE cell line. In addition, contrasting findings of RPE resistance (Lopes et al., 2011) and rod vulnerability (Peng et al., 2011) to light exposure were reported in *shaker1*.

Subsequent studies in humans, non-human primates, and amphibians showed MYO7A and other USH1 proteins form a linkage between the OS and calyceal processes (CP), the actin filament-filled extensions sprouting from the IS to surround the base of OS (Sahly et al., 2012). Knockout of protocadherin-15 (USH1F) and cadherin-23 (USH1D) in frogs compromised the CP structural support, causing distortion of the OS shape (Schietroma et al., 2017). Notably, *Myo7a* knockout frogs acquired developmental defects hampering further study. The lack of CP in the mouse retina plus other differences compared with the human eye, such as eye size, high rod-to-cone ratio, and the absence of a macula could explain the absence of a USH1B retinal phenotype in mouse models. A study in dogs also failed to observe retinal phenotypes (Webb et al., 2019), while vestibular dysfunction and deafness were present. Pigs with *MYO7A* mutation showed balance difficulties and suspected hearing loss, but retinal phenotypes were not studied (Derks et al., 2021). Like the frog model, piglets with a homozygous nonsense *MYO7A* mutation died soon after birth.

In addition to the lack of faithful animal models, there is a wider lack of understanding of the molecular pathways leading to photoreceptor cell death that are triggered by the disease-causing mutations. Modeling USH1B with patient-derived induced pluripotent stem cells (iPSCs) offers an alternative route with potential to understand the early consequences of *MYO7A* mutation in human retinal cells. Tang et al. (2016) reported the first human USH1B cochlear hair cell model derived from iPSCs of a patient suffering sensorineural hearing loss (non-syndromic USH1B), but retinal cells have not previously been studied. Rod photoreceptor cells are highly metabolically active requiring high oxygen consumption. In RP, elevated oxidative stress is thought to accelerate rod photoreceptor cell loss and secondary cone photoreceptor death. The sensitivity of photopigments to light stress and the high turnover of OS contribute to the production and accumulation of reactive oxygen species (ROS), leading to lipid, protein and DNA damage in animal models (Punzo et al., 2012; Gallenga et al., 2021). In addition, antioxidants slow photoreceptor cell death in animal models (Komeima et al., 2006). Increased markers of oxidative stress, including elevation of endoplasmic reticulum (ER) stress markers, have been reported in non-USH1 RP patient iPSC-derived retinal cells grown in two-dimensional (2D) cultures (Jin et al., 2011, 2012; Yoshida et al., 2014) or in organoids (Tucker et al., 2013).

Here, we report the first three-dimensional (3D) retinal organoid model of USH1B generated from patient-derived iPSCs. The iPSC-derived organoids did not show evidence of cell degeneration in culture at fetal retina-equivalent stage, consistent with the onset of USH1B in childhood. However, bulk RNA sequencing (RNA-seq) and Gene Ontology (GO) enrichment analysis revealed increased aberrantly expressed genes in patient-derived cells over time. Single-cell RNA sequencing (scRNA-seq) analysis of 35 week organoids identified adaptive responses to stress specifically in USH1B rods, and enrichment for apoptotic signaling pathways in USH1B Müller cells. Together these findings indicate the presence of a mutation-induced molecular pathology in patient-derived retinal cells and provide evidence of increased adaptive responses in patient cells that maintain cellular homeostasis and prevent photoceptor cell death in the *in vitro* organoid system.

## Results

### Derivation of USH1B patient iPSCs

Dermal skin fibroblasts were collected from three USH1B patients for reprogramming to iPSCs. We confirmed that the generated USH1B-iPSCs harbored the same *MYO7A* mutations identified in their fibroblast counterparts using Sanger sequencing (Figure S1). Patient 2 (USH1B.2) and patient 3 (USH1B.3) are siblings, both harboring a c.6070C>T (p.Arg2024^∗^) nonsense mutation and a c.223G>C (p.Asp75His) missense mutation. Patient 1 (USH1B.1) harbored a c.1996C>T (p.Arg666^∗^) nonsense mutation and an acceptor splice site mutation, c.133-2A>G, in intron 3 (IVS3-2A-G) p.(=), causing the skipping of canonical acceptor splice site and shortening of exon 4 by seven highly conserved amino acids (Figure S1). All iPSCs showed characteristics of pluripotent cells in culture and expressed pluripotency markers as shown by immunohistochemistry (IHC), while maintaining genome integrity as indicated by SNP array (Figure S2).

### Generation of 3D retinal organoids from control and USH1B iPSCs

**Figure 1:**
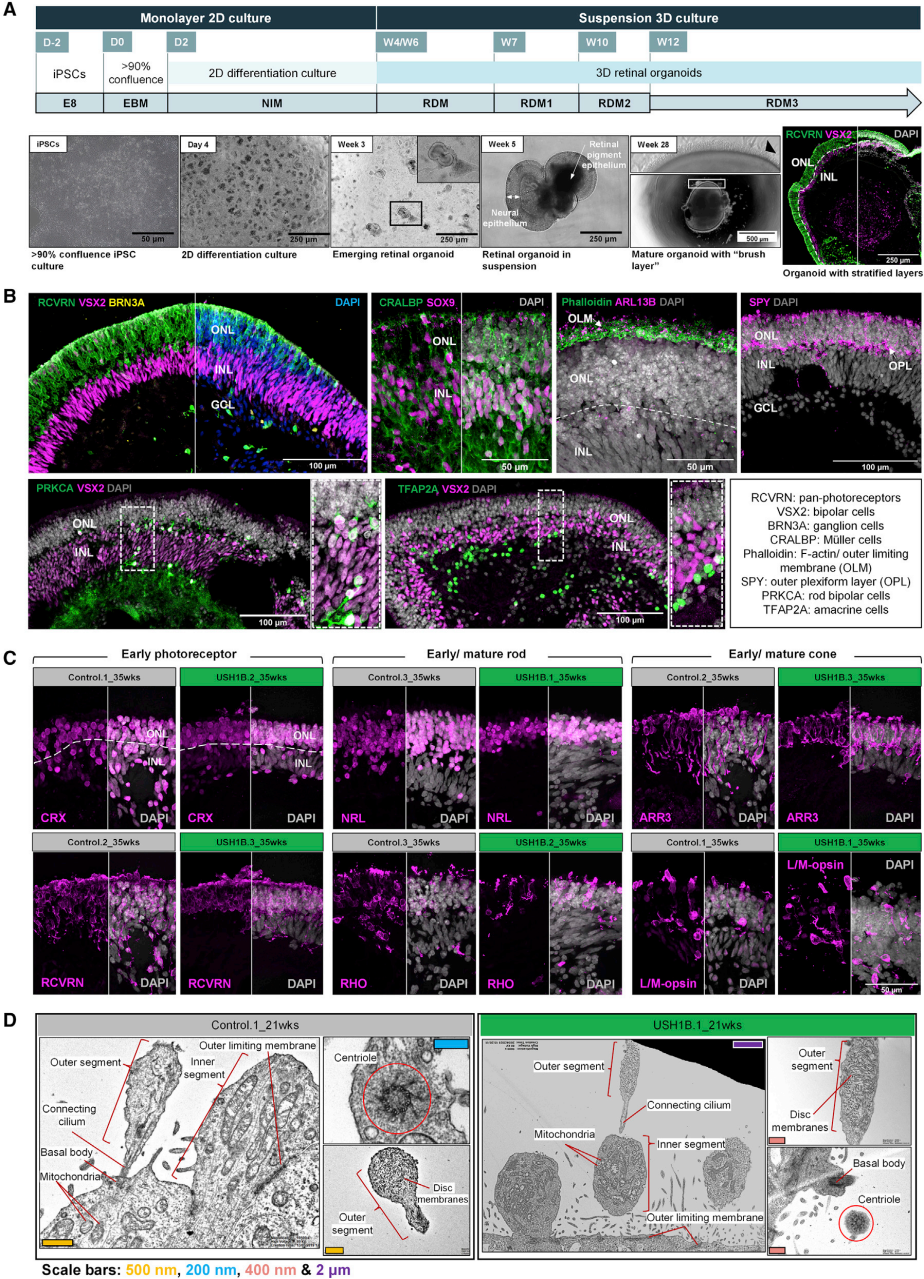
Generation of 3D retinal organoids from USH1B patient and control iPSCs (A) Schematic of retinal differentiation protocol (upper panel). Bright-field images show the appearance of organoid cultures at different stages (lower panel). Black arrowhead, segment-like structures. Representative examples from Control.1 and USH1B.2. (B and C) IHC of 21 and 35 week retinal organoids shows the presence of various retinal cell types and proper cytoarchitecture (B) and early and late photoreceptor markers (C); photoreceptors, RCVRN; bipolar cells/retinal progenitor cells, VSX2; BRN3A, retinal ganglion cells; Müller cells, SOX9 and VIM; phalloidin, outer limiting membrane (OLM); ARL13B, connecting cilium; SYP, inner plexiform layer (INL); PKCα, bipolar cells (see Table S2 for full protein names). Representative examples shown from N > 3 differentiations per iPSC line, n > 3 organoids per differentiation. RPE, retinal pigment epithelium; NE, neural epithelium; ONL, outer nuclear layer; INL, inner nuclear layer; GCL, ganglion cell layer; OLM, outer limiting membrane. (D) Transmission electron micrographs show the presence of photoreceptor subcellular structures and separating photoreceptor cell body and segments in both control and patient 21 week retinal organoids. N = 2 differentiations per iPSC line, n > 2 organoids per differentiation.

USH1B and control iPSCs were differentiated to 3D retinal organoids *in vitro* using a published protocol (Cuevas et al., 2021; Gonzalez-Cordero et al., 2017) (Figure 1A). By 21 weeks, organoids contained stratified neuroepithelium with multiple cell layers that were characterized using IHC (Figure 1B). Photoreceptors in the outer nuclear layer (ONL) stained for RCVRN. The inner nuclear layer (INL) stained for VSX2, marking bipolar cells (BCs), and the ganglion cell layer (GCL) was identified by BRN3A. Müller cells (MCs) were identified using IHC for CRALBP and SOX9. F-actin (phalloidin) staining confirmed the presence of outer limiting membrane (OLM) and the outer apical polarity of the organoid. Synaptophysin (SYP) staining indicated the outer plexiform layer (OPL). Rod BCs (PRKCA) and amacrine cells (ACs) (TFAP2A) were also present. At 35 weeks, both control and USH1B photoreceptors expressed pan-photoreceptor (RCVRN and CRX), rod (NRL), and cone (ARR3) markers, as well as rod-specific (RHO) and cone-specific (L/M-opsin) photopigments (Figure 1C). Photoreceptors acquired subcellular features, such as connecting cilium (ARL13B and PCNT), and rod (PRPH2) or cone (GNAT2) outer segment structures by 28 weeks of differentiation (Figure S2). Transmission electron micrographs of organoids showed the presence of OLM, mitochondria-rich IS, developing OS containing disc membrane-like structures, connecting cilia, basal body and centrioles (with 9 + 0 microtubule arrangement) (Figure 1D). Overall, USH1B iPSCs differentiated as effectively as control iPSCs to produce retinal organoids that resembled native human retina.

### Absence of RP degenerative features in USH1B retinal organoids

**Figure 2:**
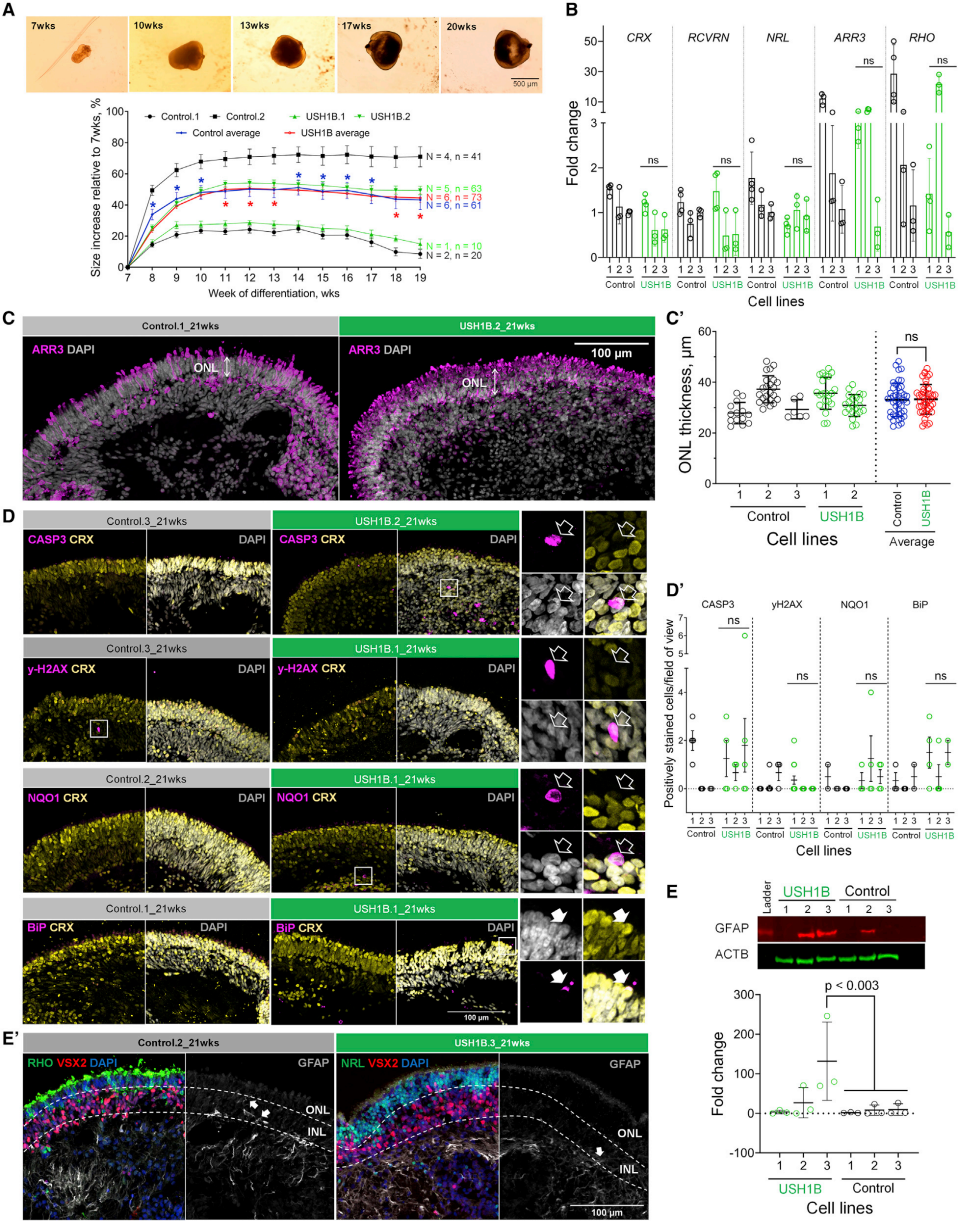
Comparison of USH1B and control retinal organoids to identify USH1B-associated degenerative features (A) Top: bright-field images shows organoid growth; images from Control.1. Changes in organoid size over time relative to 7 weeks. Independent-samples t test was performed at each time point. ^∗^p < 0.001, control > USH1B when in blue, USH1B > control when in red. (B) qRT-PCR analysis of photoreceptor marker expression by 21 week patient and control organoids. One-way ANOVA with Dunnett correction. Fold change relative to *GAPDH* expression and normalized to Control.3. N = 3, n = 2–3. (C and C′) Quantification of ONL thickness in 21 week USH1B and control organoids. Representative images show one field of view. IHC for ARR3 defined the ONL. N = 2, n = 2. Data points are measurements from one field of view. Independent-samples t test. (D) Oxidative stress marker expression in 21 week organoids. Representative images showing one field of view. Insets show colocalization of stress markers with CRX (empty arrow, not colocalized; filled arrow, colocalized). (D′) Quantification of double-positive cells for CRX and stress markers. Data point, number of organoids. N = 2. One-way ANOVA with Dunnett correction. (E) GFAP expression in 21 week organoids. Quantification of GFAP expression with immunoblotting; fold change relative to ACTB expression and normalized to Control.1. N = 3, n = 5–8. (E′) Representative images of IHC of GFAP, with RHO, VSX2, and NRL. Observed in >3 organoids per iPSC line. In (A), bars denote mean ± SEM; in (B)–(E), bars denote mean ± SD. N, independent differentiation per iPSC line; n, organoids per differentiation; ns, not significant.

To determine if USH1B organoids reproduce degenerative phenotypes in patients, first, we compared the size of USH1B and control organoids from 7 to 19 weeks. Using 7 weeks as a baseline we found that although control and USH1B organoid size changed at variable rates, no USH1B-specific abnormality was observed (Figure 2A). To assess whether USH1B organoids lost photoreceptors to degeneration by 21 weeks, we determined the level of photoreceptor marker gene expression and quantified the thickness of the ONL (where photoreceptor nuclei reside). No significant differences were observed in expression of *CRX*, *RCVRN*, *NRL*, *ARR3* and *RHO* (Figure 2B). Similarly, we found no aberrant ONL thinning in USH1B organoids, suggesting excessive photoreceptor death was not present (Figures 2C and 2C′).

To assess if USH1B organoids showed increased cellular stress, we analyzed expression of γH2AX and 8-hydroxydeoxyguanosine (8-OHdG) (DNA damage/stress), NQO1 (oxidative stress), BiP (ER stress), acrolein (lipid oxidation), and CASP3 (apoptosis) using IHC (Figure 2D). Each marker was co-stained with CRX. Overall, stress markers were scarce. When present, they rarely colocalized with CRX, suggesting that stressed cells were not photoreceptors. No significant elevation was observed in USH1B organoids (Figure 2D′). We did not detect any cells positive for 8-OHdG and acrolein (data not shown).

Reactive gliosis is a secondary event to photoreceptor loss in RP, characterized by overexpression of glial fibrillary acidic protein (GFAP) and the disruption of OLM integrity. It was reported in iPSC-derived retinal organoids from RP patients harboring *RPGR* mutation (Deng et al., 2018) and in an *Rpgr*^−/−^ mouse model (Megaw et al., 2017). We quantified GFAP protein levels using western blot and distribution using IHC of 21 week organoids (Figures 2E and 2E′). One patient line (USH1B.3) showed a significantly higher level of GFAP. However, IHC revealed restricted expression of GFAP within inner retina (at the pseudo-GCL and occasionally INL), resembling the pattern in healthy mouse retina (Hippert et al., 2021), in both control and USH1B organoids (including USH1B.3). Overall, USH1B patient organoids did not display marked characteristics of degeneration, photoreceptor cell death, oxidative stress or reactive gliosis.

### RNA sequencing analysis showed resemblance of late-stage retinal organoids to fetal retina

**Figure 3:**
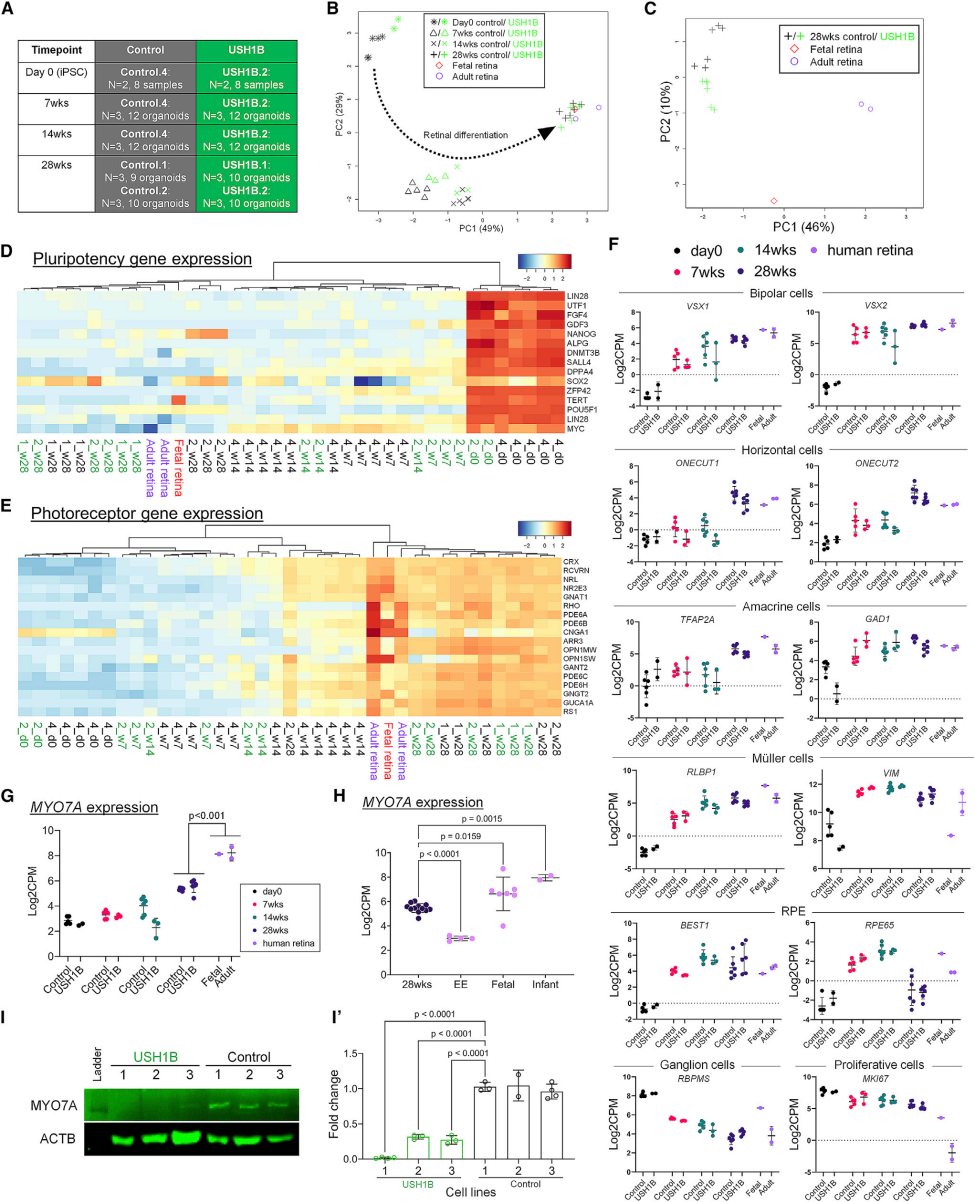
RNA-seq analysis showed similarity of 28 week retinal organoid to fetal retina (A) The number of organoids collected at four different time points of retinal differentiation of USH1B and control iPSC lines: day 0, 7 weeks, 14 weeks, and 28 weeks, for bulk RNA-seq. (B and C) PCA shows similarity of transcriptomes of organoids and human retina. Dotted arrow indicates the path of retinal differentiation. (D and E) Heatmap of pluripotency (D) and photoreceptor (E) marker gene expression in organoids and human retina; control, black; USH1B, green. (F) Gene expression of markers of specific retinal cell types indicated, bipolar, horizontal, amacrine, Müller, retinal ganglion, RPE, and proliferative cells. (G) Expression of *MYO7A* in organoids at different time points and in human retina. (H) Expression of *MYO7A* in 28 weeks organoids compared with human embryonic eye (EE), fetal and infant retina (GEO: GSE98370). (I and I′) Western blot of MYO7A in 28 weeks organoids. Fold change shown relative to ACTB expression and normalized to Control.1. MYO7A, 254 kDa; ACTB, 42 kDa. N = 3 independent differentiations per line; n = 5–8 organoids per differentiation. In (F)–(I), mean fold change or mean log_2_CPM ± SD; one-way ANOVA with Dunnett correction.

To investigate the development of retinal organoids and the “closeness” of their global transcriptomes to native retina, we performed bulk RNA sequencing analysis on control and USH1B iPSCs (day 0), early-stage (7 weeks), mid-stage (14 weeks), and late-stage (28 weeks) organoids, and human 20 weeks post-conception fetal and adult retina (Figure 3A). Principal-component analysis (PCA) revealed close correlation of organoids according to their respective time of differentiation (Figure 3B). Day 0 iPSCs clustered furthest from the fetal and adult retina along PC1, followed by 7 and 14 weeks, indicating the progression of retinal differentiation. All 28 weeks, organoids clustered closer to fetal than to adult retina (Figure 3C). Pluripotency gene expression showed a drastic downregulation in 7, 14, and 28 weeks organoids compared with day 0 iPSCs, indicating loss of the pluripotency state (Figure 3D). The expression of well-defined photoreceptor markers was upregulated starting at 14 weeks and further elevated at 28 weeks to resemble the level of human retina (Figure 3E).

A gradual increase of expression was also observed in marker genes of other retinal cell types, including BCs, horizontal cells (HCs), ACs, and MCs, in the organoids (Figure 3F). Decreased *RBPMS* expression over time signaled diminishing RGC number, consistent with the trajectory in human retina and other retinal organoid studies (Cowan et al., 2020). Varying levels of *BEST1* and *RPE65* were due to the sporadic presence of RPE. Analysis of *MYO7A* expression at different stages showed levels increased concurrently with retinal maturation but were significantly lower than human retina (Figure 3G).

To more accurately map the 28 weeks organoids to an *in vivo* developmental stage, we compared the 28 weeks organoids with a published bulk RNA-seq dataset of human embryonic eye, fetal, and infant retina (Table S3) (Mellough et al., 2019). Figure S3 shows that the 28 weeks organoids were more mature than embryonic eye and were similar to fetal/infant retina with respect to retinal cell marker expression. *MYO7A* expression showed a similar upward trend across *in vivo* developmental stages (Figure 3H), with higher levels in 28 weeks organoids compared with embryonic eyes.

As difference in *MYO7A* mRNA level was not apparent between USH1B and control organoids, we sought to investigate MYO7A protein levels in order to validate the predicted effect of *MYO7A* mutations on protein synthesis. Immunoblotting of 21 weeks organoids (Figures 3I and 3I′) with an antibody (DSHB 138-1) that targets amino acids 927–1203 of MYO7A should detect products from both alleles (if translated; p.Asp75His, p.Arg2024^∗^) of USH1B.2 and USH1B.3 but only one allele (splice site mutation c.133-2A>G) of USH1B.1 (Figure S3). Residual MYO7A expression (fold change 0.30 ± 0.05) was detected in USH1B.2 and USH1B.3 by the p.Asp75His allele, while mRNA products from the p.Arg2024^∗^ allele were not detected suggesting nonsense-mediated mRNA decay. The USH1B.1 allele did not give rise to detectable protein.

To summarize, we showed that USH1B and control organoids developed and matured to acquire global transcriptomes at 28 weeks, largely similar to fetal retina. Profiles of expression of retinal cell markers were similar to adult retina suggesting completion of retinal histogenesis. Considering the onset of USH1B in childhood, the similarity to fetal retina is consistent with the absence of signs of photoreceptor cell degeneration in the USH1B organoids.

### RNA-seq analysis revealed dysregulated GO terms in mid- and late-stage USH1B retinal organoids

**Figure 4:**
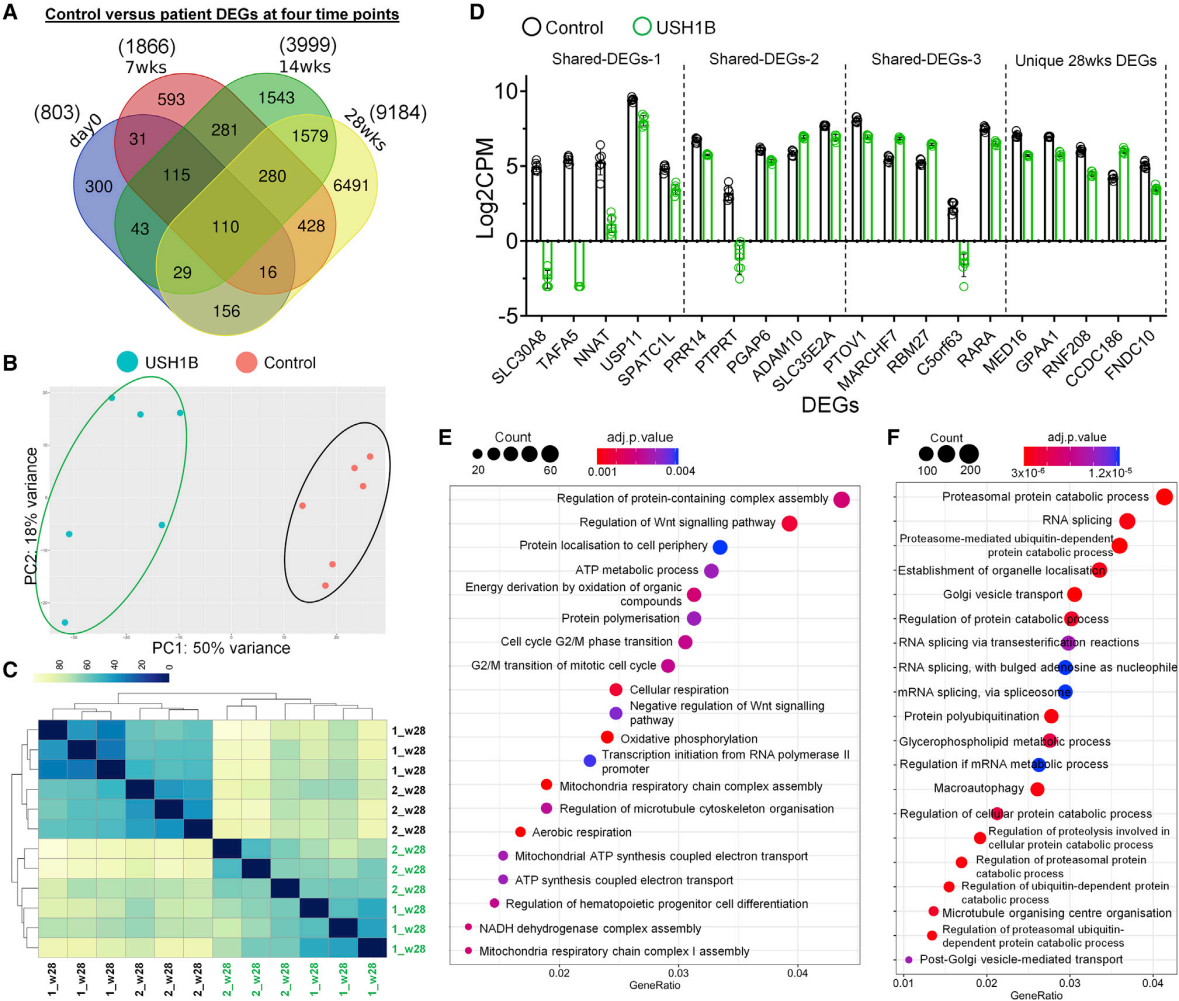
Differential expression analysis comparing control and USH1B iPSCs, 7, 14, and 28 week retinal organoids (A) Venn diagram shows number of significant (adjusted p value < 0.05) DEGs between control and patient at four different time points and number of overlapped DEGs. (B and C) PCA (B) and heatmap of Pearson’s correlation coefficient matrix (C) (control in black and USH1B in green) indicate clear separation of control and patient 28 weeks retinal organoids on the basis of global transcriptional profiles. (D) The expression of the top five DEGs (ranked by adjusted p value) in four overlapped gene lists log_2_CPM ± SD. (E and F) Top 20 most significantly enriched (by adjusted p value) GO biological processes terms analyzing (E) shared DEGs-3 and (F) 28 weeks unique DEGs.

To identify mutation-induced transcriptomic defects in USH1B organoids, we performed differential expression (DE) analysis comparing the transcriptomes of control and USH1B organoids (control versus USH1B). A total of 803, 1,866, 3,999, and 9,184 differentially expressed genes (DEGs) (adjusted p value < 0.05) were identified at day 0, 7 weeks, 14 weeks, and 28 weeks, respectively (Figure 4A; Table S4), suggesting that control and USH1B organoids became more different over time, coinciding with the rise of *MYO7A* expression. Indeed, PCA and Pearson’s correlation coefficient showed clear separation of control and USH1B 28 week organoids (Figures 4B and 4C). Considering RP is progressive, we hypothesised that aberrant transcriptional patterns, if present, should persist, if not increase, at later time points. Therefore, to investigate the onset of molecular pathology associated with *MYO7A* mutation preceding degeneration, we performed GO enrichment analysis on DEGs shared across (1) all four time points (110 DEGs), (2) 7 weeks onward (280 DEGs), (3) 14 week onward (1,579 DEGs), and (4) unique DEGs for 28 weeks (6,491 DEGs), hereafter referred to as shared DEGs-1 to DEGs-3 and 28 weeks unique DEGs (Figure 4A; Table S4). The top five DEGs for each group are shown in Figure 4D (Table S4 indicates links of these top five genes to retina and/or neurodegeneration).

No GO enrichment (q < 0.05) was identified in shared DEGs-1 and shared DEGs-2 (Table S4). Shared DEGs-3 showed enrichment for biological process terms chiefly relating to mitochondria functions, Wnt signaling pathway, protein localization, and cell cycle (Figure 4E). DEGs unique to 28 weeks were enriched for protein ubiquitination, RNA splicing, cellular transport, and organelle localization (Figure 4F; Table S4). Both the latter are relevant to the current understandings of MYO7A function. Consistently, GO enrichment analysis of molecular function terms identified similar findings as shown in Table S4. Ubiquitination is a critical pathway controlling proteosome homeostasis and regulating degradation of damaged or misfolded proteins, and its dysfunction is associated with a range of neurodegenerative diseases that affect the retina (Campello et al., 2013). Figure S4 shows the set of genes in the Kyoto Encyclopedia of Genes and Genomes (KEGG) ubiquitin-mediated proteolysis pathway that are dysregulated in the 28 weeks organoids.

In summary, through bulk RNA-seq analysis, we identified dysregulated genes in USH1B organoids, many of which have implications in retinal dystrophy or other neurodegenerative diseases. These aberrant transcriptomic patterns represent clues for the dissection of USH1B disease mechanisms and serve as potential early disease biomarkers.

### Single-cell RNA sequencing showed the expression of Usher genes at a single-cell level

**Figure 5:**
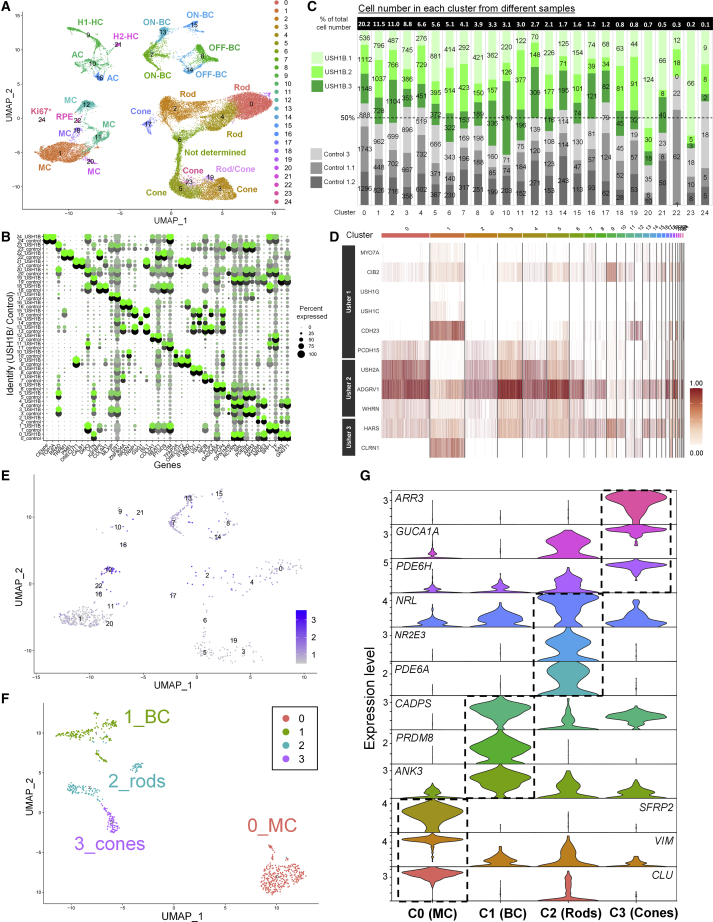
Single-cell RNA sequencing of control and USH1B patient 35 weeks retinal organoids (A) UMAP shows the presence of 25 clusters, encompassing 7 major retinal cell types on the basis of the transcriptomes of 39,934 single cells. (B) Dot plot illustrates the expression of the top two markers (by adjusted p value) by each cluster. Table S5 provides the top genes in each cluster. (C) Bar plot represents the number of cells from each organoid sample in each single-cell cluster. (D) Heatmap shows the expression of the known Usher syndrome causative genes across 25 clusters. (E–G) UMAPs show the distribution of 756 *MYO7A*^*+*^ single cells across 25 clusters identified in Figure 5A (E); re-clustering of these 756 cells identified four distinct clusters, Müller cells (MCs), bipolar cells (BCs), rods and cones (F); violin plots show the expression of the top three markers (dashed boxes) by each of the four clusters (G).

We next sought to elucidate the site of action of *MYO7A* by establishing a cell atlas of USH1B organoids. The 35 weeks USH1B and control organoids were subjected to single-cell RNA sequencing. The 35 weeks time point was chosen because a recent study showed that organoids acquired a stable “developed state” between 30 and 38 weeks and did not mature further (Cowan et al., 2020). The RNA of 19,399 and 20,535 single cells from control and USH1B, respectively, was sequenced. Dimension reduction of the 39,934 single-cell transcriptomes with uniform manifold approximation and projection (UMAP) identified 25 clusters, encompassing 7 major retinal cell types, including rods, cones, BCs, ACs, HCs, MCs, and RPE (Figure 5A), assigned on the basis of the expression of well-defined markers (Figure 5B, Table S5). Further analyses showed the presence of cell subtypes, including rod subtypes differentiated by the expression of *MYO9A*, and BC and HC subtypes (Yi et al., 2021) (Figure S5). Each of the 25 clusters was well represented in the six organoid samples (Figure 5C).

Next, we mapped the expression of all USH-causative genes to organoids (Figure 5D). For USH1 genes, *MYO7A*, *USH1G*, *USH1C*, and *CDH23* were highly expressed by MCs (cluster 1), while *CIB2* was ubiquitously expressed and *PCDH15* specific to photoreceptors. For USH2 genes, high *USH2A* expression was detected in photoreceptors, while *ADGRV1* and *WHRN* were detected in all cell types. Last, USH3 gene *CLRN1* was highly expressed in MCs and *HARS* was ubiquitously expressed. To further determine identities of cells expressing *MYO7A*, we performed dimension reduction on a total of 756 *MYO7A*-expressing cells (Figures 5E and 5F; Table S5). Four distinct cell clusters were identified expressing top markers indicative of MCs, BCs, rods, and cones (Figure 5G; Table S5). Despite previous *MYO7A* studies showing exclusive expression by RPE and photoreceptors, in particular rods, the expression of *MYO7A* by MCs reported in this study is consistent with a recent scRNA-seq analysis of human retina (Cowan et al., 2020). These findings indicate *MYO7A* could play roles in other cell types, which are undocumented and could contribute to pathology.

### Single-cell DE analysis identified early signs of degeneration specific to rods and upregulation of apoptotic pathways in MC

**Figure 6:**
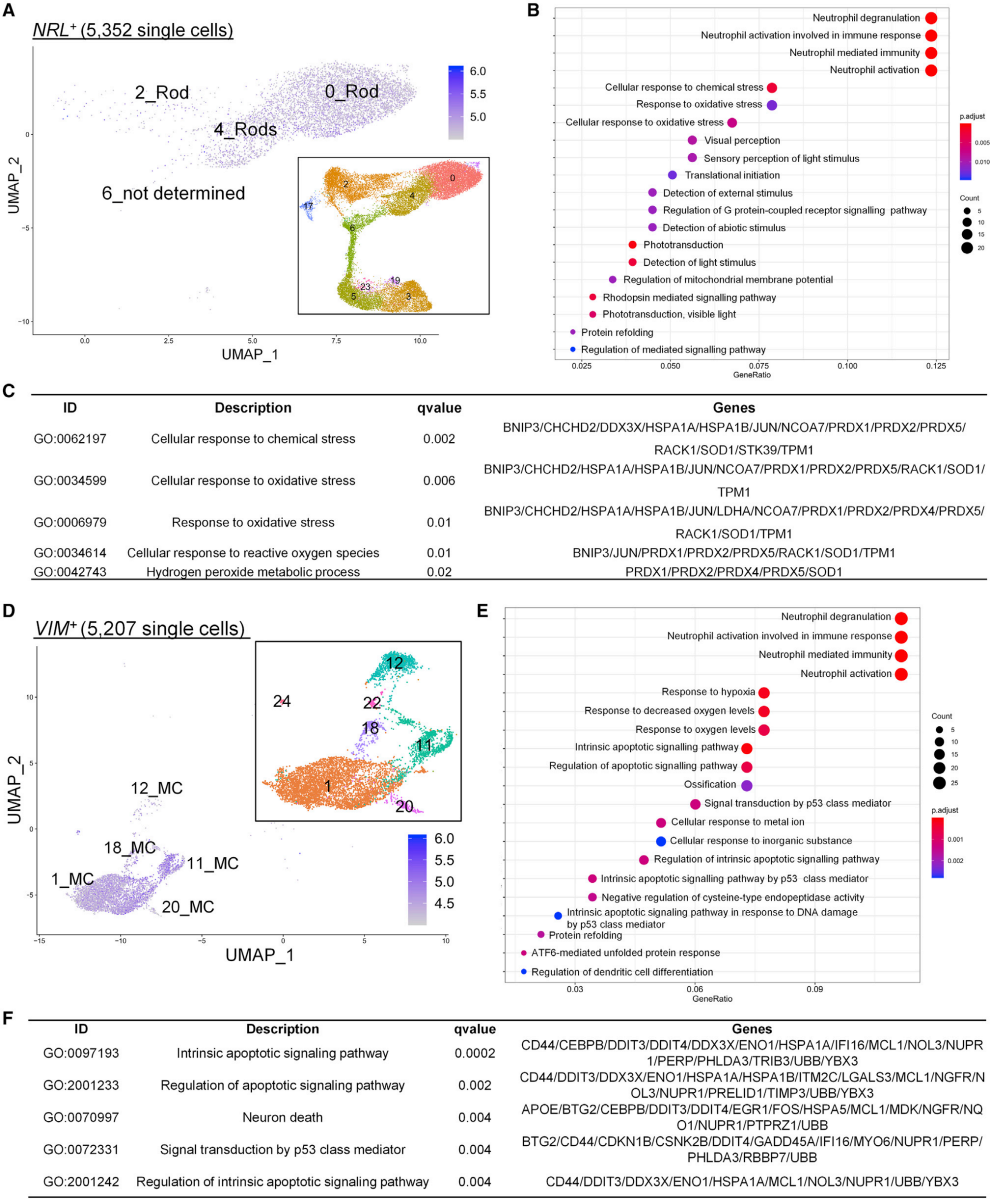
Single-cell RNA-seq identified early signs of degeneration in rods and Müller cells (A) UMAP shows distribution of 5,352 *NRL*^*+*^ cells only in the clusters in the “photoreceptor island” (mostly rod clusters) identified in Figure 5A (inset). (B) Dot plot shows top 20 significantly enriched GO biological processes terms on the basis of analysis of 207 DEGs comparing control and *NRL*^*+*^ cells. (C) Table shows details of five significantly enriched biological process GO terms associated with stress response ranked by adjusted p value Table S5. (D) UMAP shows localization of 5,207 *VIM*^*+*^ cells in the clusters identified as MC in Figure 5A (inset). (E) Dot plot shows top 20 significantly enriched biological processes GO terms on the basis of analysis of 255 DEGs comparing control and USH1B *VIM*^*+*^ cells. (F) Table shows details of the top five significantly enriched GO biological processes terms associated with apoptotic signaling (ranked by adjusted p value) Table S5.

Finally, we sought to identify molecular defects specific to individual retinal cell types. Degeneration of rods is the primary clinical event in RP, hence we examined directly if rods show evidence of altered transcriptomes. NRL is an essential rod-specific transcription factor. We compared the transcriptomes of 3,072 and 2,325 *NRL*-expressing (*NRL*^*+*^) rods from control and USH1B organoids, respectively. All cells were found in clusters 0, 2 and 4, previously identified as rods (Figure 6A; Table S5). DE analysis identified 207 DEGs (Table S5; genes with links to retina and/or neurodegeneration are indicated). GO enrichment analysis identified biological process terms associated with cellular responses to chemical and oxidative stress as well as hydrogen peroxide metabolic process (Figures 6B and 6C; Table S5). Genes encoding pro-apoptotic factor (*BNIP3*), antioxidant enzymes (*PRDX1*, *PRDX2*, and *PRDX5*), and free radical scavenging enzyme (*SOD1*) were overexpressed in USH1B *NRL*^*+*^ cells, suggesting ongoing adaptive responses to cell stress (Figure 6C). The same events were not detected when analyzing 3,013 *ARR3*^*+*^ (cone arrestin) cones, suggesting that molecular pathology was limited to rods (Figures S6A–S6C; Table S5).

In view of the new interest in MCs as a *MYO7A*-expressing cell type, we performed the same analysis on a total of 5,207 vimentin (*VIM*^*+*^) MCs (2,392 control and 2,815 USH1B) (Figures 6D–6F; Table S5). DE analysis identified 255 DEGs, which were enriched for biological process terms for regulation of apoptotic signaling pathways (Table S5). Last, we analyzed 2,277 BCs identified by expression of *GRIK1* (1,136 cells) and *PCP2* (1,141 cells) (Figures S6D–S6F). A total of 157 DEGs were identified, which were not enriched in either apoptotic- or stress response-related GO terms (Table S5). We noted that the abundant presence of ribosomal genes and ribosome-related GO terms in the *ARR3*^*+*^ and *PCP2*^*+*^/*GRIK1*^*+*^ cell-specific GO enrichment analyses might represent technical artifacts previously reported as common to scRNA-seq (Nowakowski et al., 2017).

In summary, we identified stress responses in rods, but not cones, consistent with clinical observations that the former is first to degenerate in USH1B-RP. Analysis of *VIM*^*+*^ MCs showed USH1B MC exhibited apoptotic pathway-related transcription activities. These results suggest our organoid model reproduces a pre-death mutation-induced state of rod photoreceptors *in vitro*. It is also instrumental for further investigation into the vulnerability of MC.

## Discussion

By coupling iPSC technology and *in vitro* retinal differentiation protocols, we established the first retinal organoid disease model for USH1B. Bulk RNA-seq analysis revealed the transcriptomes of USH1B patient organoids were progressively distinct from controls after extended time in culture, coinciding with the gradual rise of *MYO7A* expression. Dysregulation of genes associated with mitochondrial function were followed by genes enriched for protein ubiquitination, RNA splicing, and cellular transport functions. Elevated cellular responses to stress were detected exclusively in USH1B rods, suggesting organoids were modeling a molecular pathology underlying early RP manifestations. This is the first such analysis reported. By identifying mutation-induced altered transcriptomes in patient-derived retinal cells that predispose rods to cell stress, this study provides the first clues into molecular mechanisms underlying USH1B-RP.

### Limitations of USH1B patient-derived 3D retinal organoid model

Recent studies comparing retinal organoids with human retina suggested *in vitro* differentiation recapitulates the rate of retina development *in vivo* (Cowan et al., 2020). Consistently, bulk and scRNA-seq as well as mapping of the 11 USH genes to specific retinal cell types demonstrated the faithfulness of our organoid model as a surrogate for USH1B disease modeling. Despite these similarities, the level of *MYO7A* expression in 28 weeks control organoids was lower than that of the adult retina. This may be due to a reduction or absence of cellular activities involving MYO7A in the organoids. For example, the absence of juxtaposition of RPE to photoreceptors precludes OS phagocytosis. In addition, immature electrophysiological responses commonly reported in retinal organoids suggest attenuated phototransduction, possibly due to reduced protein trafficking and lack of the visual cycle (Hallam et al., 2018). Last, a lack of development of CP could abolish the need for USH1 protein linkage formation between OS and CP. The lack of retinal vasculature, blood supply and resident microglia in the organoid model also limits modeling of mutation-induced inflammatory responses, which may be important for understanding the notable identification of dysregulation of genes associated with neutrophil activation pathways in the USH1B rods and MC in this study (Table S5). In summary, although organoids provide valuable new insights into retinal disease pathology, further improvement on complexity and functionality of the model could improve representation of disease conditions *in vivo*.

### Retinal cell types expressing *MYO7A*

Previous studies of USH1B focused entirely on the role of MYO7A in rods and the RPE on the basis of the clinical phenotype and distribution pattern of MYO7A in animal models. Analysis of *MYO7A*^+^ cells in this study identified the majority of them as MCs, followed by BCs, rods, and cones. This raises the question of why, when *MYO7A* is mutated, rods are the first cell type to degenerate, which is typical of all RP clinically. Significantly, our scRNA-seq data showed heightened defensive responses against stress exclusively by rods, modeling their clinical susceptibility.

In addition, we found activation of apoptotic signaling pathways in USH1B patient MCs. MC death has not been widely studied or considered a primary event in RP. However, OCT examination of USH1B patient retina identified increased OLM visibility likely attributed to MC response to nearby photoreceptor cell stress or death (Jacobson et al., 2009). The same study also attributes OPL and INL thickening in USH1B patient retina to MC hypertrophy or hyperreactivity. Interrogating the role of MYO7A in the various cell types in which it is expressed would be important to further understand USH1B disease mechanism. To date we have not been able to confirm MYO7A protein localization in MCs using IHC with three different antibodies (Table S2) (data not shown), suggesting post-transcriptional control mechanisms and/or low or transient protein.

### Dysregulated GO terms over time: Potential disease markers of mutation-induced state

Over time in culture, USH1B organoids displayed increased numbers of DEGs associated with the ubiquitin-proteosome system (UPS), which is known to function in response to stress. Notably we found TOPORS and UBR1 dysregulated in the USH1B organoids (GO:0010498; Figure 4F; Table S4); mutation of the former is a cause of autosomal dominant RP (Chakarova et al., 2007), and the latter promotes rhodopsin protein degradation in the OS via the UPS during retinal inflammation (Ozawa et al., 2008). Altered protein trafficking of rhodopsin with a P23H mutation overwhelms the proteasome machinery due to UPS inefficiency in degrading the variant, leading to malfunction in various cellular pathways (Illing et al., 2002). The 28 weeks DEGs were also enriched for RNA splicing (GO:0008380). More than 25% of retinal disease-causing mutations alter splicing patterns (Bacchi et al., 2014). Mutation of the ubiquitously expressed pre-mRNA processing factors (PRPFs) accounts for 15% of autosomal-dominant RP (Wang et al., 2019). Several members of PRPFs (*PRPF18*, *PRPF31* and *PRPF39*) were dysregulated in the USH1B organoids (Table S4). A recent study showed that USH1G (SANS) interacts with PRPFs to facilitate the transport of small nuclear ribonucleoprotein during pre-RNA splicing (Yildirim et al., 2021). *PRPF31* mutation causes autosomal-dominant RP (RP11) and adversely affects primary cilia formation in photoreceptors and RPE function (Buskin et al., 2018). Last, with regard to cellular transport, *KIF3A* and *KIF3B* (a RP gene) along with other members of the kinesin family (GO:0048193) as well as components of the intraflagellar transport system (GO:0030705), including *IFT20* were dysregulated in USH1B organoids.

### Antioxidant enzymes differentially expressed between the control and patient retinal cells

Single-cell analysis of USH1B patient cells showed elevation of defensive cellular responses exclusively in rods. One interpretation of these findings is that patient cells were more sensitive to cellular stress compared with controls. Antioxidant enzymes including members from the peroxiredoxins (PRDXs) and superoxide dismutase (SOD) families were upregulated in rods. PRDX isoforms have previously been reported in different retinal cell types and organelles (Chidlow et al., 2016) and implicated in neurodegenerative and retinal diseases (Zheng et al., 2012; Kubo et al., 2009; Kim et al., 2001). However, PRDXs are not known to be expressed in rods under normal physiological conditions, and the absence of H_2_O_2_ scavenging PRDXs may contribute to susceptibility of rods to degeneration. *Sod1*^*−/−*^ mouse retina showed mitochondrial degeneration in both INL and ONL and AMD-like phenotypes (Hashizume et al., 2008). *BNIP3* was another notable dysregulated defensive gene identified as part of the cellular response to oxidative stress in *NRL*^*+*^ rods in patient organoids (GO:0034599; Figure 6C). *BNIP3* encodes a pro-apoptotic factor that interacts with anti-apoptotic proteins. A HIF-1α/BNIP3 pathway is neuroprotective against inner retinal neurodegeneration (Kunimi et al., 2021), whereas overexpression of BNIP3/FUNDC1 signaling reversed cell stress and death caused by HIF-1α inhibition in an hypoxic retinal model (Sun et al., 2021).

### Cellular responses in rods against stress: One-hit model

A cumulative damage model is often used to describe cell death in neurodegenerative disorders, whereby gene mutation causes the accumulation of secondary damage in cells, increasing the likelihood of a cell dying over time. In contrast, the one-hit model described by Clarke et al. (2000) proposes that the risk of a cell’s dying remains constant. Cells harboring gene mutations acquire a nearly normal homeostatic state called the mutant steady state (MSS), whereby a critical, but not lethal, alteration of gene activity that regulates pre-death reactions is present. Initiation of cell death only occurs when rare and random catastrophic events cause a surge of pre-death molecules to pass a critical cell-death threshold.

The altered transcriptomes of USH1B cells are consistent with an MSS being established in organoids. The enrichment of cell death-related pathways and cellular stress pathways (pre-death markers) may reflect the deployment of stress-mitigating enzymes to maintain the MSS and suppress cells from reaching the cell-death threshold. We speculate that the ample supply of nutrients *in vitro* and minimal exposure to light damage may limit the occurrence of catastrophic events leading to significant levels of cell death. The introduction of various stressors that an individual would normally experience in life, including exposure to natural light, free radicals, and reactive oxygen species, into the organoid system may better mimic the aging USH1B degenerative disease state.

## Experimental procedures

Additional details are provided in the supplemental information.

### iPSC generation

The study was approved by the National Research Ethics Committee London-Dulwich (11/LO/1243). Fibroblasts from USH1B patients and controls were reprogrammed to iPSC using Sendai virus. Details of mutations are given in Figure S1.

### Retinal organoid differentiation and analysis

iPSCs were grown to 90% confluency before switching to embryoid body (EB) medium on day 0, neural induction medium (NIM) on day 2, followed by retinal differentiation medium (RDM) containing various supplements (RDM1–3) for retinal maturation and long-term culture of organoids. EB, NIM, RDM, and RDM1–3 are described in Table S1. Organoids were analyzed using RT-PCR, transmission electron microscopy (TEM), and western blot as described in supplemental information.

### Immunohistochemistry

Retinal organoids were fixed with 4% paraformaldehyde (PFA) solution and cryosections of frozen organoids analyzed using immunohistochemistry. Primary and secondary antibodies are listed in the Table S2.

### Bulk RNA sequencing

RNA (100 ng) from organoids was processed using the KAPA mRNA Hyper−Prep Kit (KK8580; Roche) and libraries sequenced on a S1 flow cell on a NovaSeq 6000 system (Illumina). FASTQ files from 20 million reads per sample were aligned to the human genome UCSC hg38 using RNA-STAR 2.5.2b. Differential expression analysis was performed using limma-voom with cut-off of adjusted p value < 0.05 for statistical significance. Package clusterProfiler (version 3.0.4) was used for GO enrichment analysis.

### Single-cell RNA sequencing

Single-cell RNA libraries were generated using the Chromium Single Cell 3ʹ Reagent Kits version 3 (10X Genomics) and analyzed on an Illumina sequencing platform with 10X Genomics Cell Ranger software to generate digital gene expression matrices. Data from 39,934 cells were analyzed using Seurat. The “FindMarkers” function was used to identify differentially expressed genes between control and patient cells globally or in a cell/cluster-specific manner.

### Statistical analysis

Data were analyzed and graphs created using GraphPad Prism (version 9.2.0) or Microsoft Excel (version 2110). One-way ANOVA with Dunnett correction was performed on data from at least three independent experiments, represented as mean ± SD, unless stated otherwise. A p value < 0.05 or an adjusted p value < 0.05 was considered to indicate statistical significance. Graphical abstract and schematics were created in BioRender.com.

### Data and code availability

RNA-seq data have been deposited in ArrayExpress under accession numbers ArrayExpress: E_MTAB-11405 (Bulk RNA-seq) and ArrayExpress: E-MTAB-11990 (single-cell RNA-seq).

## Author contributions

Conceptualisation, J.C.S., V.D.F., and Y.C.L.; Methodology, Y.C.L. and J.C.S.; Investigation, Y.C.L. and V.D.F.; Collection of Patient Samples, M.B.G.; Data Analysis and Interpretation, Y.C.L., A.P., V.D.F., and J.C.S.; Single-Cell Library Preparations, A.P.; Resources, H.P., A.P., M.B.G., and J.C.S.; Validation, Y.C.L.; Writing, Y.C.L. and J.C.S.; Final Approval of Manuscript, J.C.S.; Supervision, J.C.S.; Funding, J.C.S.

## Supplemental information

Document S1. Figures S1–S6 and Tables S1–S3

Table S4. Bulk RNA-seq analysis, related to Figures 3 and 4

Table S5. Single-cell RNA-seq analysis, related to Figures 5 and 6

Document S2. Article plus supplemental information
