# Supplementary material for: Molecular pathology of Usher 1B patient-derived retinal organoids at single cell resolution
Source: Stem Cell Reports. 2022 Oct 13;17(11):2421–37. doi: 10.1016/j.stemcr.2022.09.006 (PMC9669639; doi:10.1016/j.stemcr.2022.09.006)
Supplement: Document S1. Figures S1–S6 and Tables S1–S3 [file mmc1.pdf]

Stem Cell Reports, Volume *17*

Supplemental Information

Molecular pathology of Usher 1B patient-derived retinal organoids at single cell resolution

Yeh Chwan Leong, Valentina Di Foggia, Hema Pramod, Maria Bitner-Glindzicz, Aara Patel, and Jane C. Sowden

A

Patient	Mutation			
	Allele 1		Allele 2	
	Gene	Protein	Gene	Protein
USH1B.1	c.133-2A>G	Acceptor splice site mutation, intron3 (IVS3-2A-G)	c.1996C>T	p.Arg666*
USH1B.2	c.223G>C	p.Asp75His	c.6070C>T	p.Arg2024*
USH1B.3	c.223G>C	p.Asp75His	c.6070C>T	p.Arg2024*

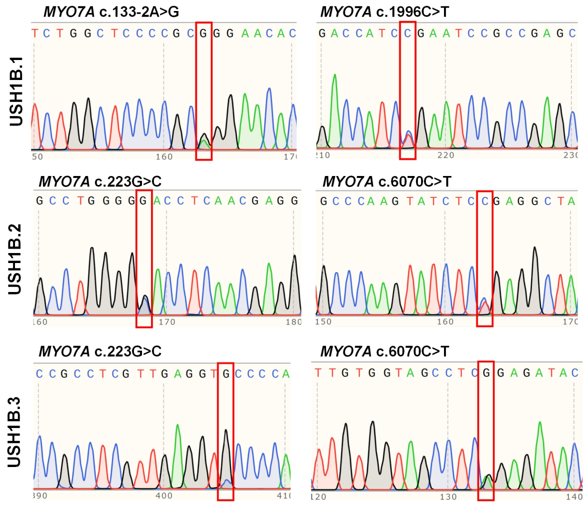

B

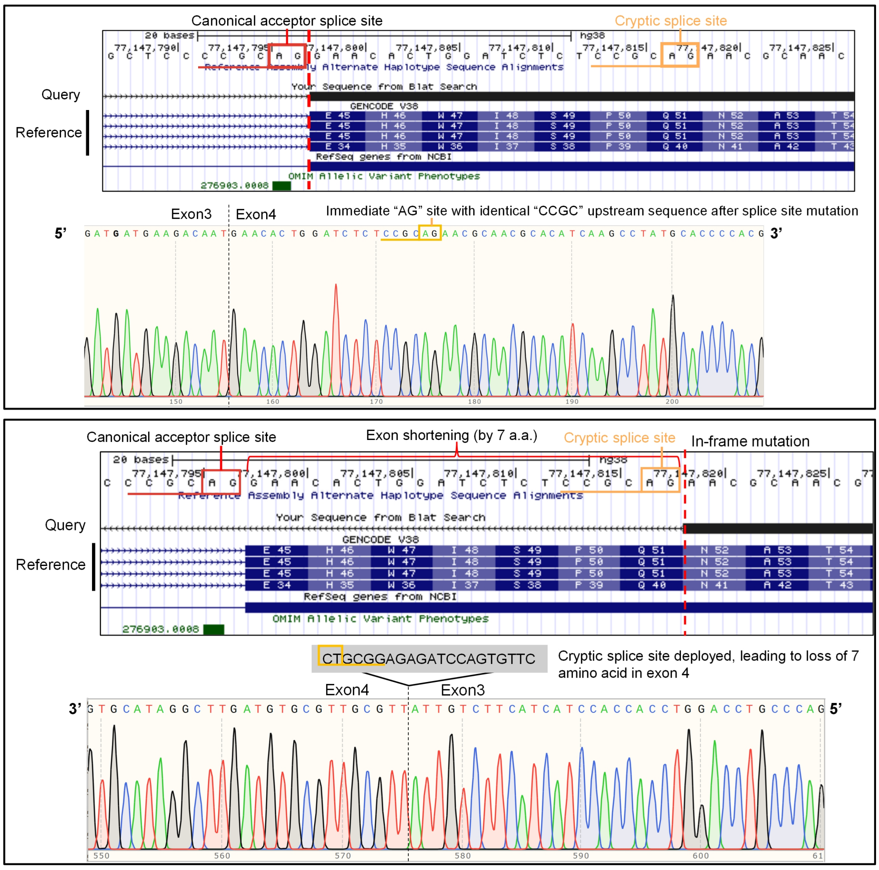

Figure S1 Confirmation of patient *MYO7A* mutations in patient-derived iPSC. Related to experimental procedure. A) (Top) Details of mutations harboured by three USH1B patients (Lenassi et al., 2014). (bottom) Sanger Sequencing confirmed patient MYO7A mutations in patient iPSCs. **B)** Sanger sequencing of cDNA from USH1B.1 patient showed deletion of 7 amino acid as a result of an acceptor splice site mutation, c.133-2A>G. (Top) sequence of a wild type allele. (bottom) sequence of allele with c.133-2A>G mutation.

A

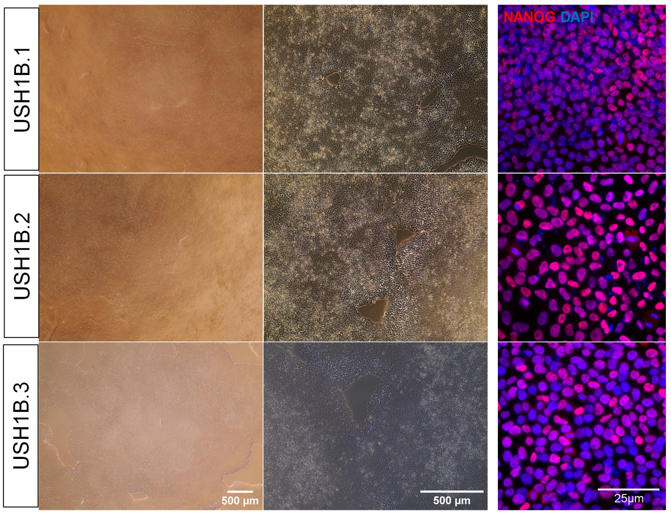

B

iPSC line used in this study	iPSC line name	SNP array (passage number)	Passages used in this study
Control.1	DF19-9-11T.H	43	40-46
Control.2	iPS(IMR90)-4	41	38-42
Control.3	GOS101	24	23-29
Control.4	N7	30	21-34
USH1B.1	Youd6	23	18-24
USH1B.2	N35.1	31	28-31
USH1B.3	GOS102	29	20-26

C

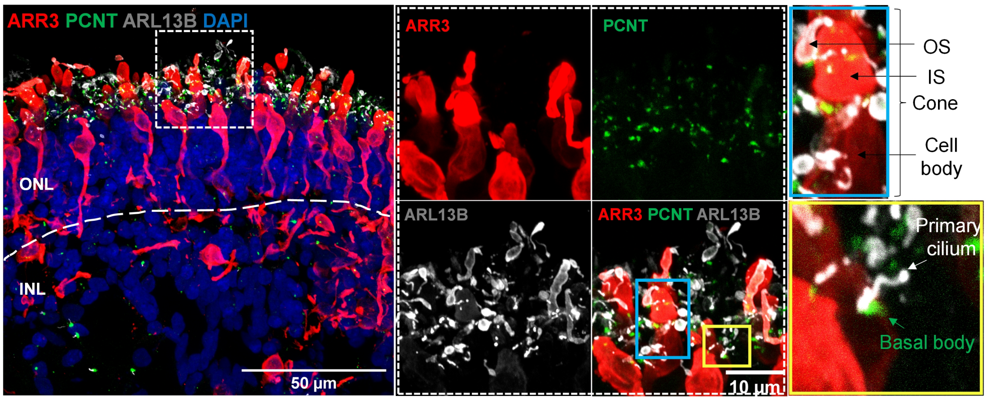

D

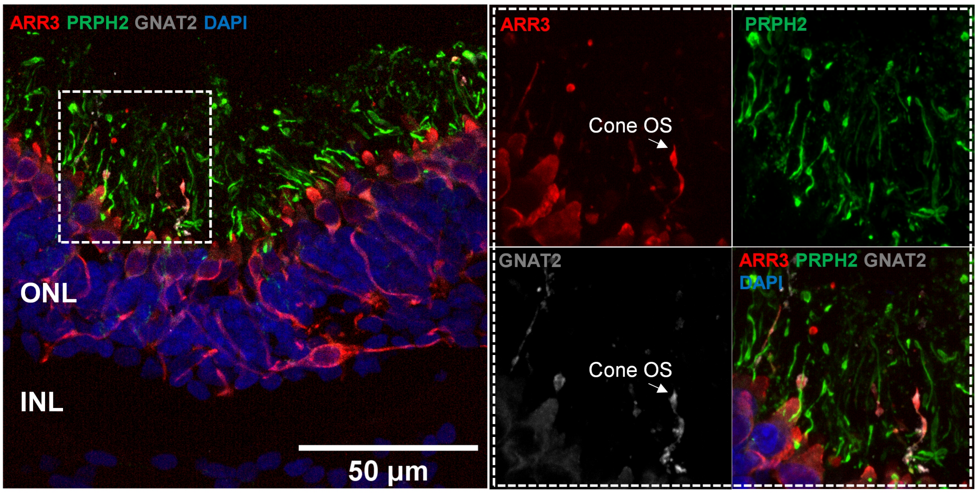

Figure S2 Characterisation of control- and USH1B patient-derived iPSCs and their genome integrity; Subcellular features indicative of photoreceptor maturation. Related to Figure 1 and Figure S1. A) Brightfield images show iPSCs maintained compact cellular morphology with well-defined edges, high nucleus-to-cytoplasm ratio (left and middle columns) and expressed pluripotency marker, NANOG (right column). **B)** Table shows (from first column) nomenclature of all iPSCs used in this study, their original names and source facility where iPSC were generated, passage number when Single Nucleotide Polymorphism (SNP) array analysis was performed, and all passages used in this study. SNP array data available upon request. **C)** Connecting cilium (expressing cilia marker, ARL13B; basal body marker, PCN) was present in photoreceptors. ARL13B also marked the photoreceptor outer segment. ARR3, cone marker. Observed in >3 organoids per iPSC line. **D)** Immunostaining of 28wks retinal organoids for outer segment markers. PRPH2, rod outer segment; GNAT2, cone outer segment. Observed in >3 organoids per iPSC line.

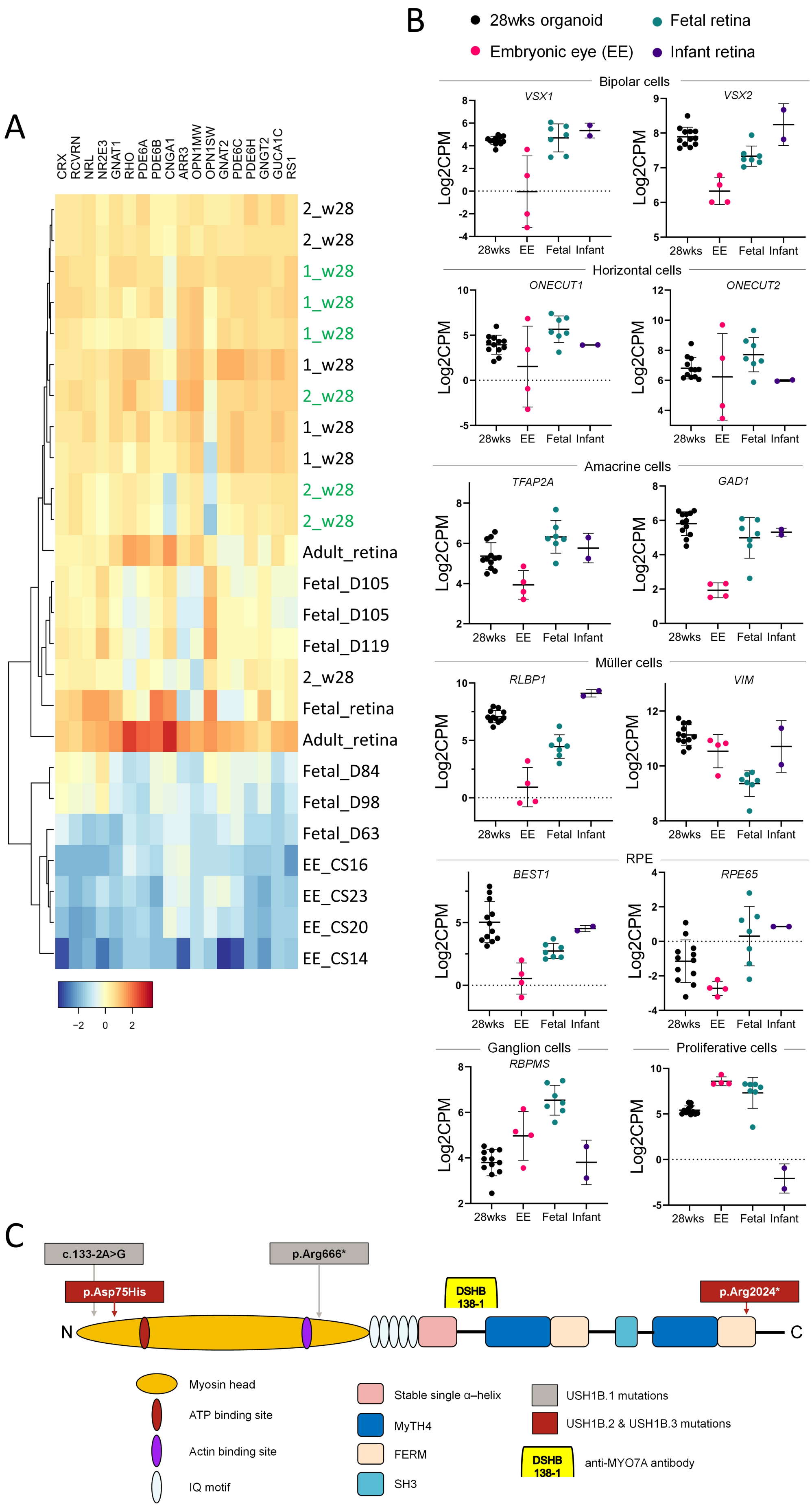

A
CRX
RCVRN
NRL
NR2E3
GNAT1
RHO
PDE6A
PDE6B
CNGA1
ARR3
OPN1MW
OPN1SW
GNAT2
PDE6C
PDE6H
GNGT2
GUCA1C
RS1
2_w28
2_w28
1_w28
1_w28
1_w28
1_w28
2_w28
1_w28
1_w28
2_w28
2_w28
Adult_retina
Fetal_D105
Fetal_D105
Fetal_D119
2_w28
Fetal_retina
Adult_retina
Fetal_D84
Fetal_D98
Fetal_D63
EE_CS16
EE_CS23
EE_CS20
EE_CS14
−2
0
2
B
28wks organoid
Fetal retina
Embryonic eye (EE)
Infant retina
Bipolar cells
VSX1
VSX2
Horizontal cells
ONECUT1
ONECUT2
Amacrine cells
TFAP2A
GAD1
Müller cells
RLBP1
VIM
RPE
BEST1
RPE65
Ganglion cells
RBPMS
Proliferative cells
Log2CPM
28wks
EE
Fetal
Infant
C
c.133-2A>G
p.Arg666*
p.Asp75His
DSHB 138-1
p.Arg2024*
N
C
Myosin head
ATP binding site
Actin binding site
IQ motif
Stable single α–helix
MyTH4
FERM
SH3
USH1B.1 mutations
USH1B.2 & USH1B.3 mutations
anti-MYO7A antibody

Figure S3 28wks retinal organoids were similar to fetal and adult retina based on expression of markers of different retinal cell types. Related to Figure 3 and experimental procedure. A) Heatmap shows expression of photoreceptor markers comparing data from human developing eye (GEO accession: GSE98370, Mellough et al, 2019; encompassing three developmental stages: human embryonic eye (CS14–16), fetal (9 – 17wks), infant (74pcw) and RNA-seq data from 28wks USH1B and control organoids (this study). **B)** Expression of markers of other retinal cell types in human developing eye with USH1B and control organoids (this study). **C)** Schematic of MYO7A shows anti-MYO7A antibody (DSHB, 138-1) binding site used in Western blot shown in Figure 3I-I'. MYO7A contains an N-terminal motor domain, where ATP- and actin-binding sites reside and is connected to a neck region composed of 5 isoleucine-glutamine (IQ) motifs followed by a complex tail region, which binds to cargos or other proteins to form a complex. The tail region begins with a stable single α–helix (SAH) followed by two MyTH4 (myosin tail homology 4)-FERM (four-point1, ezrin, radixin and myosin) regions (MF1 and MF2) sandwiching a SRC homology 3 (SH3) domain (Kabahuma et al., 2021, Heissler and Manstein, 2012).

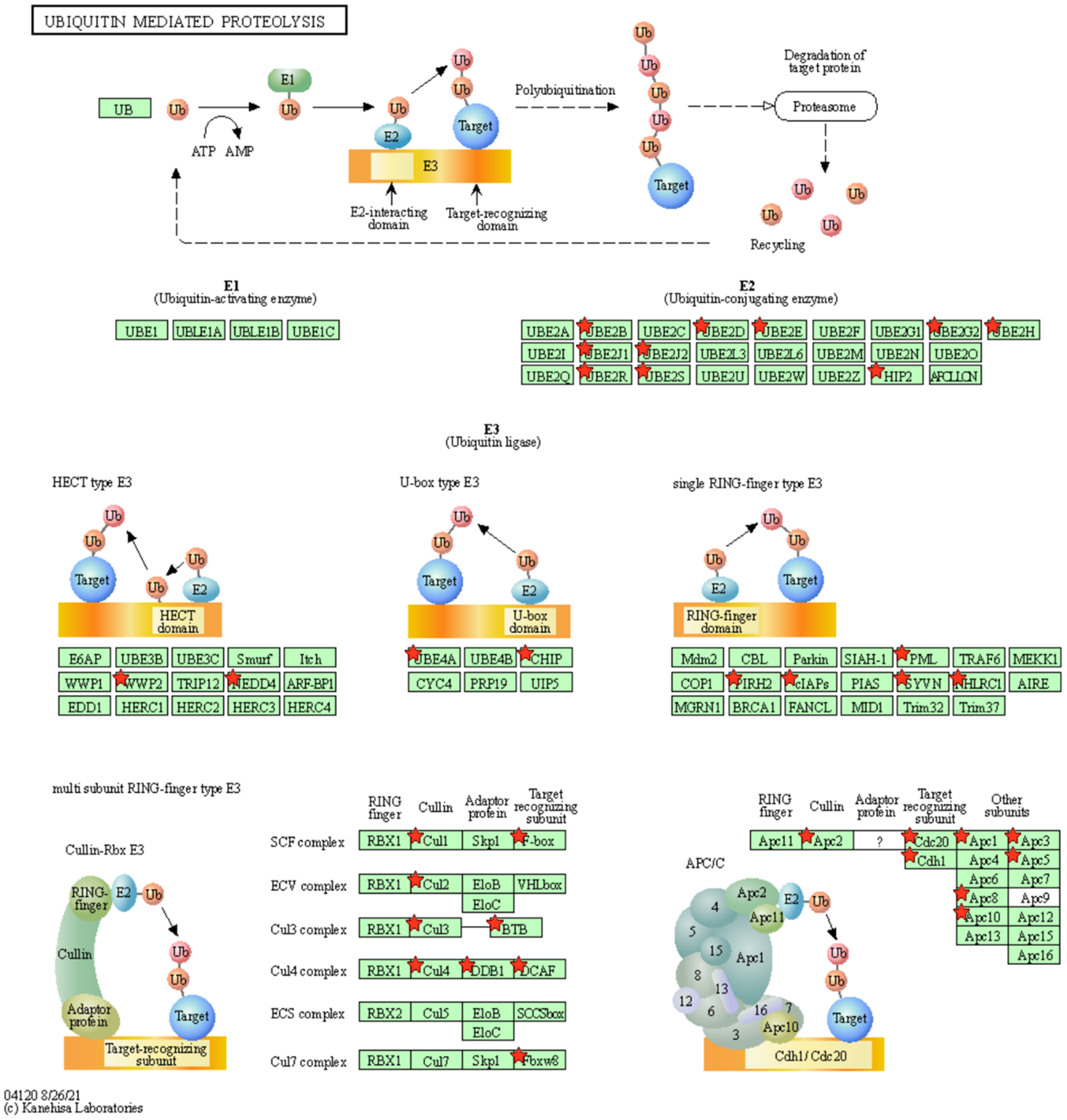

Figure S4 KEGG pathway (04121) ubiquitin mediated proteolysis. Related to Figure 4F. Highlighted in red are the 40 genes in the KEGG ubiquitin mediated proteolysis pathway that were dysregulated in the 28wks USH1B organoids (Benjamini p-value 4.4×10^{-39}; generated using david-d.ncifcrf.gov)

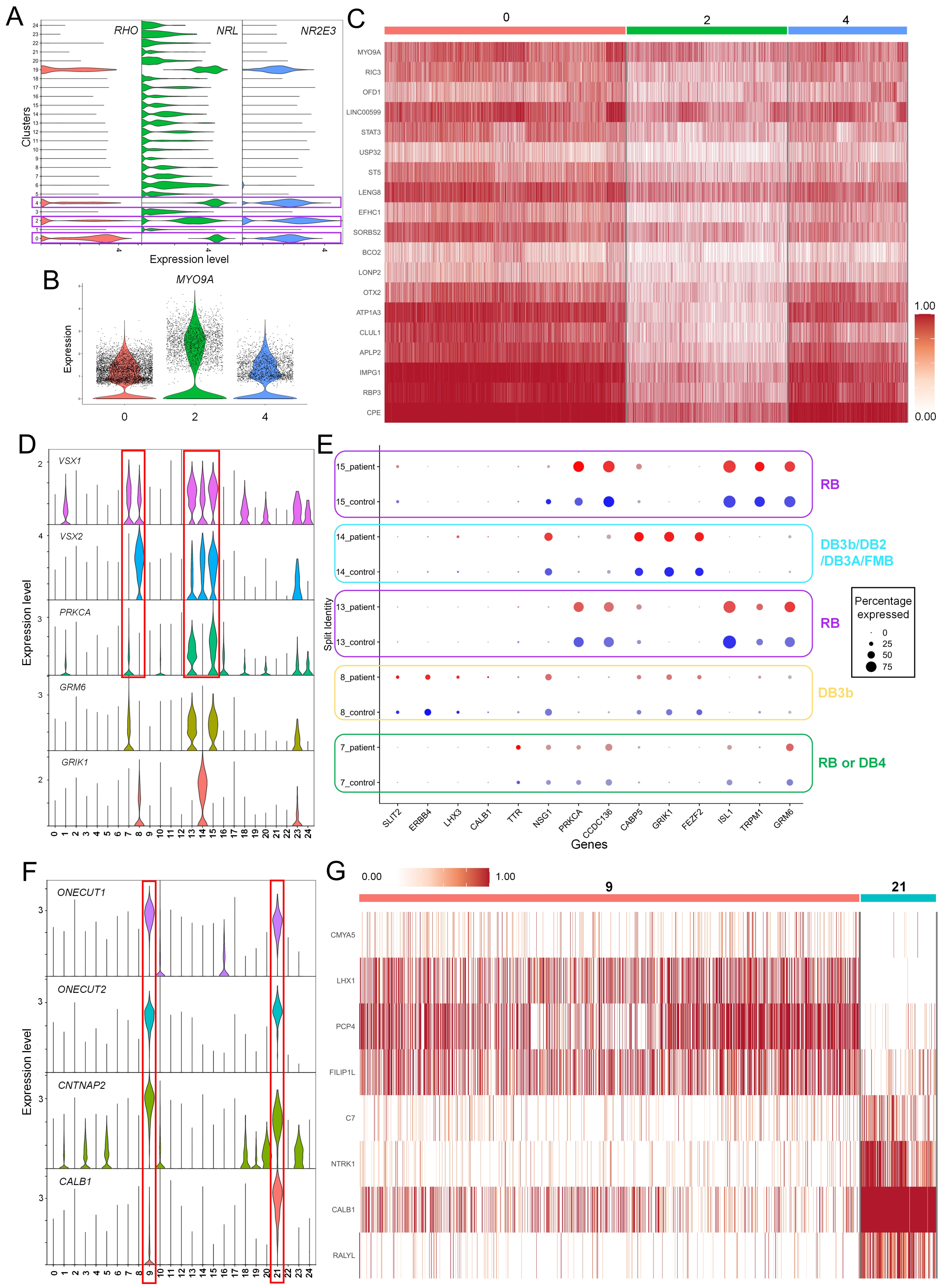

A
RHO
NRL
NR2E3
Clusters
Expression level
B
MYO9A
Expression
0
2
4
C
0
2
4
MYO9A
RIC3
OFD1
LINC00599
STAT3
USP32
ST5
LENG8
EFHC1
SORBS2
BCO2
LONP2
OTX2
ATP1A3
CLUL1
APLP2
IMPG1
RBP3
CPE
1.00
0.00
D
VSX1
VSX2
PRKCA
GRM6
GRIK1
Expression level
E
15_patient
15_control
14_patient
14_control
13_patient
13_control
8_patient
8_control
7_patient
7_control
Split Identity
RB
DB3b/DB2
/DB3A/FMB
RB
DB3b
RB or DB4
Percentage expressed
0
25
50
75
SLIT2
ERBB4
LHX3
CALB1
TTR
NSG1
PRKCA
CCDC136
CABP5
GRIK1
FEZF2
ISL1
TRPM1
GRM6
Genes
F
ONECUT1
ONECUT2
CNTNAP2
CALB1
Expression level
G
0.00
1.00
9
21
CMYA5
LHX1
PCP4
FILIP1L
C7
NTRK1
CALB1
RALYL

Figure S5 The presence of retinal cell subtypes in 35wks retinal organoids. Related to Figure 5. A) Rod photoreceptor clusters (0, 2 and 6) were indicated by *RHO*, *NRL* and *NR2E3* expression. **B)** Two rod subtypes described by Yi *et al.* (2021) that can be differentiated by *MYO9A* expression. Cluster 2 expressed high level of *MYO9A* compared to cluster 0 and 4. **C)** Heatmap shows the expression, by cluster 0, 2 and 6, of the most distinctive genes comparing human *MYO9A*$^{+}$ and *MYO9A*$^{-}$ rods identified by Yi *et al.* (2021). **D)** *GRIK1*, *GRM6*, *VSX1*, *VSX2* and *PRKCA* expression identified bipolar cell clusters: 7, 8, 13, 14 and 15; *GRM6* and *GRIK1* expression were indicative of ON- and OFF-bipolar cells, respectively. **E)** More bipolar cell subtypes can be differentiated by expression of markers described by Yi *et al.* (2021). **F)** Horizontal cell clusters were indicated by the expression of *CNTNAP2*, *CALB1*, *ONECUT1* and *ONECUT2:* 9 and 21. **G)** Heatmap shows the expression, by cluster 9 and 21, of the most distinctive genes comparing human H1 and H2 horizontal cells identified by Yi et al. (2021).

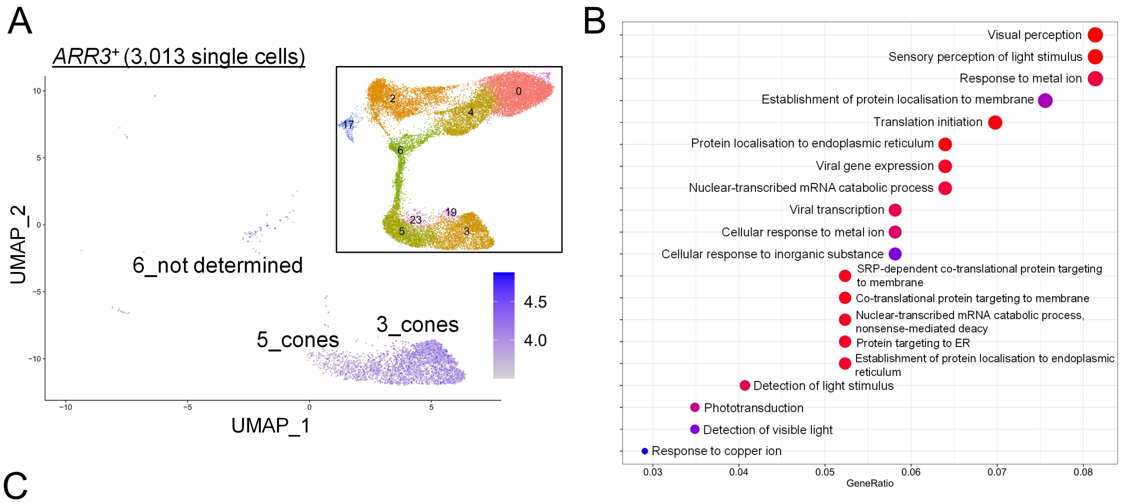

C

ID	Description	qvalue	geneID
GO:0007601	Visual perception	<0.001	CABP2/CNGB3/CPLX4/EYS/GRK7/GUCA1B/NRL/OPN1MW/PDE6G/RBP3/RDH10/RP1L1/RPGRIP1/RS1
GO:0050953	Sensory perception of light stimulus	<0.001	CABP2/CNGB3/CPLX4/EYS/GRK7/GUCA1B/NRL/OPN1MW/PDE6G/RBP3/RDH10/RP1L1/RPGRIP1/RS1
GO:0070972	Protein localization to endoplasmic reticulum	<0.001	ANK2/RPL13A/RPL17/RPL37/RPL41/RPS10/RPS17/RPS21/RPS26/RPS4X/TAPT1
GO:0006413	Translational initiation	<0.001	CSDE1/EIF1AX/EIF2S3/RPL13A/RPL17/RPL37/RPL41/RPS10/RPS17/RPS21/RPS26/RPS4X
GO:0006614	SRP-dependent co-translational protein targeting to membrane	<0.001	RPL13A/RPL17/RPL37/RPL41/RPS10/RPS17/RPS21/RPS26/RPS4X
GO:0006613	Co-translational protein targeting to membrane	<0.001	RPL13A/RPL17/RPL37/RPL41/RPS10/RPS17/RPS21/RPS26/RPS4X
GO:0000184	Nuclear-transcribed mRNA catabolic process, nonsense-mediated decay	0.001	RPL13A/RPL17/RPL37/RPL41/RPS10/RPS17/RPS21/RPS26/RPS4X
GO:0045047	Protein targeting to ER	0.001	RPL13A/RPL17/RPL37/RPL41/RPS10/RPS17/RPS21/RPS26/RPS4X
GO:0072599	Establishment of protein localization to endoplasmic reticulum	0.001	RPL13A/RPL17/RPL37/RPL41/RPS10/RPS17/RPS21/RPS26/RPS4X
GO:0019080	viral gene expression	0.001	CSDE1/JUN/RPL13A/RPL17/RPL37/RPL41/RPS10/RPS17/RPS21/RPS26/RPS4X

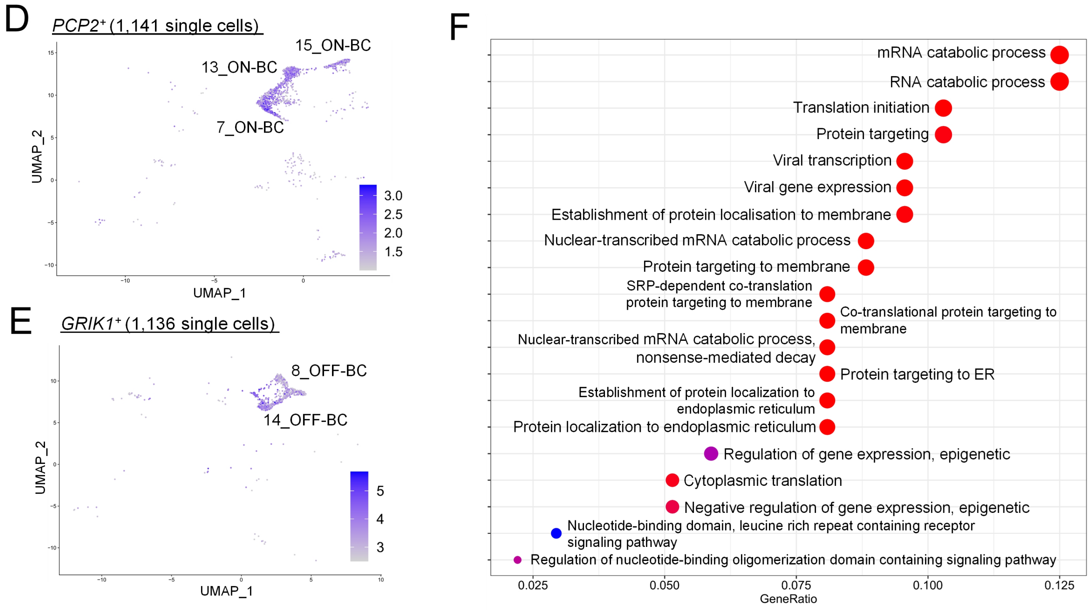

Figure S6 Cell type specific differential expression analysis of single-cell RNA-seq. Related to Figure 6. A) A total of 3,013 cone photoreceptors were isolated based on cone arrestin (*ARR3*) expression. **B)** Top 20 significantly enriched biological processes GO terms by analysis of DEGs between control and USH1B *ARR3*+ single cells. Ranked by gene ratio and q-value. **C)** Details of top 10 significantly enriched biological processes. Ranked by q-value. **D)** A total of 1,136 OFF-BC isolated based on *GRIK1* expression. **E)** A total of 1,141 ON-BC isolated based on *PCP2* expression. **F)** Top 20 significantly enriched biological processes GO terms by analysis of DEGs between control and USH1B *GRIK1*+/*PCP2*+ single cells. Ranked by gene ratio and q-value.

Table S1: Retinal differentiation media. Related to experimental procedures and Figure 1.

Reagent	Volume (mL)	Concentration	Catalogue number
**Embryoid body medium (500 mL)**			
DMEM/F12 (with Glutamax)	up to 500	-	Gibco 10565-018
KOSR	100	20%	Gibco 10828-028
MEM non-EAAs (100X)	5	1x	Gibco 11140-050
β-ME (50 mM)	1	0.1 mM	Gibco 31350-010
**Neural Induction Medium (NIM) (500 mL)**			
Advanced DMEM/F12	up to 500	-	Gibco 12634-010
MEM non-EAAs	5	1x	Gibco 11140-050
N2 supplement	5	1x	Gibco 17502-048
GlutaMax	5	1x	Gibco 35050-038
**Retinal Differentiation Medium (RDM) (500 mL)**			
DMEM (with L-glutamine)	up to 500	-	Gibco 41965-039
F12	150	-	Gibco 21765-029
B27 supplement	10	1x	Gibco 12587-010
**RDM1 (250 mL)**			
RDM	Up to 250	-	
FBS	25	10 %	LifeTech, 10270106
GlutaMax	2.5	1x	Gibco, 35050-038
Taurine	250 µL	100 µM	Sigma, T8691
**RDM2 (50mL)**			
RDM1	50	-	Gibco 10565-018
Retinoic Acid	-	1 µM	Sigma R2625
**RDM3 (50mL)**			
DMEM/F12 with Glutamax	50	-	Gibco 10565-018
Retinoic Acid	-	0.5 µM	Sigma R2625
N2 supplement	500 µL	1x	Gibco 17502-048

Table S2: List of PCR primer and antibodies used for immunohistochemistry and Western blot, related to Figure 1-3 and experimental procedures.

Gene name	Forward primer (5'-3')	Reverse primer (5'-3')		
GAPDH	CACCATCTTCCAGGAGCGAG	GACTCCACGACGTACTCAGC		
RCVRN	CCTCTACGACGTGGACGGTAA	GTGTTTTCATCGTCTGGAAGGA		
NRL	GGGCTGAGTCCTGAAGAGG	TTTAGCTCCCGCACAGACAT		
CRX	GTCCAGGGTTCAGGTTTGGT	GTGCCCGCCTTCCTCTTG		
ARR3	AGTCCTACAGGAGCGACTACT	GGCAGGTTGGTCACCATCTG		
RHO	ACAGGATGCAATTTGGAGGGC	GCTCATGGGCTTACACACCA		
**Antibodies**	**Full protein names**	**Company (catalogue number)**	**Host**	**Dilution**
**Immunohistochemistry**				
CRX	Cone-rod homeobox protein	Abnova (H00001406-M02)	Mouse	1:800
VSX2	Visual system homeobox 2	Merck Millipore (AB9016)	Sheep	1:200
RCVRN	Recoverin	Merck Millipore (A85585)	Rabbit	1:800
PAX6	Paired box protein Pax-6	Biolegend (901301)	Rabbit	1:200
ARR3	Cone arrestin	Novus (NBP-37003)	Goat	1:100
NRL	Neural retina-specific leucine zipper protein	R&D Systems (AF2945)	Goat	1:100
NR2E3	Photoreceptor-specific nuclear receptor	Persus Proteomics (PP-H7223-00)	Mouse	1:500
S-opsin	Short-wave-sensitive opsin 1	Merck Millipore (AB5407)	Rabbit	1:200
L/M-opsin	Medium/Long-wave-sensitive opsin 1	Merck Millipore (AB5405)	Rabbit	1:200
RHO	Rhodopsin	Sigma (O4886)	Mouse	1:800
PRKCA	Protein kinase C alpha type	GeneTex (GTX11723)	Mouse	1:20
ARL13B	ADP-ribosylation factor-like protein 13B	Proteintech (17711-1-AP)	Rabbit	1:500
GNAT2	Guanine nucleotide-binding protein G(t) subunit alpha-2	Santa Cruz Biotechnology (SC390)	Rabbit	1:200
PRPH2	Peripherin-2	Merck Millipore (MABN293)	Mouse	1:200
BRN3A	POU domain, class 4, transcription factor 1	Merck Millipore (MAB1585)	Mouse	1:100
SPY	Synaptophysin	Abcam (AB8049)	Mouse	1:100
TFAP2A	Transcription factor AP-2-alpha	DSHB 3B5	Mouse	1:500
PCNT	Pericentrin	Abcam (AB28144)	Mouse	1:500
Cleaved-CASP3	Cleaved-caspase3	Cell Signalling (9661S)	Rabbit	1:200
γH2AX	Gamma histone H2AX	Abcam (AB2893)	Rabbit	1:400
NQO1	NAD(P)H dehydrogenase [quinone] 1	Abcam (AB2346)	Goat	1:100
BiP/GRP78	Endoplasmic reticulum chaperone BiP	Abcam (AB21685)	Rabbit	1:300
8-OHdG	8-Hydroxy-2'-deoxyguanosine	Abcam (AB48508)	Mouse	1:200
GFAP	Glial fibrillary acidic protein	Abcam (AB5804)	Rabbit	1:200
MYO7A	Unconventional myosin-VIIa	Abcam (Ab3481)	Rabbit	1:200
MYO7A	Unconventional myosin-VIIa	DSHB (138-1)	Mouse	1:200
MYO7A	Unconventional myosin-VIIa	Proteus (256790)	Rabbit	1:200
**Western blot**				
GFAP	Glial fibrillary acidic protein	Abcam (AB5804)	Rabbit	1:1000
ACTB	Actin, cytoplasmic 1	Sigma (A2228)	Mouse	1:5000
MYO7A	Unconventional myosin-VIIa	Abcam (Ab3481)	Rabbit	1:500
MYO7A	Unconventional myosin-VIIa	DSHB (138-1)	Mouse	1:500
MYO7A	Unconventional myosin-VIIa	Proteus (256790)	Rabbit	1:2000

Table S3: Publicly available human embryonic eye and retina RNA-seq data (GEO: GSE98370) (Mellough et al., 2019). Related to Figure 3.

Sample accession	Sample title	Timepoint
SAMN06854090	12301 Embryonic CS14 Eye RNA	CS14
SAMN06854087	12556 Embryonic CS16 Eye RNA	CS16
SAMN06854084	12527 Embryonic CS20 Eye RNA	CS20
SAMN06854081	1901 Embryonic CS23 Eye RNA	CS23
SAMN06854080	13149 Foetal 1 Retina RNA	63days
SAMN06854077	1940 Foetal 4 Retina RNA	84 days
SAMN06854074	12552 Foetal 6 Retina RNA	98 days
SAMN06854071	12773 Foetal 7 Retina RNA	105 days
SAMN06854070	12xxx1 Foetal 7 Retina RNA	105 days
SAMN06854064	13172 Foetal 9 Retina RNA	119 days
SAMN06854091	191115 Adult Retina RNA	74pcw
SAMN06854092	010813 Adult Retina RNA	77pcw

Table S4: Bulk RNA-seq analysis. Related to Figure 3 and 4.

Table S5: Single cell RNA-seq analysis. Related to Figure 5 and 6.

Supplemental Experimental Procedures

Generation and maintenance of iPSCs

The study was approved by the National Research Ethics Committee London-Dulwich (11/LO/1243). Skin biopsies were collected from consenting patients or guardians of patients with a clinical genetic diagnosis of USH1B who attended clinics at Great Ormond Street Hospital (GOSH) or unaffected individuals as controls. Fibroblasts from patient 1 (USH1B.1), patient 2 (USH1B.2) and patient 3 (USH1B.3) were reprogrammed to iPSC using Sendai virus by the HipSci (the human Induced Pluripotent Stem Cell Initiative funded by the Wellcome Trust and MRC, www.hipsci.org), NIHR Cambridge Biomedical Research Centre human iPSCs core facility and NIHR GOSH Biomedical Research Centre iPSC facility, respectively. Control.3 iPSC was generated through the same route as USH1B.3, Control.4 iPSC was generated through the same route as USH1B.2, and control.1 (DF19-9-11T.H) and control.2 (iPS(IMR90)-4) were purchased from WiCell. All iPSCs were cultured on Vitronectin XF (Stem Cell Technologies, 100-0763) with Essential 8 medium (Gibco, A1517001). iPSC lines maintained a healthy morphology and growth rate, and expressed pluripotency markers OCT4, SOX2, TRA-1-60 and NANOG. *MYO7A* gene mutations were confirmed in patient iPSCs with Sanger sequencing. Details of mutations are given in Figure S1. iPSC cultures displayed a normal growth rate and morphology and maintained pluripotency marker expression. Single- nucleotide polymorphism (SNP) karyotype analysis of the iPSC lines and passage numbers used are given in Figure S2. SNP microarray analysis was performed using HumanCytoSNP-12 v2.1 BeadChip Kit from Illumina and Genomestudio 2.0 confirmed the edited line retained a normal digital karyotype (Data available upon request).

Immunofluorescence and microscopy

Retinal organoids were fixed with 4% PFA solution (Thermo Scientific, 28908) in phosphate buffer saline (PBS, Oxoid BR0014G) for 30 min at room temperature. Fixed organoids were washed thrice with PBS and incubated overnight in 30% sucrose (Sigma, S0389) solution in PBS (or in 7.5% gelatin/5% sucrose in PBS for connecting cilium analysis) before embedding in OCT (Fisher Scientific, 12678646) and stored at -80°C. Cryosections of frozen organoids were generated using a Leica cryostat and collected on microslide superfrost plus blue slides (VWR, 630-0950). For immunostaining, cryosections were left at room temperature for 30 min, washed once with PBS and incubated with blocking solution 10% donkey serum (Sigma, D9663), 1% (wt / vol) bovine serum albumin (Merck, A7906), 0.5% Triton X-100 (Merck, 112298) prepared in PBS for 1 h at room temperature. Primary and secondary antibodies were prepared in the same buffer containing 3% donkey serum, 1% BSA, 0.5% Triton X-100 in PBS. Sections were incubated at room temperature overnight in humidified chamber with primary antibodies, washed thrice with 0.1% TWEEN 20 (Sigma, P1379) in PBS, 15 min per wash, followed by incubation with secondary antibodies. After two washes with 0.1% TWEEN 20 in

PBS and one wash with PBS, sections were counterstained with DAPI, before mounting coverslips with ImmunoFluoroMount (GTX30928). Microscopy was performed using Zeiss LSM 710 confocal microscope. Image analysis was performed using FIJI (Schindelin et al., 2012). All primary and secondary antibodies are listed in the Table S2.

Quantification of organoid size and thickness of outer nuclear layer (ONL)
Organoid size was quantified from Fiji image analysis using 7wks organoid size as baseline because at 6wks, adherent organoids were micro-dissected and transferred to 3D suspension culture and at 7wks, medium was supplemented with fetal bovine serum, after which an increase in organoid size was observed. ONL thickness was quantified using Fiji with the areas measured guided by ARR3 (cone arrestin, cone photoreceptor) or RCVRN (recoverin, pan-photoreceptor) IHC plus DAPI counterstain in retinal cryosections.

RT-PCR and qRT-PCR
RNA extraction was performed using miRNeasy micro kit (Qiagen, 217084) and cDNA was synthesised using miScript II RT kit (Qiagen, 218160). RNA was quantified using Thermo Scientific™ NanoDrop™ One Microvolume UV-Vis Spectrophotometer (13-400-518). RT-PCR was performed using MyFi™ mix (Meridian Bioscience, BIO-25049) and PCR products were resolved in UltraPure™ agarose gel (Thermo Scientific, 16500100) stained by SYBR® Safe DNA Gel Stain (Thermo Scientific, S33102). For qRT-PCR, SYBR green PCR master mix (Life Technologies, 4309155) was used, and reactions were analysed with Applied Biosystem 7500 Real Time PCR system. Technical triplicates of independent samples were prepared. Results were normalised to GAPDH (internal control) and fold change was calculated using the $2^{-\Delta\Delta Ct}$ method. The sequences of primers used can be found in the Table S2.

Transmission electron microscope (TEM)
Retinal organoids were fixed with 3% EM grade glutaraldehyde in 0.1 M sodium cacodylate buffer and pH 7.4 5 mM CaCl2 for 15 min at room temperature and kept at 4°C before processing. Organoids were processed at the Institute of Neurology electron microscopy facility, UCL. TE micrographs were captured using a JEOL JEM-1400 120 kV transmission electron microscope.

Western blot analysis
Protein extraction was performed using 1x RIPA Buffer (Cell Signalling Technology, 9806) supplemented with 1x protease inhibitor cocktail (Sigma-Aldrich, P8340). Protein concentration was determined using a Pierce™ BCA Protein Assay Kit (Thermo Scientific, 23225) and analysed by Infinite 200 PRO NanoQuant microplate readers (Tecan). Western blotting was performed with 20 µg protein per sample. Polyvinylidene difluoride (PVDF) membrane (GE Healthcare, 10600023) was blocked with 5% non-fat milk (Sigma) overnight

at 4°C before staining with primary antibodies (prepared in 5% non-fat milk) overnight at 4°C on tube rollers. PVDF membrane was washed thrice with Tris-buffered saline (TBS)-tween 20 (TBST) (TBS and 0.5% Tween 20) solution (15 minutes per wash) before incubation with secondary antibodies (prepared in TBST) for 2 h at room temperature on shaker. Visualisation was performed with the Odyssey® CLx Infrared Imaging System (LI-COR Biosciences). Immunoblotting of MYO7A was performed using, with DSHB 138-1 anti-MYO7A antibody.

Bulk RNA-sequencing

Total RNA was extracted from retinal organoids and human neural retinal tissue using a miRNeasy micro kit (Qiagen, 217084). Human tissue was obtained from the Joint Medical Research Council UK (grant G0700089)/Wellcome Trust (grant GR082557) Human Developmental Biology Resource (http://www.hdbr.org/) and human adult eyes from Moorfields Biobank with ethics approval. RNA integrity and quantity were tested with an Agilent 4200 Tapestation System. A total of 100 ng RNA was processed using the KAPA mRNA HyperPrep Kit (Roche, KK8580) according to manufacturer's instructions. In brief, poly-adenylated mRNAs were isolated, chemically fragmented and primed with random hexamers, before generation of strand-specific first strand cDNA using reverse transcriptase in the presence of actinomycin D. Quality of libraries generated was accessed using High Sensitivity Agilent DNA 1000 assay (Agilent, 5067-4626)) on Agilent TapeStation 4200, before being enzymatically normalised using a Normalase assay (Swift BioSciences, 66096). Libraries were sequenced on a S1 flow cell on a NovaSeq 6000 system (Illumina, San Diego, US) at 0.8 nM using v1.5 SBS chemistry and a 50bp paired-end recipe. Approximately 20 million reads per sample were generated. Data was demultiplexed and FASTQ files generated using Illumina's bcl2fast Conversion Software v2.20. FASTQ files were then tagged with the UMI read (with UMITools) and aligned to the human genome UCSC hg38 using RNA-STAR 2.5.2b. Aligned reads were UMI deduplicated using JE-Suite (1.2.1) and reads per transcript were counted by FeatureCounts to produce a digital output of gene expression. EdgeR was used to generate counts per million (CPM), to be used for filtering lowly expressed genes. Differential expression analysis was performed using limma-voom with cut-off of adjusted p value < 0.05 for statistical significance. Package clusterProfiler (version 3.0.4) was used for GO enrichment analysis.

Single-cell RNA sequencing

Single cell RNA libraries were generated using the Chromium Single Cell 3' Reagent Kits v3 (10X Genomics, CA, USA). The cells were counted using a trypan blue exclusion count assay with a Bio-Rad Automated Cell Counter TC10 and diluted for loading onto the Chromium Controller. Loading was performed to target capture of ~8,000 Gel Bead-In-EMulsions (GEMs) per sample for downstream analysis, and samples were processed through the Chromium

Controller following the standard manufacturer's specifications. The sequencing libraries were evaluated for quality on the Agilent TapeStation (Agilent Technologies, USA), and quantified by using a Qubit 2.0 Fluorometer (Invitrogen) and pooled libraries were quantified using qPCR (Applied Biosystems) prior to loading onto an Illumina sequencing platform. The samples were sequenced at a configuration compatible with the recommended guidelines as outlined by 10X Genomics. Raw sequence data (.bcl files) generated from Illumina HiSeq was converted into fastq files and demultiplexed using the 10X Genomics' cellranger mkfastq command. Subsequent UMI and cell barcode de-convolution along with mapping to the respective genome was performed using 10X Genomics' cellranger software package to generate the final digital gene expression matrices.

Data was analysed using the Seurat package. Only cells expressing more than 200 genes and fewer than 8000 and with mitochondrial gene percentages less than 30% were kept for further analysis. In total, 19,399 and 20,535 single cells from two independent controls and from three independent USH1B patients, respectively, remained (control.1 replicate1, 6,607 cells; control.1 replicate 2, 6,315 cells; control.3, 6,477 cells; USH1B.1, 6,653; USH1B.2, 6,983 cells and USH1B.3, 6,899 cells). A total of 39934 cells were integrated with the Seurat standard protocol and the effect of mitochondrial genes were regressed out using "ScaleData" function. The top markers for each cluster were identified using the "FindConservedMarkers" function. Cell identity assignment was performed based on well-defined retinal markers and two recent single-cell sequencing studies of human retina. Full list of top marker genes used for cell assignment can be found in Table S. The "FindMarkers" function was used to identify differentially expressed genes between control and patient cells globally or in a cell/cluster-specific manner.

Data availability

RNA-seq data have been deposited in ArrayExpress under accession numbers E_MTAB-11405 (Bulk RNA-seq) and E-MTAB-11990 (single cell RNA-seq)

Supplemental References

AILION, M., HANNEMANN, M., DALTON, S., PAPPAS, A., WATANABE, S., HEGERMANN, J., LIU, Q., HAN, H. F., GU, M., GOULDING, M. Q., SASIDHARAN, N., SCHUSKE, K., HULLETT, P., EIMER, S. & JORGENSEN, E. M. 2014. Two Rab2 interactors regulate dense-core vesicle maturation. *Neuron,* 82**,** 167-80.

BRASIL, A. A., DE CARVALHO, M. D. C., GERHARDT, E., QUEIROZ, D. D., PEREIRA, M. D., OUTEIRO, T. F. & ELEUTHERIO, E. C. A. 2019. Characterization of the activity, aggregation, and toxicity of heterodimers of WT and ALS-associated mutant Sod1. *Proc Natl Acad Sci U S A,* 116**,** 25991-26000.

BRUGGER, M., BECKER-DETTLING, F., BRUNET, T., STROM, T., MEITINGER, T., LURZ, E., BORGGRAEFE, I. & WAGNER, M. 2021. A homozygous truncating variant in CCDC186 in an individual with epileptic encephalopathy. *Ann Clin Transl Neurol,* 8**,** 278-283.

DAS, A., QIAN, J. & TSANG, W. Y. 2017. USP9X counteracts differential ubiquitination of NPHP5 by MARCH7 and BBS11 to regulate ciliogenesis. *PLoS Genet,* 13**,** e1006791.

ESTEVE, P., SANDONIS, A., CARDOZO, M., MALAPEIRA, J., IBANEZ, C., CRESPO, I., MARCOS, S., GONZALEZ-GARCIA, S., TORIBIO, M. L., ARRIBAS, J., SHIMONO, A., GUERRERO, I. & BOVOLENTA, P. 2011. SFRPs act as negative modulators of ADAM10 to regulate retinal neurogenesis. *Nat Neurosci,* 14**,** 562-9.

HEISSLER, S. M. & MANSTEIN, D. J. 2012. Functional characterization of the human myosin-7a motor domain. *Cell Mol Life Sci,* 69**,** 299-311.

HUAI, J. & ZHANG, Z. 2019. Structural Properties and Interaction Partners of Familial ALS-Associated SOD1 Mutants. *Front Neurol,* 10**,** 527.

JIANG, K., FAIRLESS, E., KANDA, A., GOTOH, N., COGLIATI, T., LI, T. & SWAROOP, A. 2020. Divergent Effects of HSP70 Overexpression in Photoreceptors During Inherited Retinal Degeneration. *Invest Ophthalmol Vis Sci,* 61**,** 25.

JIN, T., TAN, X., SHI, X., LV, L., PENG, X., ZHANG, H., TANG, B., WANG, C. & YANG, M. 2021. Preliminary Findings on Proline-Rich Protein 14 as a Diagnostic Biomarker for Parkinson's Disease. *Neuromolecular Med,* 23**,** 285-291.

KABAHUMA, R. I., SCHUBERT, W. D., LABUSCHAGNE, C., YAN, D., BLANTON, S. H., PEPPER, M. S. & LIU, X. Z. 2021. Spectrum of MYO7A Mutations in an Indigenous South African Population Further Elucidates the Nonsyndromic Autosomal Recessive Phenotype of DFNB2 to Include Both Homozygous and Compound Heterozygous Mutations. *Genes (Basel),* 12.

LENASSI E, SAIHAN Z, CIPRIANI V, LE QUESNE STABEJ P, MOORE AT, LUXON LM, BITNER-GLINDZICZ M, WEBSTER AR. 2014. Natural history and retinal structure in patients with Usher syndrome type 1 owing to MYO7A mutation. *Ophthalmology*, 121(2), 580-7.

MACKAY, E. W., MOLERO, S. I., TIRATHDAS, L. H., PETERSON-MADURO, J., ZANG, J., NEUHAUSS, S. C. F., SCHULTE-MERKER, S., WILSON, S. W. 2020. Zinc dysregulation in slc30a8 (znt8) mutant zebrafish leads to blindness and disrupts bone mineralisation. *bioRxiv preprint*.

MOLLEMA, N. J., YUAN, Y., JELCICK, A. S., SACHS, A. J., VON ALPEN, D., SCHORDERET, D., ESCHER, P. & HAIDER, N. B. 2011. Nuclear receptor Rev-erb alpha (Nr1d1) functions in concert with Nr2e3 to regulate transcriptional networks in the retina. *PLoS One,* 6**,** e17494.

NGUYEN, T. T. M., MURAKAMI, Y., SHERIDAN, E., EHRESMANN, S., ROUSSEAU, J., ST-DENIS, A., CHAI, G., AJEAWUNG, N. F., FAIRBROTHER, L., REIMSCHISEL, T., BATEMAN, A., BERRY-KRAVIS, E., XIA, F., TARDIF, J., PARRY, D. A., LOGAN, C. V., DIGGLE, C., BENNETT, C. P., HATTINGH, L., ROSENFELD, J. A., PERRY, M. S., PARKER, M. J., LE DEIST, F., ZAKI, M. S., IGNATIUS, E., ISOHANNI, P., LONNQVIST, T., CARROLL, C. J., JOHNSON, C. A., GLEESON, J. G., KINOSHITA, T. & CAMPEAU, P. M. 2017. Mutations in GPAA1, Encoding a GPI Transamidase Complex Protein, Cause Developmental Delay, Epilepsy, Cerebellar Atrophy, and Osteopenia. *Am J Hum Genet,* 101**,** 856-865.

SANTOFIMIA-CASTANO, P., LAN, W., BINTZ, J., GAYET, O., CARRIER, A., LOMBERK, G., NEIRA, J. L., GONZALEZ, A., URRUTIA, R., SOUBEYRAN, P. & IOVANNA, J. 2018. Inactivation of NUPR1 promotes cell death by coupling ER-stress responses with necrosis. *Sci Rep,* 8**,** 16999.

SCHINDELIN, J., ARGANDA-CARRERAS, I., FRISE, E., KAYNIG, V., LONGAIR, M., PIETZSCH, T., PREIBISCH, S., RUEDEN, C., SAALFELD, S., SCHMID, B., et al. 2012. Fiji: an open-source platform for biological-image analysis. *Nat Methods,* 9**,** 676-82.

SHINDE, V., PITALE, P. M., HOWSE, W., GORBATYUK, O. & GORBATYUK, M. 2016. Neuronatin is a stress-responsive protein of rod photoreceptors. *Neuroscience,* 328**,** 1-8.

TELIAS, M., DENLINGER, B., HELFT, Z., THORNTON, C., BECKWITH-COHEN, B. & KRAMER, R. H. 2019. Retinoic Acid Induces Hyperactivity, and Blocking Its Receptor Unmasks Light Responses and Augments Vision in Retinal Degeneration. *Neuron,* 102**,** 574-586 e5.

YASUDA, M., TANAKA, Y., OMODAKA, K., NISHIGUCHI, K. M., NAKAMURA, O., TSUDA, S. & NAKAZAWA, T. 2016. Transcriptome profiling of the rat retina after optic nerve transection. *Sci Rep,* 6**,** 28736.
