# Supplementary material for: Molecular pathology of Usher 1B patient-derived retinal organoids at single cell resolution
Source: Stem Cell Reports. 2022 Oct 13;17(11):2421–37. doi: 10.1016/j.stemcr.2022.09.006 (PMC9669639; doi:10.1016/j.stemcr.2022.09.006)
Supplement: Document S2. Article plus supplemental information [file mmc4.pdf]

Stem Cell Reports
Article
OPEN ACCESS

Molecular pathology of Usher 1B patient-derived retinal organoids at single cell resolution

Yeh Chwan Leong,[1] Valentina Di Foggia,[1] Hema Pramod,[1] Maria Bitner-Glindzicz,[1] Aara Patel,[1] and Jane C. Sowden[1,*]
[1]UCL Great Ormond Street Institute of Child Health, University College London and NIHR Great Ormond Street Hospital Biomedical Research Centre, University College London, London WC1N 1EH, UK
*Correspondence: j.sowden@ucl.ac.uk
https://doi.org/10.1016/j.stemcr.2022.09.006

SUMMARY

Usher syndrome-associated retinitis pigmentosa (RP) causes progressive retinal degeneration, which has no cure. The pathomechanism of Usher type 1B (USH1B)-RP caused by *MYO7A* mutation remains elusive because of the lack of faithful animal models and limited knowledge of *MYO7A* function. Here, we analyzed 3D retinal organoids generated from USH1B patient-derived induced pluripotent stem cells. Increased differential gene expression occurred over time without excessive photoreceptor cell death in USH1B organoids compared with controls. Dysregulated genes were enriched first for mitochondrial functions and then proteasomal ubiquitin-dependent protein catabolic processes and RNA splicing. Single-cell RNA sequencing revealed *MYO7A* expression in rod photoreceptor and Müller glial cells corresponding to upregulation of stress responses in *NRL*$^+$ rods and apoptotic signaling pathways in *VIM*$^+$ Müller cells, pointing to the defensive mechanisms that mitigate photoreceptor cell death. This first human model for USH1B-RP provides a representation of patient retina *in vivo* relevant for development of therapeutic strategies.

INTRODUCTION

Usher syndrome (USH) is an autosomal-recessive hereditary disease that causes sensorineural hearing loss, vestibular dysfunction, and retinitis pigmentosa (RP). It is the most common hereditary disease that causes deaf-blindness, affecting 1 to 4 in 25,000 newborns (Parker and Bitner-Glindzicz, 2015). Three major USH clinical types (I–III) have been identified. USH1 is the most severe causing profound congenital hearing loss, RP beginning in the first decade of life, and balance issues. RP is characterized by progressive loss of rod photoreceptors in the retina, resulting in night blindness and tunnel vision, followed by cone photoreceptor loss, affecting central vision and eventually leading to legal blindness (Bonnet and El-Amraoui, 2012). *MYO7A* gene mutation (USH1B subtype) accounts for 40%–55% of all USH1 cases, with >2,000 disease-causing variants reported (Whatley et al., 2020). Although hearing loss can be effectively compensated for with cochlear implants, no treatments are currently available for USH1B-RP.

MYO7A is a class 7 myosin protein with an N-terminal motor domain and distinct properties (Heissler and Manstein, 2012). Like other members of the myosin superfamily, it binds actin filaments triggering ATP hydrolysis to energise its translocation. No faithful animal or human cell models of USH1B-associated RP are available to date. *Myo7a* mutant mouse models, such as *shaker1*, recapitulate hearing loss and vestibular dysfunction but not the USH1B retinal phenotype (Gibson et al., 1995). Studies using *shaker1* identified rhodopsin mislocalization and showed MYO7A in the connecting cilium along the axonemal actin, where it facilitates rhodopsin transport from the photoreceptor inner segment (IS) to the outer segment (OS) (Liu et al., 1999). The same model showed the absence of MYO7A disrupted melanosome translocation to the apical processes of retinal pigment epithelium (RPE) cells and OS phagocytosis (Gibbs et al., 2003). Soni et al. (2005) demonstrated disrupted interaction of MYO7A with lysosomes in a human RPE cell line. In addition, contrasting findings of RPE resistance (Lopes et al., 2011) and rod vulnerability (Peng et al., 2011) to light exposure were reported in *shaker1*.

Subsequent studies in humans, non-human primates, and amphibians showed MYO7A and other USH1 proteins form a linkage between the OS and calyceal processes (CP), the actin filament-filled extensions sprouting from the IS to surround the base of OS (Sahly et al., 2012). Knockout of protocadherin-15 (USH1F) and cadherin-23 (USH1D) in frogs compromised the CP structural support, causing distortion of the OS shape (Schietroma et al., 2017). Notably, *Myo7a* knockout frogs acquired developmental defects hampering further study. The lack of CP in the mouse retina plus other differences compared with the human eye, such as eye size, high rod-to-cone ratio, and the absence of a macula could explain the absence of a USH1B retinal phenotype in mouse models. A study in dogs also failed to observe retinal phenotypes (Webb et al., 2019), while vestibular dysfunction and deafness were present. Pigs with *MYO7A* mutation showed balance difficulties and suspected hearing loss, but retinal phenotypes were not studied (Derks et al., 2021). Like the frog

Crown Copyright © 2022
This is an open access article under the CC BY license (http://creativecommons.org/licenses/by/4.0/).

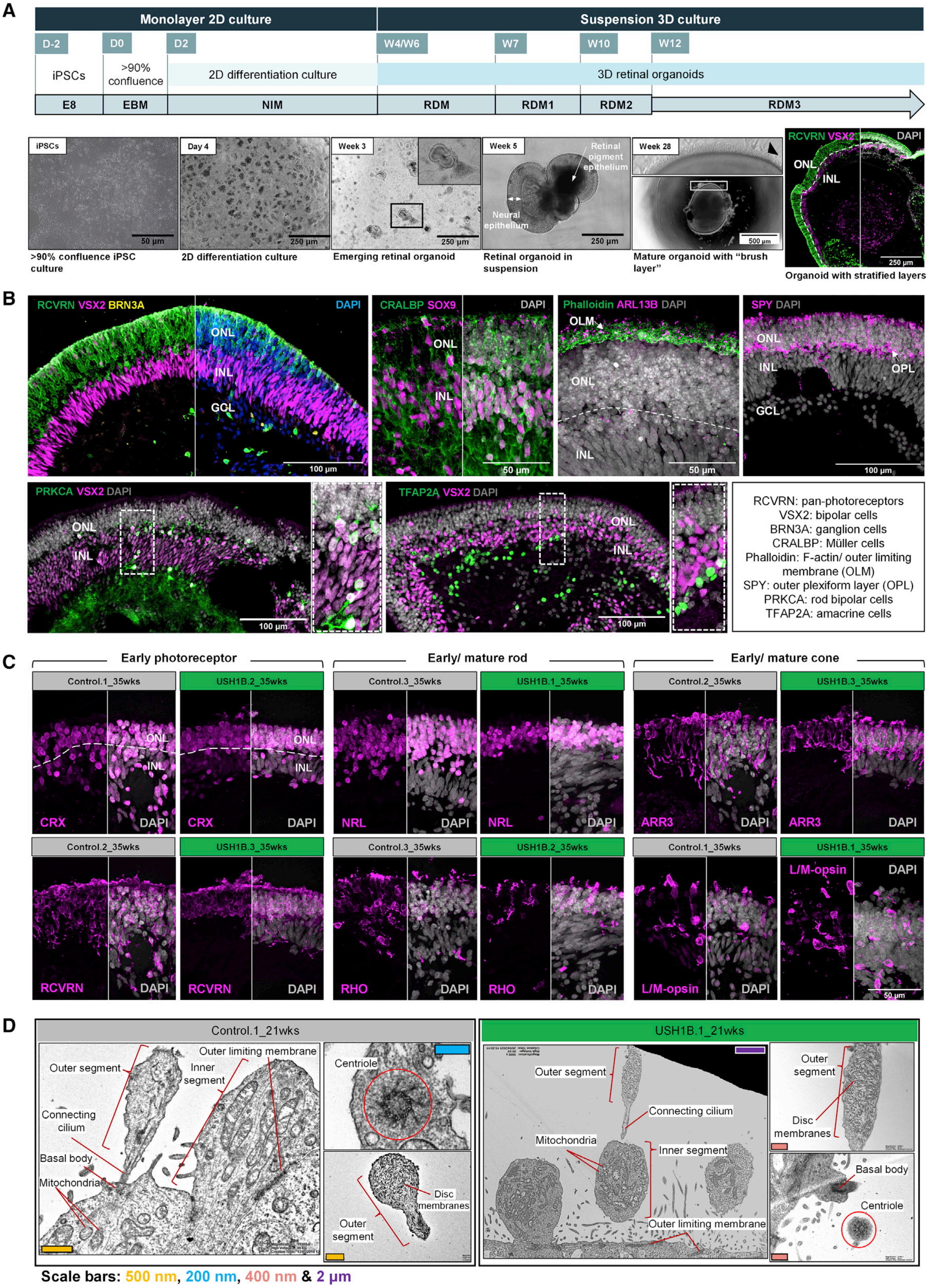

(legend on next page)

model, piglets with a homozygous nonsense *MYO7A* mutation died soon after birth.

In addition to the lack of faithful animal models, there is a wider lack of understanding of the molecular pathways leading to photoreceptor cell death that are triggered by the disease-causing mutations. Modeling USH1B with patient-derived induced pluripotent stem cells (iPSCs) offers an alternative route with potential to understand the early consequences of *MYO7A* mutation in human retinal cells. Tang et al. (2016) reported the first human USH1B cochlear hair cell model derived from iPSCs of a patient suffering sensorineural hearing loss (non-syndromic USH1B), but retinal cells have not previously been studied. Rod photoreceptor cells are highly metabolically active requiring high oxygen consumption. In RP, elevated oxidative stress is thought to accelerate rod photoreceptor cell loss and secondary cone photoreceptor death. The sensitivity of photopigments to light stress and the high turnover of OS contribute to the production and accumulation of reactive oxygen species (ROS), leading to lipid, protein and DNA damage in animal models (Punzo et al., 2012; Gallenga et al., 2021). In addition, antioxidants slow photoreceptor cell death in animal models (Komeima et al., 2006). Increased markers of oxidative stress, including elevation of endoplasmic reticulum (ER) stress markers, have been reported in non-USH1 RP patient iPSC-derived retinal cells grown in two-dimensional (2D) cultures (Jin et al., 2011, 2012; Yoshida et al., 2014) or in organoids (Tucker et al., 2013).

Here, we report the first three-dimensional (3D) retinal organoid model of USH1B generated from patient-derived iPSCs. The iPSC-derived organoids did not show evidence of cell degeneration in culture at fetal retina-equivalent stage, consistent with the onset of USH1B in childhood. However, bulk RNA sequencing (RNA-seq) and Gene Ontology (GO) enrichment analysis revealed increased aberrantly expressed genes in patient-derived cells over time. Single-cell RNA sequencing (scRNA-seq) analysis of 35 week organoids identified adaptive responses to stress specifically in USH1B rods, and enrichment for apoptotic signaling pathways in USH1B Müller cells. Together these findings indicate the presence of a mutation-induced molecular pathology in patient-derived retinal cells and provide evidence of increased adaptive responses in patient cells that maintain cellular homeostasis and prevent photoceptor cell death in the *in vitro* organoid system.

RESULTS

Derivation of USH1B patient iPSCs

Dermal skin fibroblasts were collected from three USH1B patients for reprogramming to iPSCs. We confirmed that the generated USH1B-iPSCs harbored the same *MYO7A* mutations identified in their fibroblast counterparts using Sanger sequencing (Figure S1). Patient 2 (USH1B.2) and patient 3 (USH1B.3) are siblings, both harboring a c.6070C>T (p.Arg2024*) nonsense mutation and a c.223G>C (p.Asp75His) missense mutation. Patient 1 (USH1B.1) harbored a c.1996C>T (p.Arg666*) nonsense mutation and an acceptor splice site mutation, c.133-2A>G, in intron 3 (IVS3-2A-G) p.(=), causing the skipping of canonical acceptor splice site and shortening of exon 4 by seven highly conserved amino acids (Figure S1). All iPSCs showed characteristics of pluripotent cells in culture and expressed pluripotency markers as shown by immunohistochemistry (IHC), while maintaining genome integrity as indicated by SNP array (Figure S2).

Generation of 3D retinal organoids from control and USH1B iPSCs

USH1B and control iPSCs were differentiated to 3D retinal organoids *in vitro* using a published protocol (Cuevas et al., 2021; Gonzalez-Cordero et al., 2017) (Figure 1A). By 21 weeks, organoids contained stratified neuroepithelium with multiple cell layers that were characterized using IHC (Figure 1B). Photoreceptors in the outer nuclear layer (ONL) stained for RCVRN. The inner nuclear layer (INL) stained for VSX2, marking bipolar cells (BCs), and the ganglion cell layer (GCL) was identified by BRN3A. Müller cells

Figure 1. Generation of 3D retinal organoids from USH1B patient and control iPSCs

(A) Schematic of retinal differentiation protocol (upper panel). Bright-field images show the appearance of organoid cultures at different stages (lower panel). Black arrowhead, segment-like structures. Representative examples from Control.1 and USH1B.2.

(B and C) IHC of 21 and 35 week retinal organoids shows the presence of various retinal cell types and proper cytoarchitecture (B) and early and late photoreceptor markers (C); photoreceptors, RCVRN; bipolar cells/retinal progenitor cells, VSX2; BRN3A, retinal ganglion cells; Müller cells, SOX9 and VIM; phalloidin, outer limiting membrane (OLM); ARL13B, connecting cilium; SYP, inner plexiform layer (INL); PKCα, bipolar cells (see Table S2 for full protein names). Representative examples shown from N > 3 differentiations per iPSC line, n > 3 organoids per differentiation. RPE, retinal pigment epithelium; NE, neural epithelium; ONL, outer nuclear layer; INL, inner nuclear layer; GCL, ganglion cell layer; OLM, outer limiting membrane.

(D) Transmission electron micrographs show the presence of photoreceptor subcellular structures and separating photoreceptor cell body and segments in both control and patient 21 week retinal organoids. N = 2 differentiations per iPSC line, n > 2 organoids per differentiation.

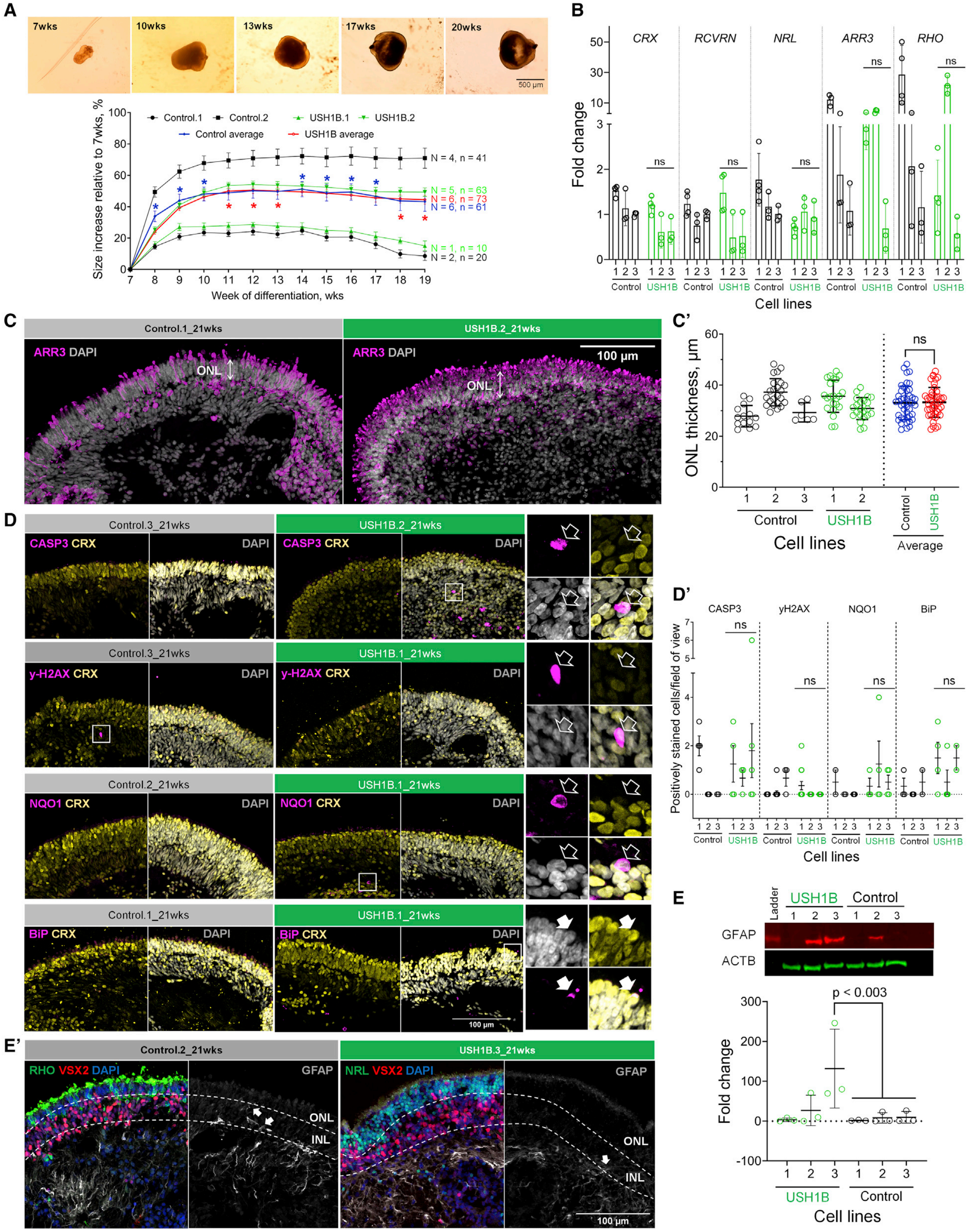

(legend on next page)

(MCs) were identified using IHC for CRALBP and SOX9. F-actin (phalloidin) staining confirmed the presence of outer limiting membrane (OLM) and the outer apical polarity of the organoid. Synaptophysin (SYP) staining indicated the outer plexiform layer (OPL). Rod BCs (PRKCA) and amacrine cells (ACs) (TFAP2A) were also present. At 35 weeks, both control and USH1B photoreceptors expressed pan-photoreceptor (RCVRN and CRX), rod (NRL), and cone (ARR3) markers, as well as rod-specific (RHO) and cone-specific (L/M-opsin) photopigments (Figure 1C). Photoreceptors acquired subcellular features, such as connecting cilium (ARL13B and PCNT), and rod (PRPH2) or cone (GNAT2) outer segment structures by 28 weeks of differentiation (Figure S2). Transmission electron micrographs of organoids showed the presence of OLM, mitochondria-rich IS, developing OS containing disc membrane-like structures, connecting cilia, basal body and centrioles (with 9 + 0 microtubule arrangement) (Figure 1D). Overall, USH1B iPSCs differentiated as effectively as control iPSCs to produce retinal organoids that resembled native human retina.

Absence of RP degenerative features in USH1B retinal organoids

To determine if USH1B organoids reproduce degenerative phenotypes in patients, first, we compared the size of USH1B and control organoids from 7 to 19 weeks. Using 7 weeks as a baseline we found that although control and USH1B organoid size changed at variable rates, no USH1B-specific abnormality was observed (Figure 2A). To assess whether USH1B organoids lost photoreceptors to degeneration by 21 weeks, we determined the level of photoreceptor marker gene expression and quantified the thickness of the ONL (where photoreceptor nuclei reside). No significant differences were observed in expression of *CRX*, *RCVRN*, *NRL*, *ARR3* and *RHO* (Figure 2B). Similarly, we found no aberrant ONL thinning in USH1B organoids, suggesting excessive photoreceptor death was not present (Figures 2C and 2C′).

To assess if USH1B organoids showed increased cellular stress, we analyzed expression of γH2AX and 8-hydroxydeoxyguanosine (8-OHdG) (DNA damage/stress), NQO1 (oxidative stress), BiP (ER stress), acrolein (lipid oxidation), and CASP3 (apoptosis) using IHC (Figure 2D). Each marker was co-stained with CRX. Overall, stress markers were scarce. When present, they rarely colocalized with CRX, suggesting that stressed cells were not photoreceptors. No significant elevation was observed in USH1B organoids (Figure 2D′). We did not detect any cells positive for 8-OHdG and acrolein (data not shown).

Reactive gliosis is a secondary event to photoreceptor loss in RP, characterized by overexpression of glial fibrillary acidic protein (GFAP) and the disruption of OLM integrity. It was reported in iPSC-derived retinal organoids from RP patients harboring *RPGR* mutation (Deng et al., 2018) and in an *Rpgr*$^{-/-}$ mouse model (Megaw et al., 2017). We quantified GFAP protein levels using western blot and distribution using IHC of 21 week organoids (Figures 2E and 2E′). One patient line (USH1B.3) showed a significantly higher level of GFAP. However, IHC revealed restricted expression of GFAP within inner retina (at the pseudo-GCL and occasionally INL), resembling the pattern in healthy mouse retina (Hippert et al., 2021), in both control and USH1B organoids (including USH1B.3). Overall, USH1B patient organoids did not display marked characteristics of degeneration, photoreceptor cell death, oxidative stress or reactive gliosis.

RNA sequencing analysis showed resemblance of late-stage retinal organoids to fetal retina

To investigate the development of retinal organoids and the "closeness" of their global transcriptomes to native retina, we performed bulk RNA sequencing analysis on control and USH1B iPSCs (day 0), early-stage (7 weeks), mid-stage

Figure 2. Comparison of USH1B and control retinal organoids to identify USH1B-associated degenerative features

(A) Top: bright-field images shows organoid growth; images from Control.1. Changes in organoid size over time relative to 7 weeks. Independent-samples t test was performed at each time point. *$p < 0.001$, control > USH1B when in blue, USH1B > control when in red.

(B) qRT-PCR analysis of photoreceptor marker expression by 21 week patient and control organoids. One-way ANOVA with Dunnett correction. Fold change relative to *GAPDH* expression and normalized to Control.3. N = 3, n = 2–3.

(C and C′) Quantification of ONL thickness in 21 week USH1B and control organoids. Representative images show one field of view. IHC for ARR3 defined the ONL. N = 2, n = 2. Data points are measurements from one field of view. Independent-samples t test.

(D) Oxidative stress marker expression in 21 week organoids. Representative images showing one field of view. Insets show colocalization of stress markers with CRX (empty arrow, not colocalized; filled arrow, colocalized).

(D′) Quantification of double-positive cells for CRX and stress markers. Data point, number of organoids. N = 2. One-way ANOVA with Dunnett correction.

(E) GFAP expression in 21 week organoids. Quantification of GFAP expression with immunoblotting; fold change relative to ACTB expression and normalized to Control.1. N = 3, n = 5–8.

(E′) Representative images of IHC of GFAP, with RHO, VSX2, and NRL. Observed in >3 organoids per iPSC line.

In (A), bars denote mean ± SEM; in (B)–(E), bars denote mean ± SD. N, independent differentiation per iPSC line; n, organoids per differentiation; ns, not significant.

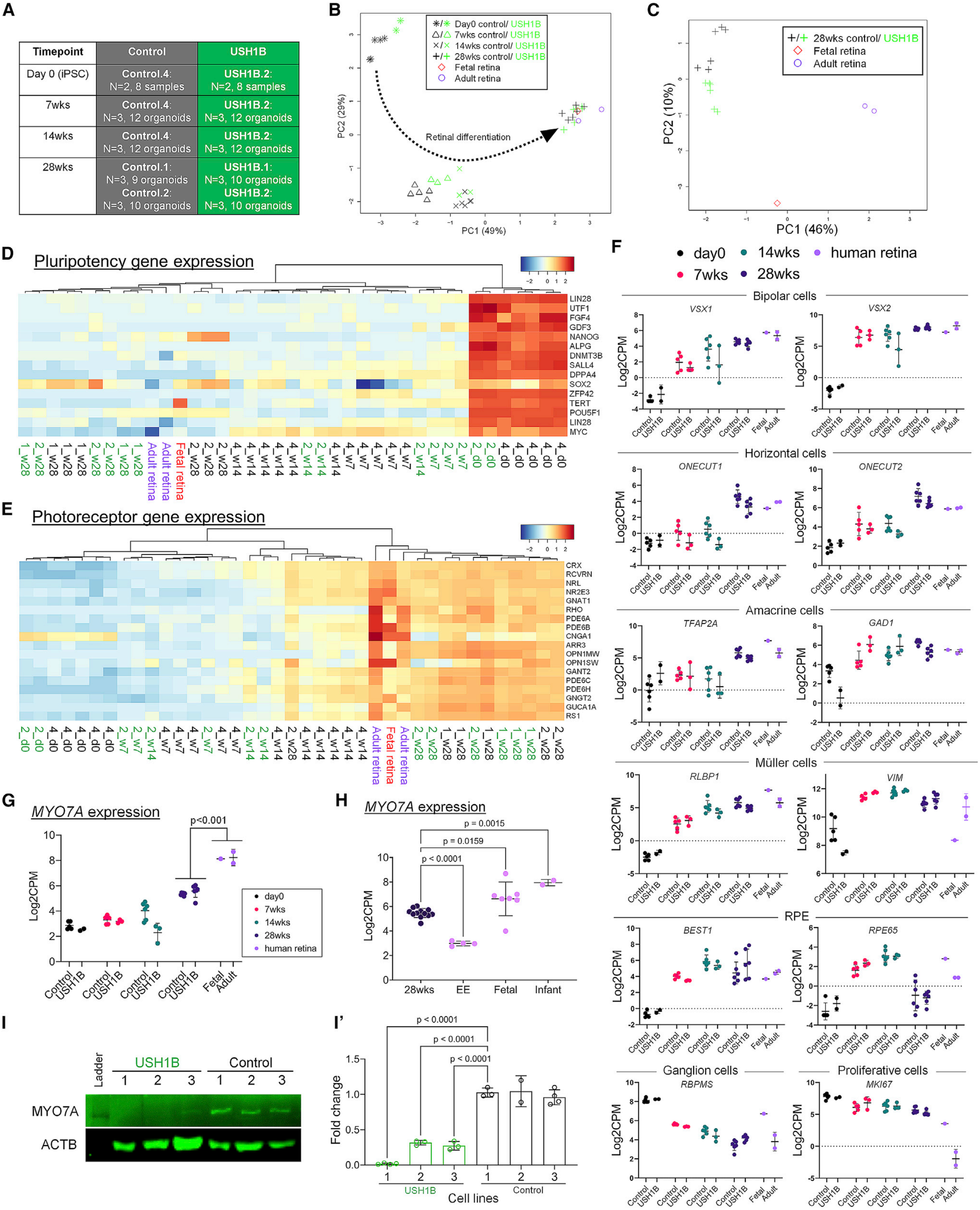

(legend on next page)

(14 weeks), and late-stage (28 weeks) organoids, and human 20 weeks post-conception fetal and adult retina (Figure 3A). Principal-component analysis (PCA) revealed close correlation of organoids according to their respective time of differentiation (Figure 3B). Day 0 iPSCs clustered furthest from the fetal and adult retina along PC1, followed by 7 and 14 weeks, indicating the progression of retinal differentiation. All 28 weeks, organoids clustered closer to fetal than to adult retina (Figure 3C). Pluripotency gene expression showed a drastic downregulation in 7, 14, and 28 weeks organoids compared with day 0 iPSCs, indicating loss of the pluripotency state (Figure 3D). The expression of well-defined photoreceptor markers was upregulated starting at 14 weeks and further elevated at 28 weeks to resemble the level of human retina (Figure 3E).

A gradual increase of expression was also observed in marker genes of other retinal cell types, including BCs, horizontal cells (HCs), ACs, and MCs, in the organoids (Figure 3F). Decreased *RBPMS* expression over time signaled diminishing RGC number, consistent with the trajectory in human retina and other retinal organoid studies (Cowan et al., 2020). Varying levels of *BEST1* and *RPE65* were due to the sporadic presence of RPE. Analysis of *MYO7A* expression at different stages showed levels increased concurrently with retinal maturation but were significantly lower than human retina (Figure 3G).

To more accurately map the 28 weeks organoids to an *in vivo* developmental stage, we compared the 28 weeks organoids with a published bulk RNA-seq dataset of human embryonic eye, fetal, and infant retina (Table S3) (Mellough et al., 2019). Figure S3 shows that the 28 weeks organoids were more mature than embryonic eye and were similar to fetal/infant retina with respect to retinal cell marker expression. *MYO7A* expression showed a similar upward trend across *in vivo* developmental stages (Figure 3H), with higher levels in 28 weeks organoids compared with embryonic eyes.

As difference in *MYO7A* mRNA level was not apparent between USH1B and control organoids, we sought to investigate MYO7A protein levels in order to validate the predicted effect of *MYO7A* mutations on protein synthesis. Immunoblotting of 21 weeks organoids (Figures 3I and 3I′) with an antibody (DSHB 138-1) that targets amino acids 927–1203 of MYO7A should detect products from both alleles (if translated; p.Asp75His, p.Arg2024*) of USH1B.2 and USH1B.3 but only one allele (splice site mutation c.133-2A>G) of USH1B.1 (Figure S3). Residual MYO7A expression (fold change 0.30 ± 0.05) was detected in USH1B.2 and USH1B.3 by the p.Asp75His allele, while mRNA products from the p.Arg2024* allele were not detected suggesting nonsense-mediated mRNA decay. The USH1B.1 allele did not give rise to detectable protein.

To summarize, we showed that USH1B and control organoids developed and matured to acquire global transcriptomes at 28 weeks, largely similar to fetal retina. Profiles of expression of retinal cell markers were similar to adult retina suggesting completion of retinal histogenesis. Considering the onset of USH1B in childhood, the similarity to fetal retina is consistent with the absence of signs of photoreceptor cell degeneration in the USH1B organoids.

RNA-seq analysis revealed dysregulated GO terms in mid- and late-stage USH1B retinal organoids

To identify mutation-induced transcriptomic defects in USH1B organoids, we performed differential expression (DE) analysis comparing the transcriptomes of control and USH1B organoids (control versus USH1B). A total of 803, 1,866, 3,999, and 9,184 differentially expressed genes (DEGs) (adjusted p value < 0.05) were identified at day 0, 7 weeks, 14 weeks, and 28 weeks, respectively (Figure 4A; Table S4), suggesting that control and USH1B organoids became more different over time, coinciding with the rise of *MYO7A* expression. Indeed, PCA and Pearson's correlation coefficient showed clear separation of control and USH1B 28 week organoids (Figures 4B and 4C). Considering RP is progressive, we hypothesised that aberrant transcriptional patterns, if present, should persist, if not

Figure 3. RNA-seq analysis showed similarity of 28 week retinal organoid to fetal retina

(A) The number of organoids collected at four different time points of retinal differentiation of USH1B and control iPSC lines: day 0, 7 weeks, 14 weeks, and 28 weeks, for bulk RNA-seq.

(B and C) PCA shows similarity of transcriptomes of organoids and human retina. Dotted arrow indicates the path of retinal differentiation.

(D and E) Heatmap of pluripotency (D) and photoreceptor (E) marker gene expression in organoids and human retina; control, black; USH1B, green.

(F) Gene expression of markers of specific retinal cell types indicated, bipolar, horizontal, amacrine, Müller, retinal ganglion, RPE, and proliferative cells.

(G) Expression of *MYO7A* in organoids at different time points and in human retina.

(H) Expression of *MYO7A* in 28 weeks organoids compared with human embryonic eye (EE), fetal and infant retina (GEO: GSE98370).

(I and I′) Western blot of MYO7A in 28 weeks organoids. Fold change shown relative to ACTB expression and normalized to Control.1. MYO7A, 254 kDa; ACTB, 42 kDa. N = 3 independent differentiations per line; n = 5–8 organoids per differentiation.

In (F)–(I), mean fold change or mean $\log_2$CPM ± SD; one-way ANOVA with Dunnett correction.

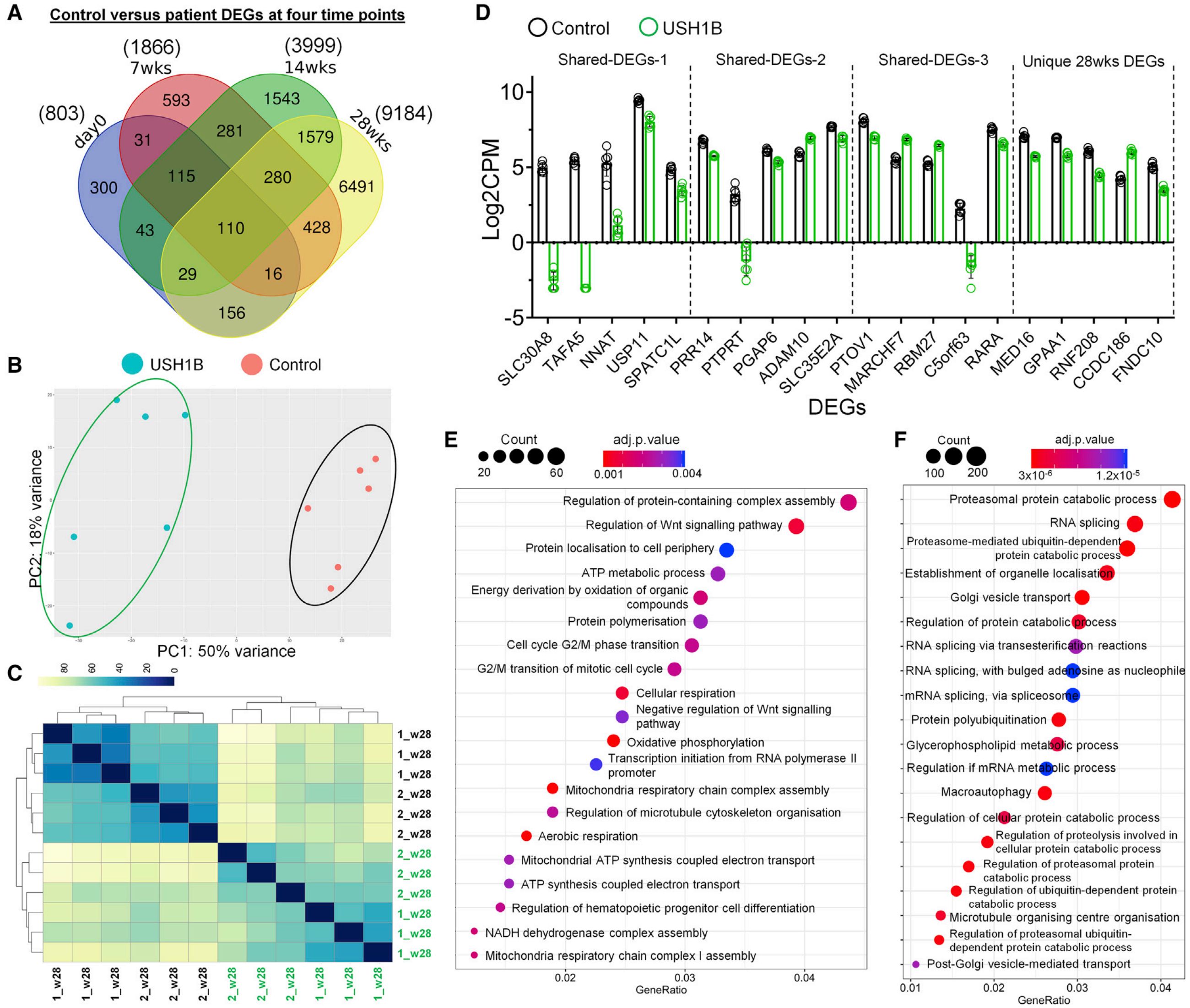

Figure 4. Differential expression analysis comparing control and USH1B iPSCs, 7, 14, and 28 week retinal organoids

(A) Venn diagram shows number of significant (adjusted p value < 0.05) DEGs between control and patient at four different time points and number of overlapped DEGs.

(B and C) PCA (B) and heatmap of Pearson's correlation coefficient matrix (C) (control in black and USH1B in green) indicate clear separation of control and patient 28 weeks retinal organoids on the basis of global transcriptional profiles.

(D) The expression of the top five DEGs (ranked by adjusted p value) in four overlapped gene lists $\log_2$CPM ± SD.

(E and F) Top 20 most significantly enriched (by adjusted p value) GO biological processes terms analyzing (E) shared DEGs-3 and (F) 28 weeks unique DEGs.

increase, at later time points. Therefore, to investigate the onset of molecular pathology associated with *MYO7A* mutation preceding degeneration, we performed GO enrichment analysis on DEGs shared across (1) all four time points (110 DEGs), (2) 7 weeks onward (280 DEGs), (3) 14 week onward (1,579 DEGs), and (4) unique DEGs for 28 weeks (6,491 DEGs), hereafter referred to as shared DEGs-1 to DEGs-3 and 28 weeks unique DEGs (Figure 4A; Table S4). The top five DEGs for each group are shown in Figure 4D (Table S4 indicates links of these top five genes to retina and/or neurodegeneration).

No GO enrichment (q < 0.05) was identified in shared DEGs-1 and shared DEGs-2 (Table S4). Shared DEGs-3 showed enrichment for biological process terms chiefly relating to mitochondria functions, Wnt signaling pathway, protein localization, and cell cycle (Figure 4E). DEGs unique to 28 weeks were enriched for protein ubiquitination, RNA splicing, cellular transport, and organelle

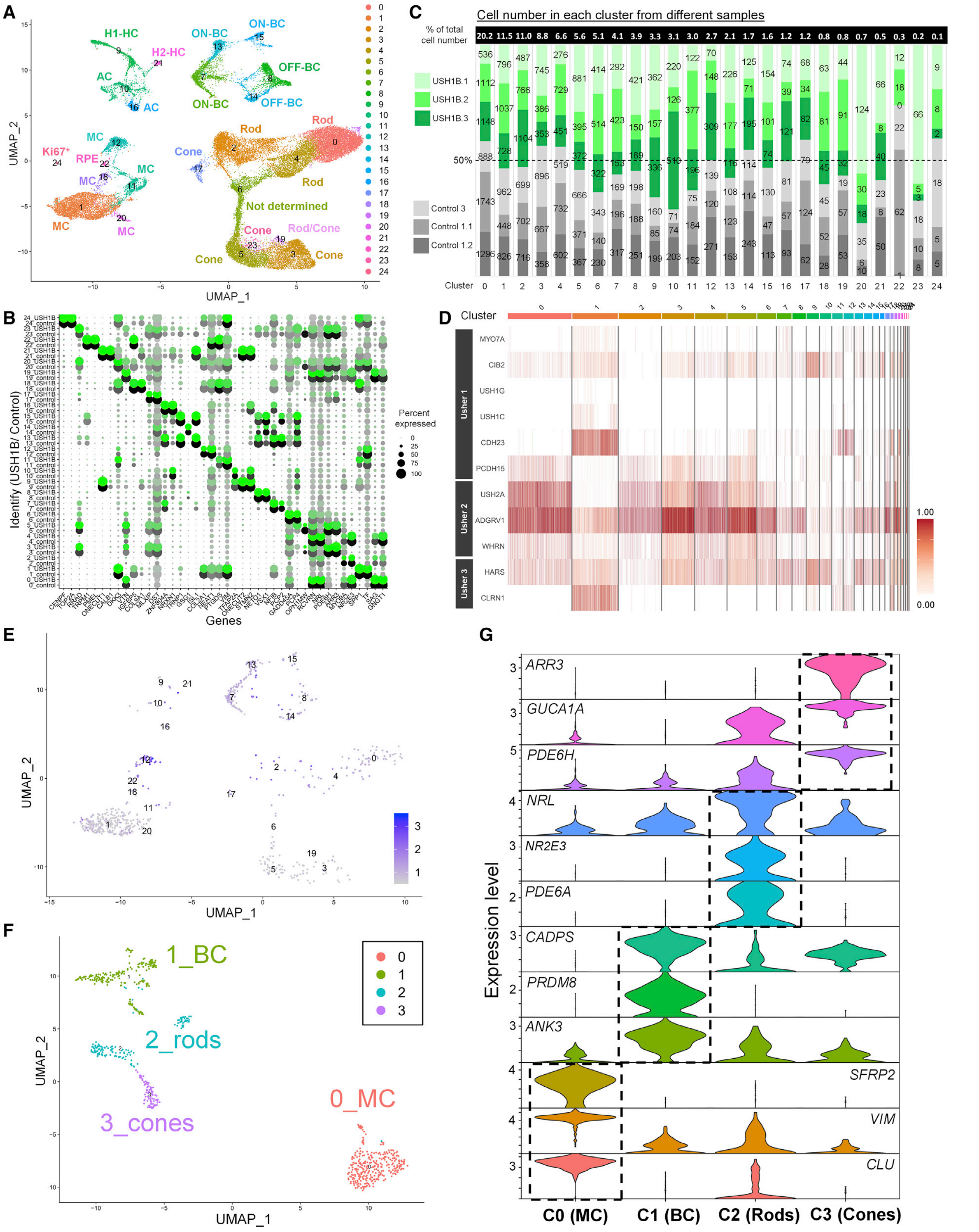

(legend on next page)

localization (Figure 4F; Table S4). Both the latter are relevant to the current understandings of MYO7A function. Consistently, GO enrichment analysis of molecular function terms identified similar findings as shown in Table S4. Ubiquitination is a critical pathway controlling proteosome homeostasis and regulating degradation of damaged or misfolded proteins, and its dysfunction is associated with a range of neurodegenerative diseases that affect the retina (Campello et al., 2013). Figure S4 shows the set of genes in the Kyoto Encyclopedia of Genes and Genomes (KEGG) ubiquitin-mediated proteolysis pathway that are dysregulated in the 28 weeks organoids.

In summary, through bulk RNA-seq analysis, we identified dysregulated genes in USH1B organoids, many of which have implications in retinal dystrophy or other neurodegenerative diseases. These aberrant transcriptomic patterns represent clues for the dissection of USH1B disease mechanisms and serve as potential early disease biomarkers.

Single-cell RNA sequencing showed the expression of Usher genes at a single-cell level

We next sought to elucidate the site of action of *MYO7A* by establishing a cell atlas of USH1B organoids. The 35 weeks USH1B and control organoids were subjected to single-cell RNA sequencing. The 35 weeks time point was chosen because a recent study showed that organoids acquired a stable "developed state" between 30 and 38 weeks and did not mature further (Cowan et al., 2020). The RNA of 19,399 and 20,535 single cells from control and USH1B, respectively, was sequenced. Dimension reduction of the 39,934 single-cell transcriptomes with uniform manifold approximation and projection (UMAP) identified 25 clusters, encompassing 7 major retinal cell types, including rods, cones, BCs, ACs, HCs, MCs, and RPE (Figure 5A), assigned on the basis of the expression of well-defined markers (Figure 5B, Table S5). Further analyses showed the presence of cell subtypes, including rod subtypes differentiated by the expression of *MYO9A*, and BC and HC subtypes (Yi et al., 2021) (Figure S5). Each of the 25 clusters was well represented in the six organoid samples (Figure 5C).

Next, we mapped the expression of all USH-causative genes to organoids (Figure 5D). For USH1 genes, *MYO7A*, *USH1G*, *USH1C*, and *CDH23* were highly expressed by MCs (cluster 1), while *CIB2* was ubiquitously expressed and *PCDH15* specific to photoreceptors. For USH2 genes, high *USH2A* expression was detected in photoreceptors, while *ADGRV1* and *WHRN* were detected in all cell types. Last, USH3 gene *CLRN1* was highly expressed in MCs and *HARS* was ubiquitously expressed. To further determine identities of cells expressing *MYO7A*, we performed dimension reduction on a total of 756 *MYO7A*-expressing cells (Figures 5E and 5F; Table S5). Four distinct cell clusters were identified expressing top markers indicative of MCs, BCs, rods, and cones (Figure 5G; Table S5). Despite previous *MYO7A* studies showing exclusive expression by RPE and photoreceptors, in particular rods, the expression of *MYO7A* by MCs reported in this study is consistent with a recent scRNA-seq analysis of human retina (Cowan et al., 2020). These findings indicate *MYO7A* could play roles in other cell types, which are undocumented and could contribute to pathology.

Single-cell DE analysis identified early signs of degeneration specific to rods and upregulation of apoptotic pathways in MC

Finally, we sought to identify molecular defects specific to individual retinal cell types. Degeneration of rods is the primary clinical event in RP, hence we examined directly if rods show evidence of altered transcriptomes. NRL is an essential rod-specific transcription factor. We compared the transcriptomes of 3,072 and 2,325 *NRL*-expressing (NRL^+) rods from control and USH1B organoids, respectively. All cells were found in clusters 0, 2 and 4, previously identified as rods (Figure 6A; Table S5). DE analysis identified 207 DEGs (Table S5; genes with links to retina and/or neurodegeneration are indicated). GO enrichment analysis identified biological process terms associated with cellular responses to chemical and oxidative stress as well as hydrogen peroxide metabolic process (Figures 6B and 6C; Table S5). Genes encoding pro-apoptotic factor (*BNIP3*), antioxidant enzymes (*PRDX1*, *PRDX2*, and *PRDX5*), and free radical scavenging enzyme (*SOD1*) were overexpressed in USH1B NRL^+ cells, suggesting ongoing adaptive responses to cell stress (Figure 6C). The same events were

Figure 5. Single-cell RNA sequencing of control and USH1B patient 35 weeks retinal organoids

(A) UMAP shows the presence of 25 clusters, encompassing 7 major retinal cell types on the basis of the transcriptomes of 39,934 single cells.

(B) Dot plot illustrates the expression of the top two markers (by adjusted p value) by each cluster. Table S5 provides the top genes in each cluster.

(C) Bar plot represents the number of cells from each organoid sample in each single-cell cluster.

(D) Heatmap shows the expression of the known Usher syndrome causative genes across 25 clusters.

(E–G) UMAPs show the distribution of 756 $MYO7A^+$ single cells across 25 clusters identified in Figure 5A (E); re-clustering of these 756 cells identified four distinct clusters, Müller cells (MCs), bipolar cells (BCs), rods and cones (F); violin plots show the expression of the top three markers (dashed boxes) by each of the four clusters (G).

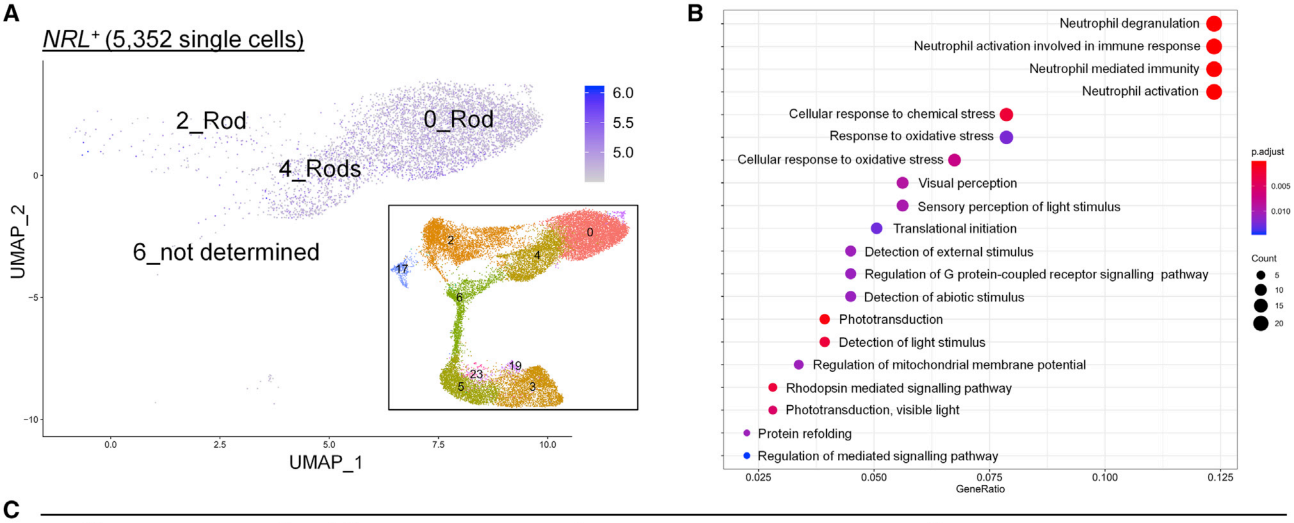

C

ID	Description	qvalue	Genes
GO:0062197	Cellular response to chemical stress	0.002	BNIP3/CHCHD2/DDX3X/HSPA1A/HSPA1B/JUN/NCOA7/PRDX1/PRDX2/PRDX5/ RACK1/SOD1/STK39/TPM1
GO:0034599	Cellular response to oxidative stress	0.006	BNIP3/CHCHD2/HSPA1A/HSPA1B/JUN/NCOA7/PRDX1/PRDX2/PRDX5/RACK1/SOD1/ TPM1
GO:0006979	Response to oxidative stress	0.01	BNIP3/CHCHD2/HSPA1A/HSPA1B/JUN/LDHA/NCOA7/PRDX1/PRDX2/PRDX4/PRDX5/ RACK1/SOD1/TPM1
GO:0034614	Cellular response to reactive oxygen species	0.01	BNIP3/JUN/PRDX1/PRDX2/PRDX5/RACK1/SOD1/TPM1
GO:0042743	Hydrogen peroxide metabolic process	0.02	PRDX1/PRDX2/PRDX4/PRDX5/SOD1

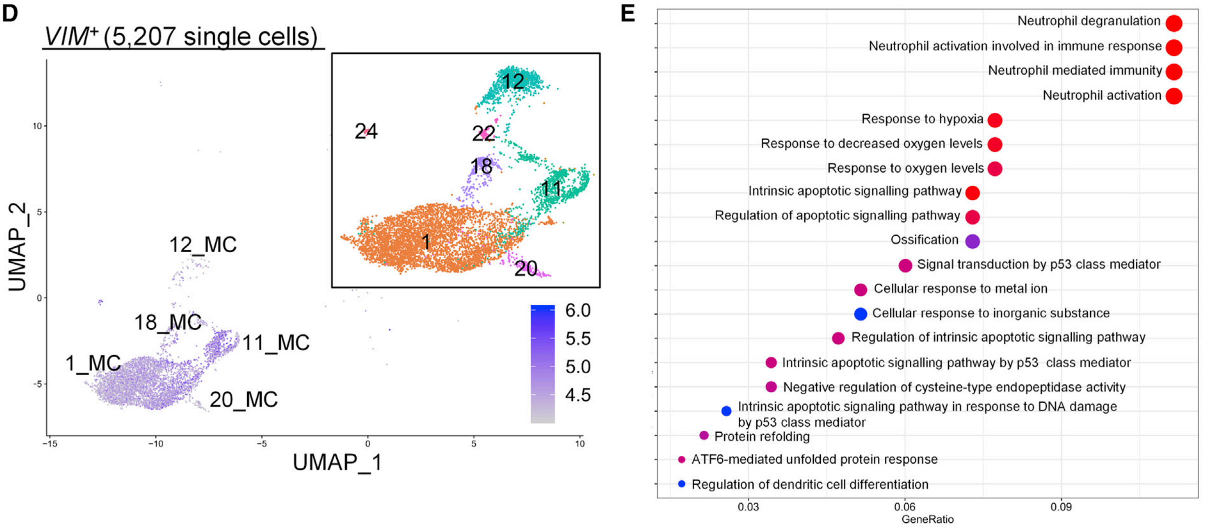

F

ID	Description	qvalue	Genes
GO:0097193	Intrinsic apoptotic signaling pathway	0.0002	CD44/CEBPB/DDIT3/DDIT4/DDX3X/ENO1/HSPA1A/IFI16/MCL1/NOL3/NUPR 1/PERP/PHLDA3/TRIB3/UBB/YBX3
GO:2001233	Regulation of apoptotic signaling pathway	0.002	CD44/DDIT3/DDX3X/ENO1/HSPA1A/HSPA1B/ITM2C/LGALS3/MCL1/NGFR/N OL3/NUPR1/PRELID1/TIMP3/UBB/YBX3
GO:0070997	Neuron death	0.004	APOE/BTG2/CEBPB/DDIT3/DDIT4/EGR1/FOS/HSPA5/MCL1/MDK/NGFR/NQ O1/NUPR1/PTPRZ1/UBB
GO:0072331	Signal transduction by p53 class mediator	0.004	BTG2/CD44/CDKN1B/CSNK2B/DDIT4/GADD45A/IFI16/MYO6/NUPR1/PERP/ PHLDA3/RBBP7/UBB
GO:2001242	Regulation of intrinsic apoptotic signaling pathway	0.004	CD44/DDIT3/DDX3X/ENO1/HSPA1A/MCL1/NOL3/NUPR1/UBB/YBX3

(legend on next page)

not detected when analyzing 3,013 *ARR3*+ (cone arrestin) cones, suggesting that molecular pathology was limited to rods (Figures S6A–S6C; Table S5).

In view of the new interest in MCs as a *MYO7A*-expressing cell type, we performed the same analysis on a total of 5,207 vimentin (*VIM*+) MCs (2,392 control and 2,815 USH1B) (Figures 6D–6F; Table S5). DE analysis identified 255 DEGs, which were enriched for biological process terms for regulation of apoptotic signaling pathways (Table S5). Last, we analyzed 2,277 BCs identified by expression of *GRIK1* (1,136 cells) and *PCP2* (1,141 cells) (Figures S6D–S6F). A total of 157 DEGs were identified, which were not enriched in either apoptotic- or stress response-related GO terms (Table S5). We noted that the abundant presence of ribosomal genes and ribosome-related GO terms in the *ARR3*+ and *PCP2*+/*GRIK1*+ cell-specific GO enrichment analyses might represent technical artifacts previously reported as common to scRNA-seq (Nowakowski et al., 2017).

In summary, we identified stress responses in rods, but not cones, consistent with clinical observations that the former is first to degenerate in USH1B-RP. Analysis of *VIM*+ MCs showed USH1B MC exhibited apoptotic pathway-related transcription activities. These results suggest our organoid model reproduces a pre-death mutation-induced state of rod photoreceptors *in vitro*. It is also instrumental for further investigation into the vulnerability of MC.

DISCUSSION

By coupling iPSC technology and *in vitro* retinal differentiation protocols, we established the first retinal organoid disease model for USH1B. Bulk RNA-seq analysis revealed the transcriptomes of USH1B patient organoids were progressively distinct from controls after extended time in culture, coinciding with the gradual rise of *MYO7A* expression. Dysregulation of genes associated with mitochondrial function were followed by genes enriched for protein ubiquitination, RNA splicing, and cellular transport functions. Elevated cellular responses to stress were detected exclusively in USH1B rods, suggesting organoids were modeling a molecular pathology underlying early RP manifestations. This is the first such analysis reported. By identifying mutation-induced altered transcriptomes in patient-derived retinal cells that predispose rods to cell stress, this study provides the first clues into molecular mechanisms underlying USH1B-RP.

Limitations of USH1B patient-derived 3D retinal organoid model

Recent studies comparing retinal organoids with human retina suggested *in vitro* differentiation recapitulates the rate of retina development *in vivo* (Cowan et al., 2020). Consistently, bulk and scRNA-seq as well as mapping of the 11 USH genes to specific retinal cell types demonstrated the faithfulness of our organoid model as a surrogate for USH1B disease modeling. Despite these similarities, the level of *MYO7A* expression in 28 weeks control organoids was lower than that of the adult retina. This may be due to a reduction or absence of cellular activities involving MYO7A in the organoids. For example, the absence of juxtaposition of RPE to photoreceptors precludes OS phagocytosis. In addition, immature electrophysiological responses commonly reported in retinal organoids suggest attenuated phototransduction, possibly due to reduced protein trafficking and lack of the visual cycle (Hallam et al., 2018). Last, a lack of development of CP could abolish the need for USH1 protein linkage formation between OS and CP. The lack of retinal vasculature, blood supply and resident microglia in the organoid model also limits modeling of mutation-induced inflammatory responses, which may be important for understanding the notable identification of dysregulation of genes associated with neutrophil activation pathways in the USH1B rods and MC in this study (Table S5). In summary, although organoids provide valuable new insights into retinal disease pathology, further improvement on complexity and functionality of the model could improve representation of disease conditions *in vivo*.

Figure 6. Single-cell RNA-seq identified early signs of degeneration in rods and Müller cells

(A) UMAP shows distribution of 5,352 *NRL*+ cells only in the clusters in the "photoreceptor island" (mostly rod clusters) identified in Figure 5A (inset).

(B) Dot plot shows top 20 significantly enriched GO biological processes terms on the basis of analysis of 207 DEGs comparing control and *NRL*+ cells.

(C) Table shows details of five significantly enriched biological process GO terms associated with stress response ranked by adjusted p value Table S5.

(D) UMAP shows localization of 5,207 *VIM*+ cells in the clusters identified as MC in Figure 5A (inset).

(E) Dot plot shows top 20 significantly enriched biological processes GO terms on the basis of analysis of 255 DEGs comparing control and USH1B *VIM*+ cells.

(F) Table shows details of the top five significantly enriched GO biological processes terms associated with apoptotic signaling (ranked by adjusted p value) Table S5.

Retinal cell types expressing *MYO7A*

Previous studies of USH1B focused entirely on the role of MYO7A in rods and the RPE on the basis of the clinical phenotype and distribution pattern of MYO7A in animal models. Analysis of *MYO7A*$^{+}$ cells in this study identified the majority of them as MCs, followed by BCs, rods, and cones. This raises the question of why, when *MYO7A* is mutated, rods are the first cell type to degenerate, which is typical of all RP clinically. Significantly, our scRNA-seq data showed heightened defensive responses against stress exclusively by rods, modeling their clinical susceptibility.

In addition, we found activation of apoptotic signaling pathways in USH1B patient MCs. MC death has not been widely studied or considered a primary event in RP. However, OCT examination of USH1B patient retina identified increased OLM visibility likely attributed to MC response to nearby photoreceptor cell stress or death (Jacobson et al., 2009). The same study also attributes OPL and INL thickening in USH1B patient retina to MC hypertrophy or hyperreactivity. Interrogating the role of MYO7A in the various cell types in which it is expressed would be important to further understand USH1B disease mechanism. To date we have not been able to confirm MYO7A protein localization in MCs using IHC with three different antibodies (Table S2) (data not shown), suggesting post-transcriptional control mechanisms and/or low or transient protein.

Dysregulated GO terms over time: Potential disease markers of mutation-induced state

Over time in culture, USH1B organoids displayed increased numbers of DEGs associated with the ubiquitin-proteosome system (UPS), which is known to function in response to stress. Notably we found TOPORS and UBR1 dysregulated in the USH1B organoids (GO:0010498; Figure 4F; Table S4); mutation of the former is a cause of autosomal dominant RP (Chakarova et al., 2007), and the latter promotes rhodopsin protein degradation in the OS via the UPS during retinal inflammation (Ozawa et al., 2008). Altered protein trafficking of rhodopsin with a P23H mutation overwhelms the proteasome machinery due to UPS inefficiency in degrading the variant, leading to malfunction in various cellular pathways (Illing et al., 2002). The 28 weeks DEGs were also enriched for RNA splicing (GO:0008380). More than 25% of retinal disease-causing mutations alter splicing patterns (Bacchi et al., 2014). Mutation of the ubiquitously expressed pre-mRNA processing factors (PRPFs) accounts for 15% of autosomal-dominant RP (Wang et al., 2019). Several members of PRPFs (*PRPF18*, *PRPF31* and *PRPF39*) were dysregulated in the USH1B organoids (Table S4). A recent study showed that USH1G (SANS) interacts with PRPFs to facilitate the transport of small nuclear ribonucleoprotein during pre-RNA splicing (Yildirim et al., 2021). *PRPF31* mutation causes autosomal-dominant RP (RP11) and adversely affects primary cilia formation in photoreceptors and RPE function (Buskin et al., 2018). Last, with regard to cellular transport, *KIF3A* and *KIF3B* (a RP gene) along with other members of the kinesin family (GO:0048193) as well as components of the intraflagellar transport system (GO:0030705), including *IFT20* were dysregulated in USH1B organoids.

Antioxidant enzymes differentially expressed between the control and patient retinal cells

Single-cell analysis of USH1B patient cells showed elevation of defensive cellular responses exclusively in rods. One interpretation of these findings is that patient cells were more sensitive to cellular stress compared with controls. Antioxidant enzymes including members from the peroxiredoxins (PRDXs) and superoxide dismutase (SOD) families were upregulated in rods. PRDX isoforms have previously been reported in different retinal cell types and organelles (Chidlow et al., 2016) and implicated in neurodegenerative and retinal diseases (Zheng et al., 2012; Kubo et al., 2009; Kim et al., 2001). However, PRDXs are not known to be expressed in rods under normal physiological conditions, and the absence of H_2O_2 scavenging PRDXs may contribute to susceptibility of rods to degeneration. *Sod1*$^{-/-}$ mouse retina showed mitochondrial degeneration in both INL and ONL and AMD-like phenotypes (Hashizume et al., 2008). *BNIP3* was another notable dysregulated defensive gene identified as part of the cellular response to oxidative stress in *NRL*$^{+}$ rods in patient organoids (GO:0034599; Figure 6C). *BNIP3* encodes a pro-apoptotic factor that interacts with anti-apoptotic proteins. A HIF-1α/BNIP3 pathway is neuroprotective against inner retinal neurodegeneration (Kunimi et al., 2021), whereas overexpression of BNIP3/FUNDC1 signaling reversed cell stress and death caused by HIF-1α inhibition in an hypoxic retinal model (Sun et al., 2021).

Cellular responses in rods against stress: One-hit model

A cumulative damage model is often used to describe cell death in neurodegenerative disorders, whereby gene mutation causes the accumulation of secondary damage in cells, increasing the likelihood of a cell dying over time. In contrast, the one-hit model described by Clarke et al. (2000) proposes that the risk of a cell's dying remains constant. Cells harboring gene mutations acquire a nearly normal homeostatic state called the mutant steady state (MSS), whereby a critical, but not lethal, alteration of gene activity that regulates pre-death reactions is present. Initiation of cell death only occurs when rare and random

catastrophic events cause a surge of pre-death molecules to pass a critical cell-death threshold.

The altered transcriptomes of USH1B cells are consistent with an MSS being established in organoids. The enrichment of cell death-related pathways and cellular stress pathways (pre-death markers) may reflect the deployment of stress-mitigating enzymes to maintain the MSS and suppress cells from reaching the cell-death threshold. We speculate that the ample supply of nutrients *in vitro* and minimal exposure to light damage may limit the occurrence of catastrophic events leading to significant levels of cell death. The introduction of various stressors that an individual would normally experience in life, including exposure to natural light, free radicals, and reactive oxygen species, into the organoid system may better mimic the aging USH1B degenerative disease state.

EXPERIMENTAL PROCEDURES

Additional details are provided in the supplemental information.

iPSC generation

The study was approved by the National Research Ethics Committee London-Dulwich (11/LO/1243). Fibroblasts from USH1B patients and controls were reprogrammed to iPSC using Sendai virus. Details of mutations are given in Figure S1.

Retinal organoid differentiation and analysis

iPSCs were grown to 90% confluency before switching to embryoid body (EB) medium on day 0, neural induction medium (NIM) on day 2, followed by retinal differentiation medium (RDM) containing various supplements (RDM1–3) for retinal maturation and long-term culture of organoids. EB, NIM, RDM, and RDM1–3 are described in Table S1. Organoids were analyzed using RT-PCR, transmission electron microscopy (TEM), and western blot as described in supplemental information.

Immunohistochemistry

Retinal organoids were fixed with 4% paraformaldehyde (PFA) solution and cryosections of frozen organoids analyzed using immunohistochemistry. Primary and secondary antibodies are listed in the Table S2.

Bulk RNA sequencing

RNA (100 ng) from organoids was processed using the KAPA mRNA Hyper−Prep Kit (KK8580; Roche) and libraries sequenced on a S1 flow cell on a NovaSeq 6000 system (Illumina). FASTQ files from 20 million reads per sample were aligned to the human genome UCSC hg38 using RNA-STAR 2.5.2b. Differential expression analysis was performed using limma-voom with cut-off of adjusted p value < 0.05 for statistical significance. Package clusterProfiler (version 3.0.4) was used for GO enrichment analysis.

Single-cell RNA sequencing

Single-cell RNA libraries were generated using the Chromium Single Cell 3' Reagent Kits version 3 (10X Genomics) and analyzed on an Illumina sequencing platform with 10X Genomics Cell Ranger software to generate digital gene expression matrices. Data from 39,934 cells were analyzed using Seurat. The “FindMarkers” function was used to identify differentially expressed genes between control and patient cells globally or in a cell/cluster-specific manner.

Statistical analysis

Data were analyzed and graphs created using GraphPad Prism (version 9.2.0) or Microsoft Excel (version 2110). One-way ANOVA with Dunnett correction was performed on data from at least three independent experiments, represented as mean ± SD, unless stated otherwise. A p value < 0.05 or an adjusted p value < 0.05 was considered to indicate statistical significance. Graphical abstract and schematics were created in BioRender.com.

Data and code availability

RNA-seq data have been deposited in ArrayExpress under accession numbers ArrayExpress: E_MTAB-11405 (Bulk RNA-seq) and ArrayExpress: E-MTAB-11990 (single-cell RNA-seq).

SUPPLEMENTAL INFORMATION

Supplemental information can be found online at https://doi.org/10.1016/j.stemcr.2022.09.006.

AUTHOR CONTRIBUTIONS

Conceptualisation, J.C.S., V.D.F., and Y.C.L.; Methodology, Y.C.L. and J.C.S.; Investigation, Y.C.L. and V.D.F.; Collection of Patient Samples, M.B.G.; Data Analysis and Interpretation, Y.C.L., A.P., V.D.F., and J.C.S.; Single-Cell Library Preparations, A.P.; Resources, H.P., A.P., M.B.G., and J.C.S.; Validation, Y.C.L.; Writing, Y.C.L. and J.C.S.; Final Approval of Manuscript, J.C.S.; Supervision, J.C.S.; Funding, J.C.S.

ACKNOWLEDGMENTS

This work was supported by the National Institute for Health Research (NIHR) Great Ormond Street Hospital (GOSH) Biomedical Research Centre, Medical Research Council (MRC/S036237/1), GOSH Children’s Charity and Newlife the Charity for Disabled Children. The views expressed are those of the authors and not necessarily those of the National Health Service (NHS), the NIHR or the Department of Health. This paper is dedicated to the memory of Professor Maria Bitner-Glindzicz, who died on September 20, 2018. We thank the individuals who donated samples for this study; D. Moulding at the Great Ormond Street Institute of Child Health (GOSICH) for assistance with microscopy; K. Venner at University College London (UCL) for transmission electron microscopy; D. Kelberman, UCL Genomics and Genewiz (Azenta Life Science) for RNA sequencing; and all other colleagues at UCL GOSICH who provided expert technical support and resources for this project.

CONFLICT OF INTERESTS

The authors declare no competing interests.

Received: March 31, 2022
Revised: September 14, 2022
Accepted: September 15, 2022
Published: October 13, 2022

REFERENCES

Bacchi, N., Casarosa, S., and Denti, M.A. (2014). Splicing-correcting therapeutic approaches for retinal dystrophies: where endogenous gene regulation and specificity matter. Invest. Ophthalmol. Vis. Sci. *55*, 3285–3294. https://doi.org/10.1167/iovs.14-14544.

Bonnet, C., and El-Amraoui, A. (2012). Usher syndrome (sensorineural deafness and retinitis pigmentosa): pathogenesis, molecular diagnosis and therapeutic approaches. Curr. Opin. Neurol. *25*, 42–49. https://doi.org/10.1097/wco.0b013e32834ef8b2.

Buskin, A., Zhu, L., Chichagova, V., Basu, B., Mozaffari-Jovin, S., Dolan, D., Droop, A., Collin, J., Bronstein, R., Mehrotra, S., et al. (2018). Disrupted alternative splicing for genes implicated in splicing and ciliogenesis causes PRPF31 retinitis pigmentosa. Nat. Commun. *9*, 4234. https://doi.org/10.1038/s41467-018-06448-y.

Campello, L., Esteve-Rudd, J., Cuenca, N., and Martin-Nieto, J. (2013). The ubiquitin-proteasome system in retinal health and disease. Mol. Neurobiol. *47*, 790–810. https://doi.org/10.1007/s12035-012-8391-5.

Chakarova, C.F., Papaioannou, M.G., Khanna, H., Lopez, I., Waseem, N., Shah, A., Theis, T., Friedman, J., Maubaret, C., Bujakowska, K., et al. (2007). Mutations in TOPORS cause autosomal dominant retinitis pigmentosa with perivascular retinal pigment epithelium atrophy. Am. J. Hum. Genet. *81*, 1098–1103. https://doi.org/10.1086/521953.

Chidlow, G., Wood, J.P.M., Knoops, B., and Casson, R.J. (2016). Expression and distribution of peroxiredoxins in the retina and optic nerve. Brain Struct. Funct. *221*, 3903–3925. https://doi.org/10.1007/s00429-015-1135-3.

Clarke, G., Collins, R.A., Leavitt, B.R., Andrews, D.F., Hayden, M.R., Lumsden, C.J., and Mcinnes, R.R. (2000). A one-hit model of cell death in inherited neuronal degenerations. Nature *406*, 195–199. https://doi.org/10.1038/35018098.

Cowan, C.S., Renner, M., De Gennaro, M., Gross-Scherf, B., Goldblum, D., Hou, Y., Munz, M., Rodrigues, T.M., Krol, J., Szikra, T., et al. (2020). Cell types of the human retina and its organoids at single-cell resolution. Cell *182*, 1623–1640.e34. https://doi.org/10.1016/j.cell.2020.08.013.

Cuevas, E., Holder, D.L., Alshehri, A.H., Treguier, J., Lakowski, J., and Sowden, J.C. (2021). NRL(-/-) gene edited human embryonic stem cells generate rod-deficient retinal organoids enriched in S-cone-like photoreceptors. Stem Cell. *39*, 414–428. https://doi.org/10.1002/stem.3325.

Deng, W.L., Gao, M.L., Lei, X.L., Lv, J.N., Zhao, H., He, K.W., Xia, X.X., Li, L.Y., Chen, Y.C., Li, Y.P., et al. (2018). Gene correction reverses ciliopathy and photoreceptor loss in iPSC-derived retinal organoids from retinitis pigmentosa patients. Stem Cell Rep. *10*, 2005. https://doi.org/10.1016/j.stemcr.2018.05.012.

Derks, M.F.L., Megens, H.J., Giacomini, W.L., Groenen, M.A.M., and Lopes, M.S. (2021). A natural knockout of the MYO7A gene leads to pre-weaning mortality in pigs. Anim. Genet. *52*, 514–517. https://doi.org/10.1111/age.13068.

Gallenga, C.E., Lonardi, M., Pacetti, S., Violanti, S.S., Tassinari, P., Di Virgilio, F., Tognon, M., and Perri, P. (2021). Molecular mechanisms related to oxidative stress in retinitis pigmentosa. Antioxidants *10*. https://doi.org/10.3390/antiox10060848.

Gibbs, D., Kitamoto, J., and Williams, D.S. (2003). Abnormal phagocytosis by retinal pigmented epithelium that lacks myosin VIIa, the Usher syndrome 1B protein. Proc. Natl. Acad. Sci. USA *100*, 6481–6486. https://doi.org/10.1073/pnas.1130432100.

Gibson, F., Walsh, J., Mburu, P., Varela, A., Brown, K.A., Antonio, M., Beisel, K.W., Steel, K.P., and Brown, S.D.M. (1995). A type VII myosin encoded by the mouse deafness gene shaker-1. Nature *374*, 62–64. https://doi.org/10.1038/374062a0.

Gonzalez-Cordero, A., Kruczek, K., Naeem, A., Fernando, M., Kloc, M., Ribeiro, J., Goh, D., Duran, Y., Blackford, S.J.I., Abelleira-Hervas, L., et al. (2017). Recapitulation of human retinal development from human pluripotent stem cells generates transplantable populations of cone photoreceptors. Stem Cell Rep. *9*, 820–837. https://doi.org/10.1016/j.stemcr.2017.07.022.

Hallam, D., Hilgen, G., Dorgau, B., Zhu, L., Yu, M., Bojic, S., Hewitt, P., Schmitt, M., Uteng, M., Kustermann, S., et al. (2018). Human-induced pluripotent stem cells generate light responsive retinal organoids with variable and nutrient-dependent efficiency. Stem Cell. *36*, 1535–1551. https://doi.org/10.1002/stem.2883.

Hashizume, K., Hirasawa, M., Imamura, Y., Noda, S., Shimizu, T., Shinoda, K., Kurihara, T., Noda, K., Ozawa, Y., Ishida, S., et al. (2008). Retinal dysfunction and progressive retinal cell death in SOD1-deficient mice. Am. J. Pathol. *172*, 1325–1331. https://doi.org/10.2353/ajpath.2008.070730.

Heissler, S.M., and Manstein, D.J. (2012). Functional characterization of the human myosin-7a motor domain. Cell. Mol. Life Sci. *69*, 299–311. https://doi.org/10.1007/s00018-011-0749-8.

Hippert, C., Graca, A.B., Basche, M., Kalargyrou, A.A., Georgiadis, A., Ribeiro, J., Matsuyama, A., Aghaizu, N., Bainbridge, J.W., Smith, A.J., et al. (2021). RNAi-mediated suppression of vimentin or glial fibrillary acidic protein prevents the establishment of Muller glial cell hypertrophy in progressive retinal degeneration. Glia *69*, 2272–2290. https://doi.org/10.1002/glia.24034.

Illing, M.E., Rajan, R.S., Bence, N.F., and Kopito, R.R. (2002). A rhodopsin mutant linked to autosomal dominant retinitis pigmentosa is prone to aggregate and interacts with the ubiquitin proteasome system. J. Biol. Chem. *277*, 34150–34160. https://doi.org/10.1074/jbc.m204955200.

Jacobson, S.G., Aleman, T.S., Sumaroka, A., Cideciyan, A.V., Roman, A.J., Windsor, E.A.M., Schwartz, S.B., Rehm, H.L., and Kimberling, W.J. (2009). Disease boundaries in the retina of patients with Usher syndrome caused by MYO7A gene mutations. Invest. Ophthalmol. Vis. Sci. *50*, 1886–1894. https://doi.org/10.1167/iovs.08-3122.

Jin, Z.B., Okamoto, S., Osakada, F., Homma, K., Assawachananont, J., Hirami, Y., Iwata, T., and Takahashi, M. (2011). Modeling retinal degeneration using patient-specific induced pluripotent stem cells. PLoS One *6*, e17084. https://doi.org/10.1371/journal.pone.0017084.

Jin, Z.B., Okamoto, S., Xiang, P., and Takahashi, M. (2012). Integration-free induced pluripotent stem cells derived from retinitis pigmentosa patient for disease modeling. Stem Cells Transl. Med. *1*, 503–509. https://doi.org/10.5966/sctm.2012-0005.

Kim, S.H., Fountoulakis, M., Cairns, N., and Lubec, G. (2001). Protein levels of human peroxiredoxin subtypes in brains of patients with Alzheimer's disease and Down syndrome. J. Neural. Transm. *Suppl*, 223–235. https://doi.org/10.1007/978-3-7091-6262-0_18.

Komeima, K., Rogers, B.S., Lu, L., and Campochiaro, P.A. (2006). Antioxidants reduce cone cell death in a model of retinitis pigmentosa. Proc. Natl. Acad. Sci. USA *103*, 11300–11305. https://doi.org/10.1073/pnas.0604056103.

Kubo, E., Singh, D.P., Fatma, N., and Akagi, Y. (2009). TAT-mediated peroxiredoxin 5 and 6 protein transduction protects against high-glucose-induced cytotoxicity in retinal pericytes. Life Sci. *84*, 857–864. https://doi.org/10.1016/j.lfs.2009.03.019.

Kunimi, H., Lee, D., Ibuki, M., Katada, Y., Negishi, K., Tsubota, K., and Kurihara, T. (2021). Inhibition of the HIF-1α/BNIP3 pathway has a retinal neuroprotective effect. FASEB J. *35*, e21829. https://doi.org/10.1096/fj.202100572r.

Liu, X., Udovichenko, I.P., Brown, S.D., Steel, K.P., and Williams, D.S. (1999). Myosin VIIa participates in opsin transport through the photoreceptor cilium. J. Neurosci. *19*, 6267–6274. https://doi.org/10.1523/jneurosci.19-15-06267.1999.

Lopes, V.S., Gibbs, D., Libby, R.T., Aleman, T.S., Welch, D.L., Lillo, C., Jacobson, S.G., Radu, R.A., Steel, K.P., and Williams, D.S. (2011). The Usher 1B protein, MYO7A, is required for normal localization and function of the visual retinoid cycle enzyme, RPE65. Hum. Mol. Genet. *20*, 2560–2570. https://doi.org/10.1093/hmg/ddr155.

Megaw, R., Abu-Arafeh, H., Jungnickel, M., Mellough, C., Gurniak, C., Witke, W., Zhang, W., Khanna, H., Mill, P., Dhillon, B., et al. (2017). Gelsolin dysfunction causes photoreceptor loss in induced pluripotent cell and animal retinitis pigmentosa models. Nat. Commun. *8*, 271. https://doi.org/10.1038/s41467-017-00111-8.

Mellough, C.B., Bauer, R., Co.llin, J., Dorgau, B., Zerti, D., Dolan, D.W.P., Jones, C.M., Izuogu, O.G., Yu, M., Hallam, D., et al. (2019). An integrated transcriptional analysis of the developing human retina. Development *146*, dev169474. https://doi.org/10.1242/dev.169474.

Nowakowski, T.J., Bhaduri, A., Pollen, A.A., Alvarado, B., Mostajo-Radji, M.A., Di Lullo, E., Haeussler, M., Sandoval-Espinosa, C., Liu, S.J., Velmeshev, D., et al. (2017). Spatiotemporal gene expression trajectories reveal developmental hierarchies of the human cortex. Science *358*, 1318–1323. https://doi.org/10.1126/science.aap8809.

Ozawa, Y., Nakao, K., Kurihara, T., Shimazaki, T., Shimmura, S., Ishida, S., Yoshimura, A., Tsubota, K., and Okano, H. (2008). Roles of STAT3/SOCS3 pathway in regulating the visual function and ubiquitin-proteasome-dependent degradation of rhodopsin during retinal inflammation. J. Biol. Chem. *283*, 24561–24570. https://doi.org/10.1074/jbc.m802238200.

Parker, M., and Bitner-Glindzicz, M. (2015). Genetic investigations in childhood deafness. Arch. Dis. Child. *100*, 271–278. https://doi.org/10.1136/archdischild-2014-306099.

Peng, Y.W., Zallocchi, M., Wang, W.M., Delimont, D., and Cosgrove, D. (2011). Moderate light-induced degeneration of rod photoreceptors with delayed transducin translocation in shaker1 mice. Invest. Ophthalmol. Vis. Sci. *52*, 6421–6427. https://doi.org/10.1167/iovs.10-6557.

Punzo, C., Xiong, W., and Cepko, C.L. (2012). Loss of daylight vision in retinal degeneration: are oxidative stress and metabolic dysregulation to blame? J. Biol. Chem. *287*, 1642–1648. https://doi.org/10.1074/jbc.r111.304428.

Sahly, I., Dufour, E., Schietroma, C., Michel, V., Bahloul, A., Perfettini, I., Pepermans, E., Estivalet, A., Carette, D., Aghaie, A., et al. (2012). Localization of Usher 1 proteins to the photoreceptor calyceal processes, which are absent from mice. J. Cell Biol. *199*, 381–399. https://doi.org/10.1083/jcb.201202012.

Schietroma, C., Parain, K., Estivalet, A., Aghaie, A., Boutet De Monvel, J., Picaud, S., Sahel, J.A., Perron, M., El-Amraoui, A., and Petit, C. (2017). Usher syndrome type 1-associated cadherins shape the photoreceptor outer segment. J. Cell Biol. *216*, 1849–1864. https://doi.org/10.1083/jcb.201612030.

Soni, L.E., Warren, C.M., Bucci, C., Orten, D.J., and Hasson, T. (2005). The unconventional myosin-VIIa associates with lysosomes. Cell Motil Cytoskeleton *62*, 13–26. https://doi.org/10.1002/cm.20080.

Sun, Y., Wen, F., Yan, C., Su, L., Luo, J., Chi, W., and Zhang, S. (2021). Mitophagy protects the retina against anti-vascular endothelial growth factor therapy-driven hypoxia via hypoxia-inducible factor-1α signaling. Front. Cell Dev. Biol. *9*, 727822. https://doi.org/10.3389/fcell.2021.727822.

Tang, Z.H., Chen, J.R., Zheng, J., Shi, H.S., Ding, J., Qian, X.D., Zhang, C., Chen, J.L., Wang, C.C., Li, L., et al. (2016). Genetic correction of induced pluripotent stem cells from a deaf patient with MYO7A mutation results in morphologic and functional recovery of the derived hair cell-like cells. Stem Cells Transl. Med. *5*, 561–571. https://doi.org/10.5966/sctm.2015-0252.

Tucker, B.A., Mullins, R.F., Streb, L.M., Anfinson, K., Eyestone, M.E., Kaalberg, E., Riker, M.J., Drack, A.V., Braun, T.A., and Stone, E.M. (2013). Patient-specific iPSC-derived photoreceptor precursor cells as a means to investigate retinitis pigmentosa. Elife *2*, e00824. https://doi.org/10.7554/elife.00824.

Wang, A.L., Knight, D.K., Vu, T.T.T., and Mehta, M.C. (2019). Retinitis pigmentosa: review of current treatment. Int. Ophthalmol. Clin. *59*, 263–280. https://doi.org/10.1097/iio.0000000000000256.

Webb, A.A., Ruhe, A.L., and Neff, M.W. (2019). A missense mutation in MYO7A is associated with bilateral deafness and vestibular dysfunction in the Doberman pinscher breed. Can. J. Vet. Res. *83*, 142–148.

Whatley, M., Francis, A., Ng, Z.Y., Khoh, X.E., Atlas, M.D., Dilley, R.J., and Wong, E.Y.M. (2020). Usher syndrome: genetics and molecular links of hearing loss and directions for therapy.

Front. Genet. *11*, 565216. https://doi.org/10.3389/fgene.2020.565216.

Yi, W., Lu, Y., Zhong, S., Zhang, M., Sun, L., Dong, H., Wang, M., Wei, M., Xie, H., Qu, H., et al. (2021). A single-cell transcriptome atlas of the aging human and macaque retina. Natl. Sci. Rev. *8*, nwaa179. https://doi.org/10.1093/nsr/nwaa179.

Yildirim, A., Mozaffari-Jovin, S., Wallisch, A.K., Schafer, J., Ludwig, S.E.J., Urlaub, H., Luhrmann, R., and Wolfrum, U. (2021). SANS (USH1G) regulates pre-mRNA splicing by mediating the intra--nuclear transfer of tri-snRNP complexes. Nucleic Acids Res. *49*, 5845–5866. https://doi.org/10.1093/nar/gkab386.

Yoshida, T., Ozawa, Y., Suzuki, K., Yuki, K., Ohyama, M., Akamatsu, W., Matsuzaki, Y., Shimmura, S., Mitani, K., Tsubota, K., and Okano, H. (2014). The use of induced pluripotent stem cells to reveal pathogenic gene mutations and explore treatments for retinitis pigmentosa. Mol. Brain *7*, 45. https://doi.org/10.1186/1756-6606-7-45.

Zheng, Q., Ren, Y., Tzekov, R., Zhang, Y., Chen, B., Hou, J., Zhao, C., Zhu, J., Zhang, Y., Dai, X., et al. (2012). Differential proteomics and functional research following gene therapy in a mouse model of Leber congenital amaurosis. PLoS One *7*, e44855. https://doi.org/10.1371/journal.pone.0044855.

Stem Cell Reports, Volume *17*

Supplemental Information

Molecular pathology of Usher 1B patient-derived retinal organoids at single cell resolution

Yeh Chwan Leong, Valentina Di Foggia, Hema Pramod, Maria Bitner-Glindzicz, Aara Patel, and Jane C. Sowden

A

Patient	Mutation			
	Allele 1		Allele 2	
	Gene	Protein	Gene	Protein
USH1B.1	c.133-2A>G	Acceptor splice site mutation, intron3 (IVS3-2A-G)	c.1996C>T	p.Arg666*
USH1B.2	c.223G>C	p.Asp75His	c.6070C>T	p.Arg2024*
USH1B.3	c.223G>C	p.Asp75His	c.6070C>T	p.Arg2024*

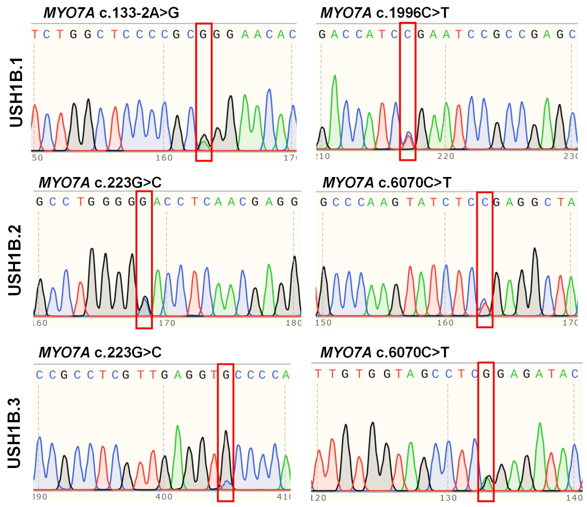

B

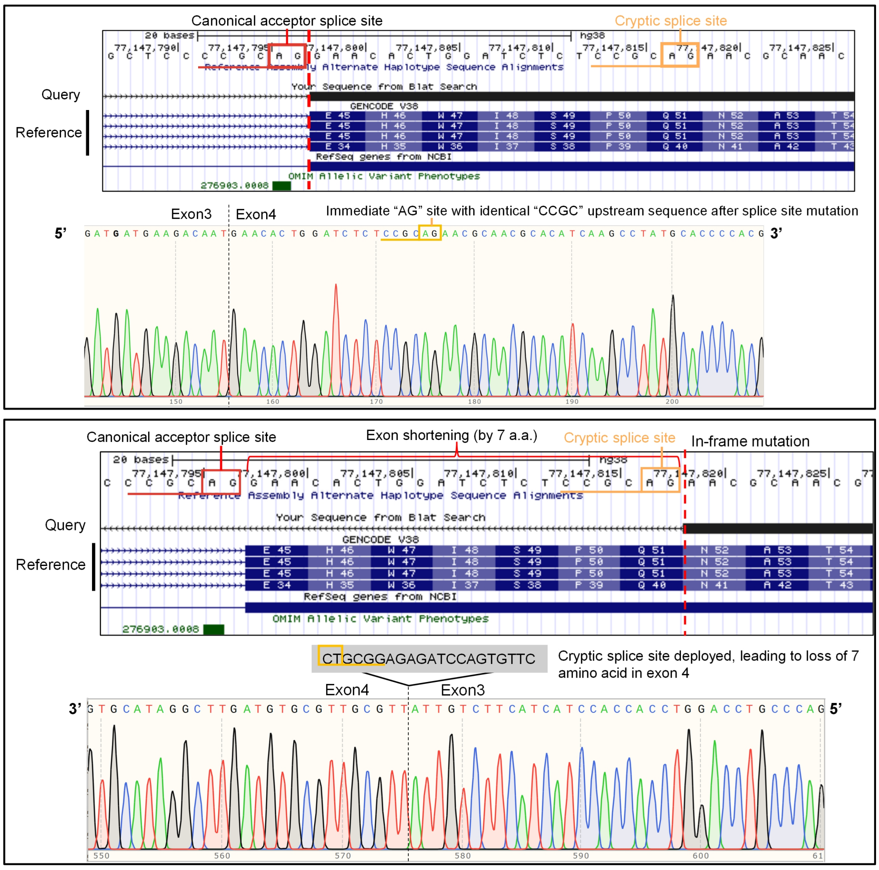

Figure S1 Confirmation of patient *MYO7A* mutations in patient-derived iPSC. Related to experimental procedure. A) (Top) Details of mutations harboured by three USH1B patients (Lenassi et al., 2014). (bottom) Sanger Sequencing confirmed patient MYO7A mutations in patient iPSCs. **B)** Sanger sequencing of cDNA from USH1B.1 patient showed deletion of 7 amino acid as a result of an acceptor splice site mutation, c.133-2A>G. (Top) sequence of a wild type allele. (bottom) sequence of allele with c.133-2A>G mutation.

A

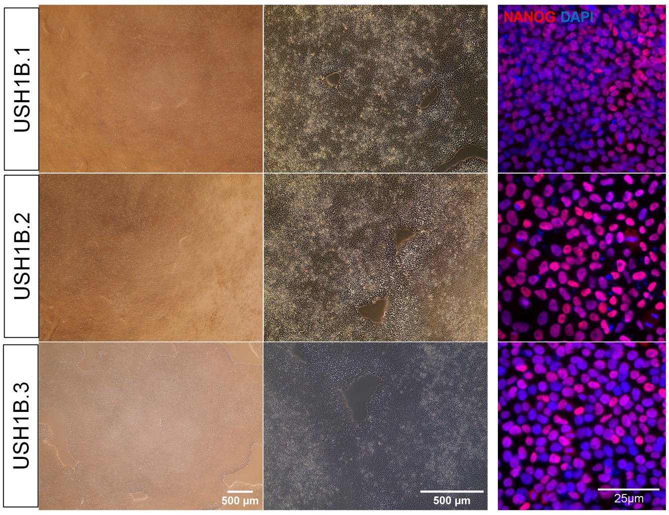

B

iPSC line used in this study	iPSC line name	SNP array (passage number)	Passages used in this study
Control.1	DF19-9-11T.H	43	40-46
Control.2	iPS(IMR90)-4	41	38-42
Control.3	GOS101	24	23-29
Control.4	N7	30	21-34
USH1B.1	Youd6	23	18-24
USH1B.2	N35.1	31	28-31
USH1B.3	GOS102	29	20-26

C

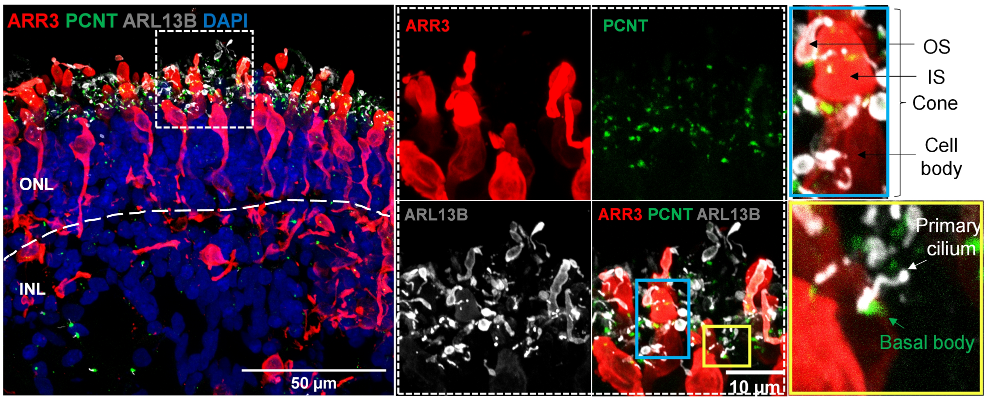

D

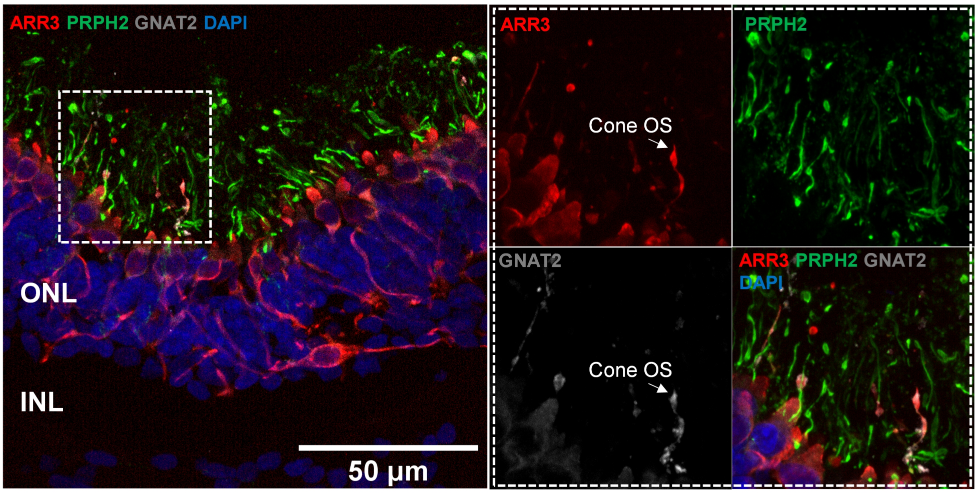

Figure S2 Characterisation of control- and USH1B patient-derived iPSCs and their genome integrity; Subcellular features indicative of photoreceptor maturation. Related to Figure 1 and Figure S1. A) Brightfield images show iPSCs maintained compact cellular morphology with well-defined edges, high nucleus-to-cytoplasm ratio (left and middle columns) and expressed pluripotency marker, NANOG (right column). **B)** Table shows (from first column) nomenclature of all iPSCs used in this study, their original names and source facility where iPSC were generated, passage number when Single Nucleotide Polymorphism (SNP) array analysis was performed, and all passages used in this study. SNP array data available upon request. **C)** Connecting cilium (expressing cilia marker, ARL13B; basal body marker, PCN) was present in photoreceptors. ARL13B also marked the photoreceptor outer segment. ARR3, cone marker. Observed in >3 organoids per iPSC line. **D)** Immunostaining of 28wks retinal organoids for outer segment markers. PRPH2, rod outer segment; GNAT2, cone outer segment. Observed in >3 organoids per iPSC line.

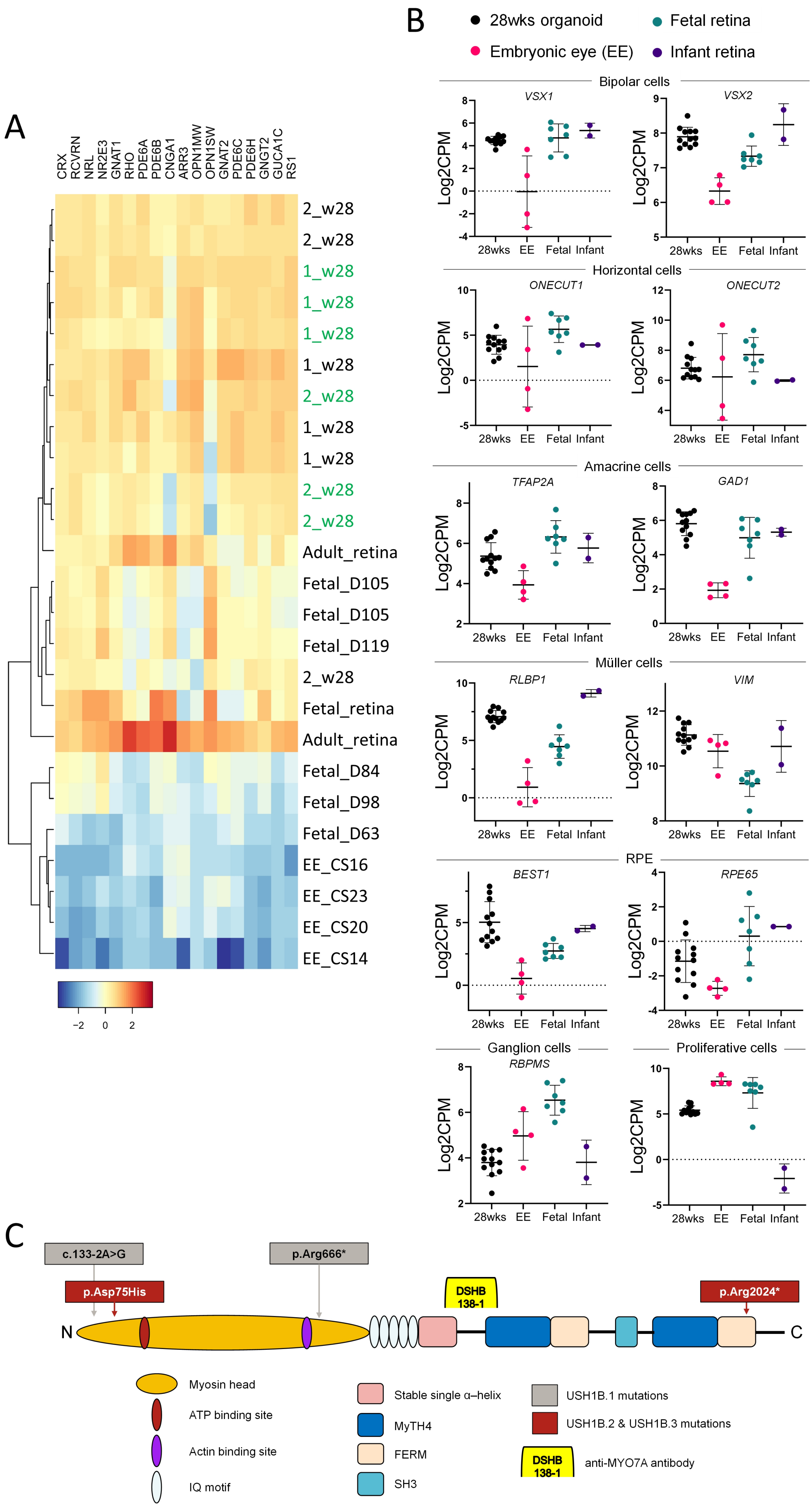

A
CRX
RCVRN
NRL
NR2E3
GNAT1
RHO
PDE6A
PDE6B
CNGA1
ARR3
OPN1MW
OPN1SW
GNAT2
PDE6C
PDE6H
GNGT2
GUCA1C
RS1
2_w28
2_w28
1_w28
1_w28
1_w28
1_w28
2_w28
1_w28
1_w28
2_w28
2_w28
Adult_retina
Fetal_D105
Fetal_D105
Fetal_D119
2_w28
Fetal_retina
Adult_retina
Fetal_D84
Fetal_D98
Fetal_D63
EE_CS16
EE_CS23
EE_CS20
EE_CS14
−2 0 2
B
28wks organoid
Fetal retina
Embryonic eye (EE)
Infant retina
Bipolar cells
VSX1
VSX2
Horizontal cells
ONECUT1
ONECUT2
Amacrine cells
TFAP2A
GAD1
Müller cells
RLBP1
VIM
RPE
BEST1
RPE65
Ganglion cells
RBPMS
Proliferative cells
Log2CPM
28wks EE Fetal Infant
C
c.133-2A>G
p.Arg666*
p.Asp75His
DSHB 138-1
p.Arg2024*
N
C
Myosin head
ATP binding site
Actin binding site
IQ motif
Stable single α–helix
MyTH4
FERM
SH3
USH1B.1 mutations
USH1B.2 & USH1B.3 mutations
DSHB 138-1
anti-MYO7A antibody

Figure S3 28wks retinal organoids were similar to fetal and adult retina based on expression of markers of different retinal cell types. Related to Figure 3 and experimental procedure. A) Heatmap shows expression of photoreceptor markers comparing data from human developing eye (GEO accession: GSE98370, Mellough et al, 2019; encompassing three developmental stages: human embryonic eye (CS14–16), fetal (9 – 17wks), infant (74pcw) and RNA-seq data from 28wks USH1B and control organoids (this study). **B)** Expression of markers of other retinal cell types in human developing eye with USH1B and control organoids (this study). **C)** Schematic of MYO7A shows anti-MYO7A antibody (DSHB, 138-1) binding site used in Western blot shown in Figure 3I-I'. MYO7A contains an N-terminal motor domain, where ATP- and actin-binding sites reside and is connected to a neck region composed of 5 isoleucine-glutamine (IQ) motifs followed by a complex tail region, which binds to cargos or other proteins to form a complex. The tail region begins with a stable single α–helix (SAH) followed by two MyTH4 (myosin tail homology 4)-FERM (four-point1, ezrin, radixin and myosin) regions (MF1 and MF2) sandwiching a SRC homology 3 (SH3) domain (Kabahuma et al., 2021, Heissler and Manstein, 2012).

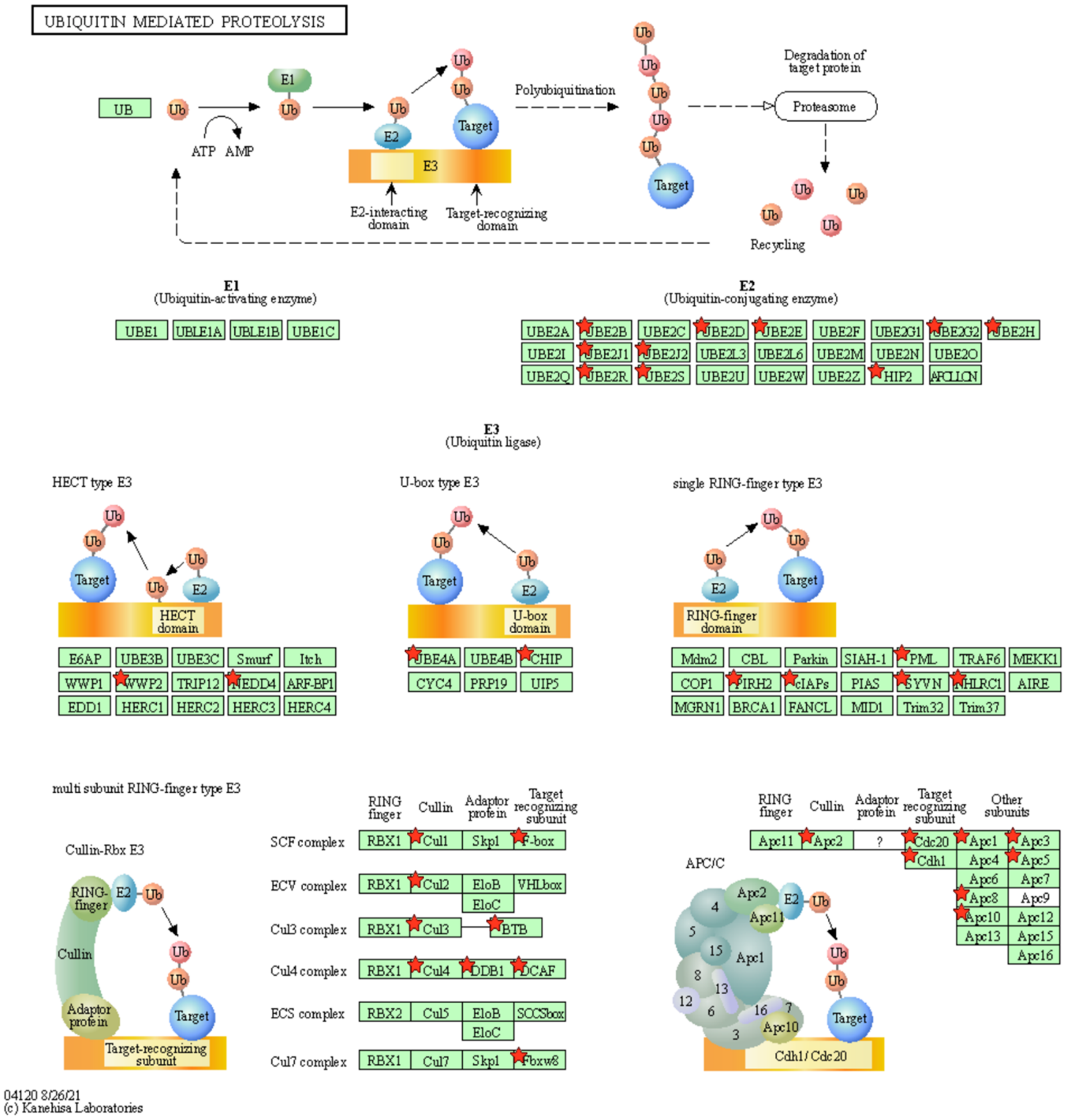

Figure S4 KEGG pathway (04121) ubiquitin mediated proteolysis. Related to Figure 4F. Highlighted in red are the 40 genes in the KEGG ubiquitin mediated proteolysis pathway that were dysregulated in the 28wks USH1B organoids (Benjamini p-value 4.4×10^{-39}; generated using david-d.ncifcrf.gov)

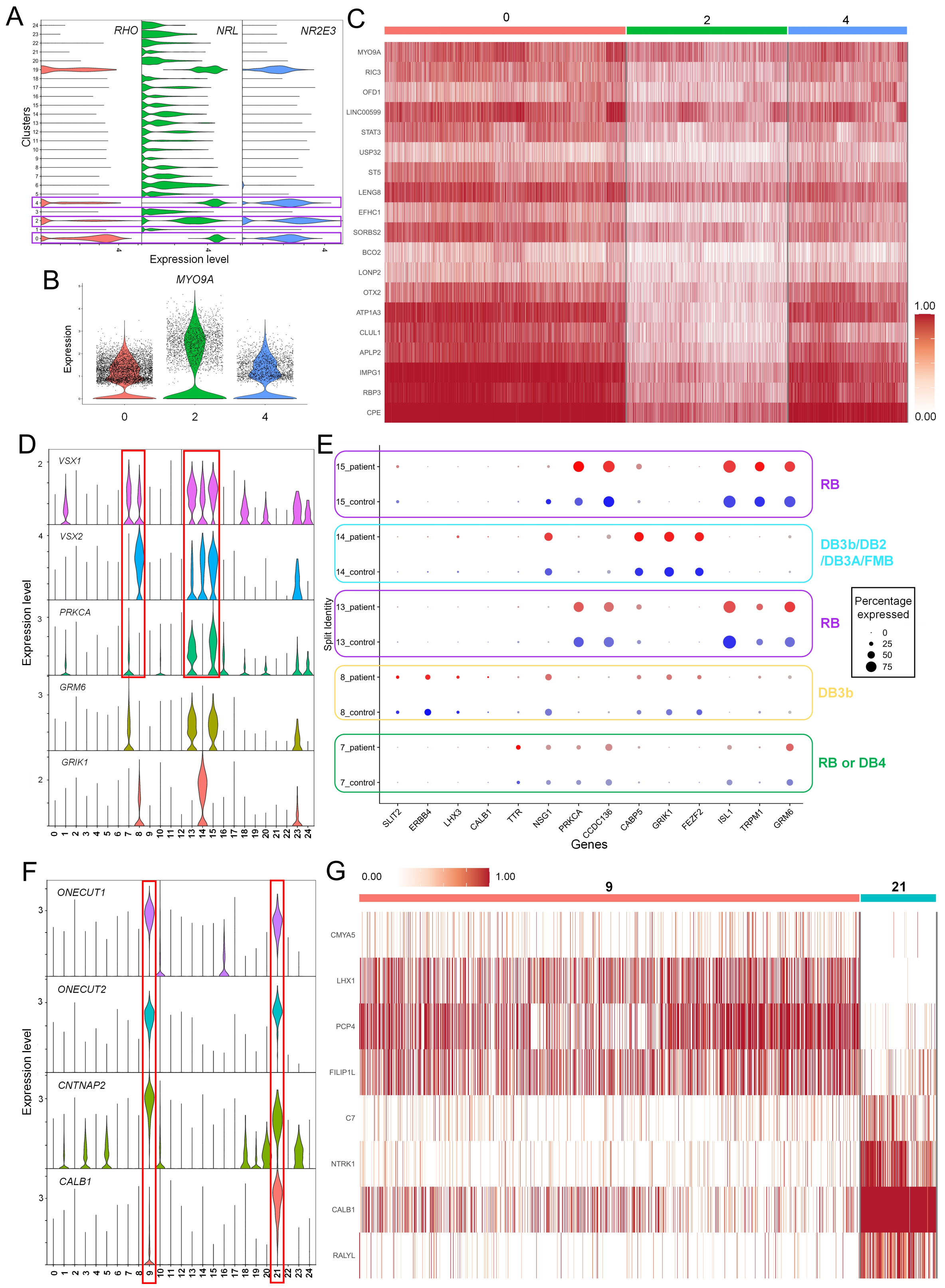

A
RHO
NRL
NR2E3
Clusters
Expression level
B
MYO9A
Expression
0
2
4
C
0
2
4
MYO9A
RIC3
OFD1
LINC00599
STAT3
USP32
ST5
LENG8
EFHC1
SORBS2
BCO2
LONP2
OTX2
ATP1A3
CLUL1
APLP2
IMPG1
RBP3
CPE
1.00
0.00
D
VSX1
VSX2
PRKCA
GRM6
GRIK1
Expression level
E
15_patient
15_control
14_patient
14_control
13_patient
13_control
8_patient
8_control
7_patient
7_control
Split Identity
RB
DB3b/DB2
/DB3A/FMB
RB
DB3b
RB or DB4
Percentage expressed
0
25
50
75
SLIT2
ERBB4
LHX3
CALB1
TTR
NSG1
PRKCA
CCDC136
CABP5
GRIK1
FEZF2
ISL1
TRPM1
GRM6
Genes
F
ONECUT1
ONECUT2
CNTNAP2
CALB1
Expression level
G
0.00
1.00
9
21
CMYA5
LHX1
PCP4
FILIP1L
C7
NTRK1
CALB1
RALYL

Figure S5 The presence of retinal cell subtypes in 35wks retinal organoids. Related to Figure 5. **A)** Rod photoreceptor clusters (0, 2 and 6) were indicated by *RHO*, *NRL* and *NR2E3* expression. **B)** Two rod subtypes described by Yi *et al.* (2021) that can be differentiated by *MYO9A* expression. Cluster 2 expressed high level of *MYO9A* compared to cluster 0 and 4. **C)** Heatmap shows the expression, by cluster 0, 2 and 6, of the most distinctive genes comparing human *MYO9A*$^{+}$ and *MYO9A*$^{-}$ rods identified by Yi *et al.* (2021). **D)** *GRIK1*, *GRM6*, *VSX1*, *VSX2* and *PRKCA* expression identified bipolar cell clusters: 7, 8, 13, 14 and 15; *GRM6* and *GRIK1* expression were indicative of ON- and OFF-bipolar cells, respectively. **E)** More bipolar cell subtypes can be differentiated by expression of markers described by Yi *et al.* (2021). **F)** Horizontal cell clusters were indicated by the expression of *CNTNAP2*, *CALB1*, *ONECUT1* and *ONECUT2:* 9 and 21. **G)** Heatmap shows the expression, by cluster 9 and 21, of the most distinctive genes comparing human H1 and H2 horizontal cells identified by Yi et al. (2021).

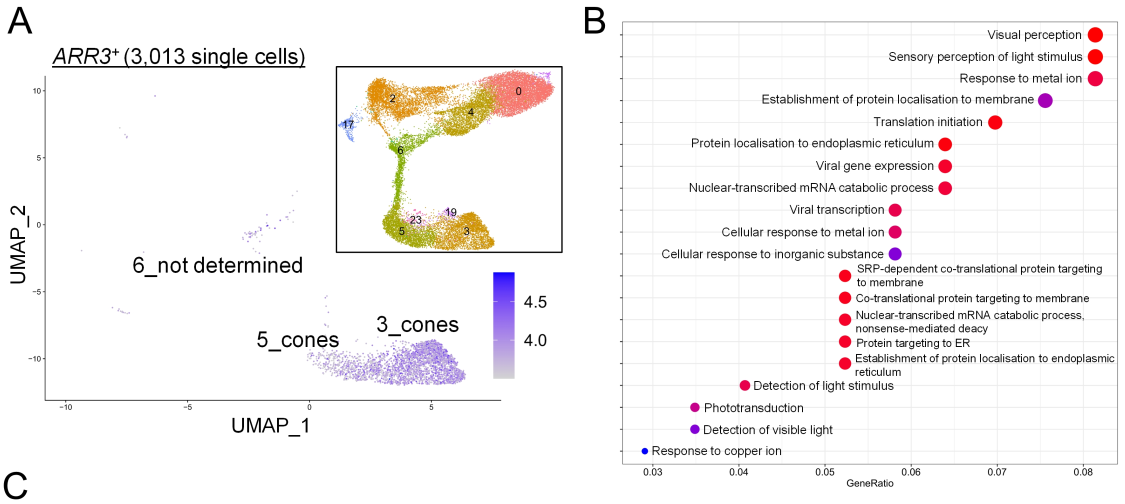

C

ID	Description	qvalue	geneID
GO:0007601	Visual perception	<0.001	CABP2/CNGB3/CPLX4/EYS/GRK7/GUCA1B/NRL/OPN1MW/PDE6G/RBP3/RDH10/RP1L1/RPGRIP1/RS1
GO:0050953	Sensory perception of light stimulus	<0.001	CABP2/CNGB3/CPLX4/EYS/GRK7/GUCA1B/NRL/OPN1MW/PDE6G/RBP3/RDH10/RP1L1/RPGRIP1/RS1
GO:0070972	Protein localization to endoplasmic reticulum	<0.001	ANK2/RPL13A/RPL17/RPL37/RPL41/RPS10/RPS17/RPS21/RPS26/RPS4X/TAPT1
GO:0006413	Translational initiation	<0.001	CSDE1/EIF1AX/EIF2S3/RPL13A/RPL17/RPL37/RPL41/RPS10/RPS17/RPS21/RPS26/RPS4X
GO:0006614	SRP-dependent co-translational protein targeting to membrane	<0.001	RPL13A/RPL17/RPL37/RPL41/RPS10/RPS17/RPS21/RPS26/RPS4X
GO:0006613	Co-translational protein targeting to membrane	<0.001	RPL13A/RPL17/RPL37/RPL41/RPS10/RPS17/RPS21/RPS26/RPS4X
GO:0000184	Nuclear-transcribed mRNA catabolic process, nonsense-mediated decay	0.001	RPL13A/RPL17/RPL37/RPL41/RPS10/RPS17/RPS21/RPS26/RPS4X
GO:0045047	Protein targeting to ER	0.001	RPL13A/RPL17/RPL37/RPL41/RPS10/RPS17/RPS21/RPS26/RPS4X
GO:0072599	Establishment of protein localization to endoplasmic reticulum	0.001	RPL13A/RPL17/RPL37/RPL41/RPS10/RPS17/RPS21/RPS26/RPS4X
GO:0019080	viral gene expression	0.001	CSDE1/JUN/RPL13A/RPL17/RPL37/RPL41/RPS10/RPS17/RPS21/RPS26/RPS4X

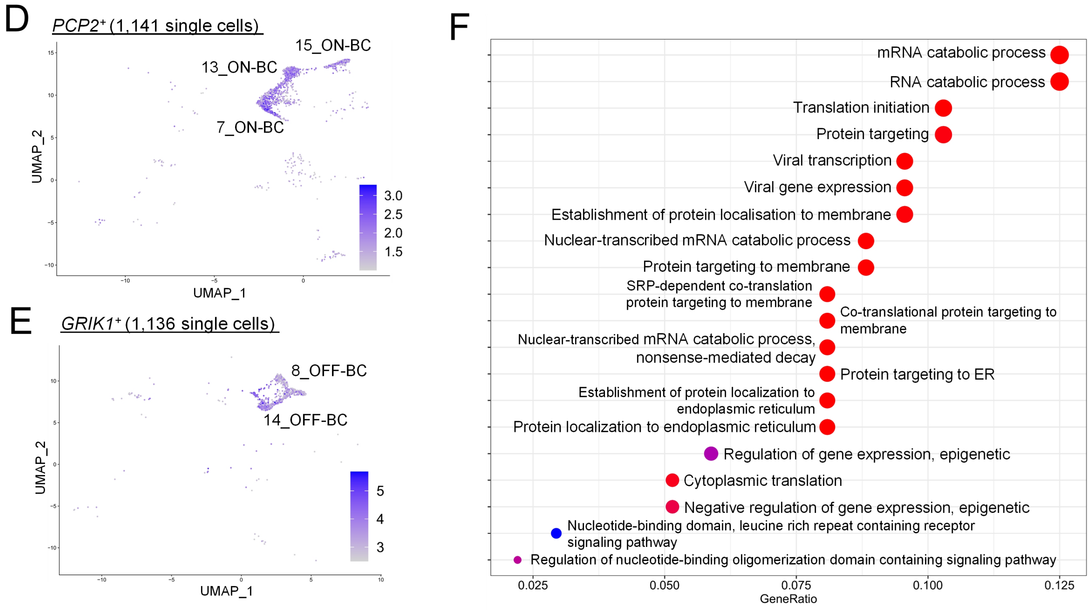

Figure S6 Cell type specific differential expression analysis of single-cell RNA-seq. Related to Figure 6. A) A total of 3,013 cone photoreceptors were isolated based on cone arrestin (*ARR3*) expression. **B)** Top 20 significantly enriched biological processes GO terms by analysis of DEGs between control and USH1B *ARR3*+ single cells. Ranked by gene ratio and q-value. **C)** Details of top 10 significantly enriched biological processes. Ranked by q-value. **D)** A total of 1,136 OFF-BC isolated based on *GRIK1* expression. **E)** A total of 1,141 ON-BC isolated based on *PCP2* expression. **F)** Top 20 significantly enriched biological processes GO terms by analysis of DEGs between control and USH1B *GRIK1*+/*PCP2*+ single cells. Ranked by gene ratio and q-value.

Table S1: Retinal differentiation media. Related to experimental procedures and Figure 1.

Reagent	Volume (mL)	Concentration	Catalogue number
**Embryoid body medium (500 mL)**			
DMEM/F12 (with Glutamax)	up to 500	-	Gibco 10565-018
KOSR	100	20%	Gibco 10828-028
MEM non-EAAs (100X)	5	1x	Gibco 11140-050
β-ME (50 mM)	1	0.1 mM	Gibco 31350-010
**Neural Induction Medium (NIM) (500 mL)**			
Advanced DMEM/F12	up to 500	-	Gibco 12634-010
MEM non-EAAs	5	1x	Gibco 11140-050
N2 supplement	5	1x	Gibco 17502-048
GlutaMax	5	1x	Gibco 35050-038
**Retinal Differentiation Medium (RDM) (500 mL)**			
DMEM (with L-glutamine)	up to 500	-	Gibco 41965-039
F12	150	-	Gibco 21765-029
B27 supplement	10	1x	Gibco 12587-010
**RDM1 (250 mL)**			
RDM	Up to 250	-	
FBS	25	10 %	LifeTech, 10270106
GlutaMax	2.5	1x	Gibco, 35050-038
Taurine	250 µL	100 µM	Sigma, T8691
**RDM2 (50mL)**			
RDM1	50	-	Gibco 10565-018
Retinoic Acid	-	1 µM	Sigma R2625
**RDM3 (50mL)**			
DMEM/F12 with Glutamax	50	-	Gibco 10565-018
Retinoic Acid	-	0.5 µM	Sigma R2625
N2 supplement	500 µL	1x	Gibco 17502-048

Table S2: List of PCR primer and antibodies used for immunohistochemistry and Western blot, related to Figure 1-3 and experimental procedures.

Gene name	Forward primer (5'-3')	Reverse primer (5'-3')		
GAPDH	CACCATCTTCCAGGAGCGAG	GACTCCACGACGTACTCAGC		
RCVRN	CCTCTACGACGTGGACGGTAA	GTGTTTTCATCGTCTGGAAGGA		
NRL	GGGCTGAGTCCTGAAGAGG	TTTAGCTCCCGCACAGACAT		
CRX	GTCCAGGGTTCAGGTTTGGT	GTGCCCGCCTTCCTCTTG		
ARR3	AGTCCTACAGGAGCGACTACT	GGCAGGTTGGTCACCATCTG		
RHO	ACAGGATGCAATTTGGAGGGC	GCTCATGGGCTTACACACCA		
**Antibodies**	**Full protein names**	**Company (catalogue number)**	**Host**	**Dilution**
**Immunohistochemistry**				
CRX	Cone-rod homeobox protein	Abnova (H00001406-M02)	Mouse	1:800
VSX2	Visual system homeobox 2	Merck Millipore (AB9016)	Sheep	1:200
RCVRN	Recoverin	Merck Millipore (A85585)	Rabbit	1:800
PAX6	Paired box protein Pax-6	Biolegend (901301)	Rabbit	1:200
ARR3	Cone arrestin	Novus (NBP-37003)	Goat	1:100
NRL	Neural retina-specific leucine zipper protein	R&D Systems (AF2945)	Goat	1:100
NR2E3	Photoreceptor-specific nuclear receptor	Persus Proteomics (PP-H7223-00)	Mouse	1:500
S-opsin	Short-wave-sensitive opsin 1	Merck Millipore (AB5407)	Rabbit	1:200
L/M-opsin	Medium/Long-wave-sensitive opsin 1	Merck Millipore (AB5405)	Rabbit	1:200
RHO	Rhodopsin	Sigma (O4886)	Mouse	1:800
PRKCA	Protein kinase C alpha type	GeneTex (GTX11723)	Mouse	1:20
ARL13B	ADP-ribosylation factor-like protein 13B	Proteintech (17711-1-AP)	Rabbit	1:500
GNAT2	Guanine nucleotide-binding protein G(t) subunit alpha-2	Santa Cruz Biotechnology (SC390)	Rabbit	1:200
PRPH2	Peripherin-2	Merck Millipore (MABN293)	Mouse	1:200
BRN3A	POU domain, class 4, transcription factor 1	Merck Millipore (MAB1585)	Mouse	1:100
SPY	Synaptophysin	Abcam (AB8049)	Mouse	1:100
TFAP2A	Transcription factor AP-2-alpha	DSHB 3B5	Mouse	1:500
PCNT	Pericentrin	Abcam (AB28144)	Mouse	1:500
Cleaved-CASP3	Cleaved-caspase3	Cell Signalling (9661S)	Rabbit	1:200
γH2AX	Gamma histone H2AX	Abcam (AB2893)	Rabbit	1:400
NQO1	NAD(P)H dehydrogenase [quinone] 1	Abcam (AB2346)	Goat	1:100
BiP/GRP78	Endoplasmic reticulum chaperone BiP	Abcam (AB21685)	Rabbit	1:300
8-OHdG	8-Hydroxy-2'-deoxyguanosine	Abcam (AB48508)	Mouse	1:200
GFAP	Glial fibrillary acidic protein	Abcam (AB5804)	Rabbit	1:200
MYO7A	Unconventional myosin-VIIa	Abcam (Ab3481)	Rabbit	1:200
MYO7A	Unconventional myosin-VIIa	DSHB (138-1)	Mouse	1:200
MYO7A	Unconventional myosin-VIIa	Proteus (256790)	Rabbit	1:200
**Western blot**				
GFAP	Glial fibrillary acidic protein	Abcam (AB5804)	Rabbit	1:1000
ACTB	Actin, cytoplasmic 1	Sigma (A2228)	Mouse	1:5000
MYO7A	Unconventional myosin-VIIa	Abcam (Ab3481)	Rabbit	1:500
MYO7A	Unconventional myosin-VIIa	DSHB (138-1)	Mouse	1:500
MYO7A	Unconventional myosin-VIIa	Proteus (256790)	Rabbit	1:2000

Table S3: Publicly available human embryonic eye and retina RNA-seq data (GEO: GSE98370) (Mellough et al., 2019). Related to Figure 3.

Sample accession	Sample title	Timepoint
SAMN06854090	12301 Embryonic CS14 Eye RNA	CS14
SAMN06854087	12556 Embryonic CS16 Eye RNA	CS16
SAMN06854084	12527 Embryonic CS20 Eye RNA	CS20
SAMN06854081	1901 Embryonic CS23 Eye RNA	CS23
SAMN06854080	13149 Foetal 1 Retina RNA	63days
SAMN06854077	1940 Foetal 4 Retina RNA	84 days
SAMN06854074	12552 Foetal 6 Retina RNA	98 days
SAMN06854071	12773 Foetal 7 Retina RNA	105 days
SAMN06854070	12xxx1 Foetal 7 Retina RNA	105 days
SAMN06854064	13172 Foetal 9 Retina RNA	119 days
SAMN06854091	191115 Adult Retina RNA	74pcw
SAMN06854092	010813 Adult Retina RNA	77pcw

Table S4: Bulk RNA-seq analysis. Related to Figure 3 and 4.

Table S5: Single cell RNA-seq analysis. Related to Figure 5 and 6.

Supplemental Experimental Procedures

Generation and maintenance of iPSCs

The study was approved by the National Research Ethics Committee London-Dulwich (11/LO/1243). Skin biopsies were collected from consenting patients or guardians of patients with a clinical genetic diagnosis of USH1B who attended clinics at Great Ormond Street Hospital (GOSH) or unaffected individuals as controls. Fibroblasts from patient 1 (USH1B.1), patient 2 (USH1B.2) and patient 3 (USH1B.3) were reprogrammed to iPSC using Sendai virus by the HipSci (the human Induced Pluripotent Stem Cell Initiative funded by the Wellcome Trust and MRC, www.hipsci.org), NIHR Cambridge Biomedical Research Centre human iPSCs core facility and NIHR GOSH Biomedical Research Centre iPSC facility, respectively. Control.3 iPSC was generated through the same route as USH1B.3, Control.4 iPSC was generated through the same route as USH1B.2, and control.1 (DF19-9-11T.H) and control.2 (iPS(IMR90)-4) were purchased from WiCell. All iPSCs were cultured on Vitronectin XF (Stem Cell Technologies, 100-0763) with Essential 8 medium (Gibco, A1517001). iPSC lines maintained a healthy morphology and growth rate, and expressed pluripotency markers OCT4, SOX2, TRA-1-60 and NANOG. *MYO7A* gene mutations were confirmed in patient iPSCs with Sanger sequencing. Details of mutations are given in Figure S1. iPSC cultures displayed a normal growth rate and morphology and maintained pluripotency marker expression. Single- nucleotide polymorphism (SNP) karyotype analysis of the iPSC lines and passage numbers used are given in Figure S2. SNP microarray analysis was performed using HumanCytoSNP-12 v2.1 BeadChip Kit from Illumina and Genomestudio 2.0 confirmed the edited line retained a normal digital karyotype (Data available upon request).

Immunofluorescence and microscopy

Retinal organoids were fixed with 4% PFA solution (Thermo Scientific, 28908) in phosphate buffer saline (PBS, Oxoid BR0014G) for 30 min at room temperature. Fixed organoids were washed thrice with PBS and incubated overnight in 30% sucrose (Sigma, S0389) solution in PBS (or in 7.5% gelatin/5% sucrose in PBS for connecting cilium analysis) before embedding in OCT (Fisher Scientific, 12678646) and stored at -80°C. Cryosections of frozen organoids were generated using a Leica cryostat and collected on microslide superfrost plus blue slides (VWR, 630-0950). For immunostaining, cryosections were left at room temperature for 30 min, washed once with PBS and incubated with blocking solution 10% donkey serum (Sigma, D9663), 1% (wt / vol) bovine serum albumin (Merck, A7906), 0.5% Triton X-100 (Merck, 112298) prepared in PBS for 1 h at room temperature. Primary and secondary antibodies were prepared in the same buffer containing 3% donkey serum, 1% BSA, 0.5% Triton X-100 in PBS. Sections were incubated at room temperature overnight in humidified chamber with primary antibodies, washed thrice with 0.1% TWEEN 20 (Sigma, P1379) in PBS, 15 min per wash, followed by incubation with secondary antibodies. After two washes with 0.1% TWEEN 20 in

PBS and one wash with PBS, sections were counterstained with DAPI, before mounting coverslips with ImmunoFluoroMount (GTX30928). Microscopy was performed using Zeiss LSM 710 confocal microscope. Image analysis was performed using FIJI (Schindelin et al., 2012). All primary and secondary antibodies are listed in the Table S2.

Quantification of organoid size and thickness of outer nuclear layer (ONL)

Organoid size was quantified from Fiji image analysis using 7wks organoid size as baseline because at 6wks, adherent organoids were micro-dissected and transferred to 3D suspension culture and at 7wks, medium was supplemented with fetal bovine serum, after which an increase in organoid size was observed. ONL thickness was quantified using Fiji with the areas measured guided by ARR3 (cone arrestin, cone photoreceptor) or RCVRN (recoverin, pan-photoreceptor) IHC plus DAPI counterstain in retinal cryosections.

RT-PCR and qRT-PCR

RNA extraction was performed using miRNeasy micro kit (Qiagen, 217084) and cDNA was synthesised using miScript II RT kit (Qiagen, 218160). RNA was quantified using Thermo Scientific™ NanoDrop™ One Microvolume UV-Vis Spectrophotometer (13-400-518). RT-PCR was performed using MyFi™ mix (Meridian Bioscience, BIO-25049) and PCR products were resolved in UltraPure™ agarose gel (Thermo Scientific, 16500100) stained by SYBR® Safe DNA Gel Stain (Thermo Scientific, S33102). For qRT-PCR, SYBR green PCR master mix (Life Technologies, 4309155) was used, and reactions were analysed with Applied Biosystem 7500 Real Time PCR system. Technical triplicates of independent samples were prepared. Results were normalised to GAPDH (internal control) and fold change was calculated using the $2^{-\Delta\Delta Ct}$ method. The sequences of primers used can be found in the Table S2.

Transmission electron microscope (TEM)

Retinal organoids were fixed with 3% EM grade glutaraldehyde in 0.1 M sodium cacodylate buffer and pH 7.4 5 mM CaCl2 for 15 min at room temperature and kept at 4°C before processing. Organoids were processed at the Institute of Neurology electron microscopy facility, UCL. TE micrographs were captured using a JEOL JEM-1400 120 kV transmission electron microscope.

Western blot analysis

Protein extraction was performed using 1x RIPA Buffer (Cell Signalling Technology, 9806) supplemented with 1x protease inhibitor cocktail (Sigma-Aldrich, P8340). Protein concentration was determined using a Pierce™ BCA Protein Assay Kit (Thermo Scientific, 23225) and analysed by Infinite 200 PRO NanoQuant microplate readers (Tecan). Western blotting was performed with 20 µg protein per sample. Polyvinylidene difluoride (PVDF) membrane (GE Healthcare, 10600023) was blocked with 5% non-fat milk (Sigma) overnight

at 4°C before staining with primary antibodies (prepared in 5% non-fat milk) overnight at 4°C on tube rollers. PVDF membrane was washed thrice with Tris-buffered saline (TBS)-tween 20 (TBST) (TBS and 0.5% Tween 20) solution (15 minutes per wash) before incubation with secondary antibodies (prepared in TBST) for 2 h at room temperature on shaker. Visualisation was performed with the Odyssey® CLx Infrared Imaging System (LI-COR Biosciences). Immunoblotting of MYO7A was performed using, with DSHB 138-1 anti-MYO7A antibody.

Bulk RNA-sequencing

Total RNA was extracted from retinal organoids and human neural retinal tissue using a miRNeasy micro kit (Qiagen, 217084). Human tissue was obtained from the Joint Medical Research Council UK (grant G0700089)/Wellcome Trust (grant GR082557) Human Developmental Biology Resource (http://www.hdbr.org/) and human adult eyes from Moorfields Biobank with ethics approval. RNA integrity and quantity were tested with an Agilent 4200 Tapestation System. A total of 100 ng RNA was processed using the KAPA mRNA HyperPrep Kit (Roche, KK8580) according to manufacturer's instructions. In brief, poly-adenylated mRNAs were isolated, chemically fragmented and primed with random hexamers, before generation of strand-specific first strand cDNA using reverse transcriptase in the presence of actinomycin D. Quality of libraries generated was accessed using High Sensitivity Agilent DNA 1000 assay (Agilent, 5067-4626)) on Agilent TapeStation 4200, before being enzymatically normalised using a Normalase assay (Swift BioSciences, 66096). Libraries were sequenced on a S1 flow cell on a NovaSeq 6000 system (Illumina, San Diego, US) at 0.8 nM using v1.5 SBS chemistry and a 50bp paired-end recipe. Approximately 20 million reads per sample were generated. Data was demultiplexed and FASTQ files generated using Illumina's bcl2fast Conversion Software v2.20. FASTQ files were then tagged with the UMI read (with UMITools) and aligned to the human genome UCSC hg38 using RNA-STAR 2.5.2b. Aligned reads were UMI deduplicated using JE-Suite (1.2.1) and reads per transcript were counted by FeatureCounts to produce a digital output of gene expression. EdgeR was used to generate counts per million (CPM), to be used for filtering lowly expressed genes. Differential expression analysis was performed using limma-voom with cut-off of adjusted p value < 0.05 for statistical significance. Package clusterProfiler (version 3.0.4) was used for GO enrichment analysis.

Single-cell RNA sequencing

Single cell RNA libraries were generated using the Chromium Single Cell 3' Reagent Kits v3 (10X Genomics, CA, USA). The cells were counted using a trypan blue exclusion count assay with a Bio-Rad Automated Cell Counter TC10 and diluted for loading onto the Chromium Controller. Loading was performed to target capture of ~8,000 Gel Bead-In-EMulsions (GEMs) per sample for downstream analysis, and samples were processed through the Chromium

Controller following the standard manufacturer's specifications. The sequencing libraries were evaluated for quality on the Agilent TapeStation (Agilent Technologies, USA), and quantified by using a Qubit 2.0 Fluorometer (Invitrogen) and pooled libraries were quantified using qPCR (Applied Biosystems) prior to loading onto an Illumina sequencing platform. The samples were sequenced at a configuration compatible with the recommended guidelines as outlined by 10X Genomics. Raw sequence data (.bcl files) generated from Illumina HiSeq was converted into fastq files and demultiplexed using the 10X Genomics' cellranger mkfastq command. Subsequent UMI and cell barcode de-convolution along with mapping to the respective genome was performed using 10X Genomics' cellranger software package to generate the final digital gene expression matrices.

Data was analysed using the Seurat package. Only cells expressing more than 200 genes and fewer than 8000 and with mitochondrial gene percentages less than 30% were kept for further analysis. In total, 19,399 and 20,535 single cells from two independent controls and from three independent USH1B patients, respectively, remained (control.1 replicate1, 6,607 cells; control.1 replicate 2, 6,315 cells; control.3, 6,477 cells; USH1B.1, 6,653; USH1B.2, 6,983 cells and USH1B.3, 6,899 cells). A total of 39934 cells were integrated with the Seurat standard protocol and the effect of mitochondrial genes were regressed out using "ScaleData" function. The top markers for each cluster were identified using the "FindConservedMarkers" function. Cell identity assignment was performed based on well-defined retinal markers and two recent single-cell sequencing studies of human retina. Full list of top marker genes used for cell assignment can be found in Table S. The "FindMarkers" function was used to identify differentially expressed genes between control and patient cells globally or in a cell/cluster-specific manner.

Data availability

RNA-seq data have been deposited in ArrayExpress under accession numbers E_MTAB-11405 (Bulk RNA-seq) and E-MTAB-11990 (single cell RNA-seq)

Supplemental References

AILION, M., HANNEMANN, M., DALTON, S., PAPPAS, A., WATANABE, S., HEGERMANN, J., LIU, Q., HAN, H. F., GU, M., GOULDING, M. Q., SASIDHARAN, N., SCHUSKE, K., HULLETT, P., EIMER, S. & JORGENSEN, E. M. 2014. Two Rab2 interactors regulate dense-core vesicle maturation. *Neuron,* 82**,** 167-80.

BRASIL, A. A., DE CARVALHO, M. D. C., GERHARDT, E., QUEIROZ, D. D., PEREIRA, M. D., OUTEIRO, T. F. & ELEUTHERIO, E. C. A. 2019. Characterization of the activity, aggregation, and toxicity of heterodimers of WT and ALS-associated mutant Sod1. *Proc Natl Acad Sci U S A,* 116**,** 25991-26000.

BRUGGER, M., BECKER-DETTLING, F., BRUNET, T., STROM, T., MEITINGER, T., LURZ, E., BORGGRAEFE, I. & WAGNER, M. 2021. A homozygous truncating variant in CCDC186 in an individual with epileptic encephalopathy. *Ann Clin Transl Neurol,* 8**,** 278-283.

DAS, A., QIAN, J. & TSANG, W. Y. 2017. USP9X counteracts differential ubiquitination of NPHP5 by MARCH7 and BBS11 to regulate ciliogenesis. *PLoS Genet,* 13**,** e1006791.

ESTEVE, P., SANDONIS, A., CARDOZO, M., MALAPEIRA, J., IBANEZ, C., CRESPO, I., MARCOS, S., GONZALEZ-GARCIA, S., TORIBIO, M. L., ARRIBAS, J., SHIMONO, A., GUERRERO, I. & BOVOLENTA, P. 2011. SFRPs act as negative modulators of ADAM10 to regulate retinal neurogenesis. *Nat Neurosci,* 14**,** 562-9.

HEISSLER, S. M. & MANSTEIN, D. J. 2012. Functional characterization of the human myosin-7a motor domain. *Cell Mol Life Sci,* 69**,** 299-311.

HUAI, J. & ZHANG, Z. 2019. Structural Properties and Interaction Partners of Familial ALS-Associated SOD1 Mutants. *Front Neurol,* 10**,** 527.

JIANG, K., FAIRLESS, E., KANDA, A., GOTOH, N., COGLIATI, T., LI, T. & SWAROOP, A. 2020. Divergent Effects of HSP70 Overexpression in Photoreceptors During Inherited Retinal Degeneration. *Invest Ophthalmol Vis Sci,* 61**,** 25.

JIN, T., TAN, X., SHI, X., LV, L., PENG, X., ZHANG, H., TANG, B., WANG, C. & YANG, M. 2021. Preliminary Findings on Proline-Rich Protein 14 as a Diagnostic Biomarker for Parkinson's Disease. *Neuromolecular Med,* 23**,** 285-291.

KABAHUMA, R. I., SCHUBERT, W. D., LABUSCHAGNE, C., YAN, D., BLANTON, S. H., PEPPER, M. S. & LIU, X. Z. 2021. Spectrum of MYO7A Mutations in an Indigenous South African Population Further Elucidates the Nonsyndromic Autosomal Recessive Phenotype of DFNB2 to Include Both Homozygous and Compound Heterozygous Mutations. *Genes (Basel),* 12.

LENASSI E, SAIHAN Z, CIPRIANI V, LE QUESNE STABEJ P, MOORE AT, LUXON LM, BITNER-GLINDZICZ M, WEBSTER AR. 2014. Natural history and retinal structure in patients with Usher syndrome type 1 owing to MYO7A mutation. *Ophthalmology*, 121(2), 580-7.

MACKAY, E. W., MOLERO, S. I., TIRATHDAS, L. H., PETERSON-MADURO, J., ZANG, J., NEUHAUSS, S. C. F., SCHULTE-MERKER, S., WILSON, S. W. 2020. Zinc dysregulation in slc30a8 (znt8) mutant zebrafish leads to blindness and disrupts bone mineralisation. *bioRxiv preprint*.

MOLLEMA, N. J., YUAN, Y., JELCICK, A. S., SACHS, A. J., VON ALPEN, D., SCHORDERET, D., ESCHER, P. & HAIDER, N. B. 2011. Nuclear receptor Rev-erb alpha (Nr1d1) functions in concert with Nr2e3 to regulate transcriptional networks in the retina. *PLoS One,* 6**,** e17494.

NGUYEN, T. T. M., MURAKAMI, Y., SHERIDAN, E., EHRESMANN, S., ROUSSEAU, J., ST-DENIS, A., CHAI, G., AJEAWUNG, N. F., FAIRBROTHER, L., REIMSCHISEL, T., BATEMAN, A., BERRY-KRAVIS, E., XIA, F., TARDIF, J., PARRY, D. A., LOGAN, C. V., DIGGLE, C., BENNETT, C. P., HATTINGH, L., ROSENFELD, J. A., PERRY, M. S., PARKER, M. J., LE DEIST, F., ZAKI, M. S., IGNATIUS, E., ISOHANNI, P., LONNQVIST, T., CARROLL, C. J., JOHNSON, C. A., GLEESON, J. G., KINOSHITA, T. & CAMPEAU, P. M. 2017. Mutations in GPAA1, Encoding a GPI Transamidase Complex Protein, Cause Developmental Delay, Epilepsy, Cerebellar Atrophy, and Osteopenia. *Am J Hum Genet,* 101**,** 856-865.

SANTOFIMIA-CASTANO, P., LAN, W., BINTZ, J., GAYET, O., CARRIER, A., LOMBERK, G., NEIRA, J. L., GONZALEZ, A., URRUTIA, R., SOUBEYRAN, P. & IOVANNA, J. 2018. Inactivation of NUPR1 promotes cell death by coupling ER-stress responses with necrosis. *Sci Rep,* 8**,** 16999.

SCHINDELIN, J., ARGANDA-CARRERAS, I., FRISE, E., KAYNIG, V., LONGAIR, M., PIETZSCH, T., PREIBISCH, S., RUEDEN, C., SAALFELD, S., SCHMID, B., et al. 2012. Fiji: an open-source platform for biological-image analysis. *Nat Methods,* 9**,** 676-82.

SHINDE, V., PITALE, P. M., HOWSE, W., GORBATYUK, O. & GORBATYUK, M. 2016. Neuronatin is a stress-responsive protein of rod photoreceptors. *Neuroscience,* 328**,** 1-8.

TELIAS, M., DENLINGER, B., HELFT, Z., THORNTON, C., BECKWITH-COHEN, B. & KRAMER, R. H. 2019. Retinoic Acid Induces Hyperactivity, and Blocking Its Receptor Unmasks Light Responses and Augments Vision in Retinal Degeneration. *Neuron,* 102**,** 574-586 e5.

YASUDA, M., TANAKA, Y., OMODAKA, K., NISHIGUCHI, K. M., NAKAMURA, O., TSUDA, S. & NAKAZAWA, T. 2016. Transcriptome profiling of the rat retina after optic nerve transection. *Sci Rep,* 6**,** 28736.
